# Supplementary material for: Student behavior at university: The development and validation of a 10-dimensional scale
Source: PLoS One. 2024 Nov 15;19(11):e0313357. doi: 10.1371/journal.pone.0313357 (PMC11567547; doi:10.1371/journal.pone.0313357)
Supplement: S1 File — (DOCX) [file pone.0313357.s001.docx]

S1 File. Procedures of the collection and analysis of the interview data (qualitative phase of the study), and the generation of initial items for the student behavior questionnaire

***Data***

The data were collected in the Fall 2022 through in-depth semistructured interviews with first-year students enrolled in undergraduate programs of eight Russian highly selective universities. Two universities were located in Northwestern federal district, two - in Siberian federal district, one university each in the Far Eastern, Volga, Central and Ural federal districts. All universities are participants of the “Priority 2030” program: six universities - in the “Territorial and (or) Industry Leadership” track, two universities - in the “Research Leadership” track. Participants were recruited via emails, the social network Vkontakte, and Telegram messenger. In total, 119 first-year students participated in the qualitative phase of this study, 60 of them were males and 59 - females; 35 students enrolled in computer science, 18 - in engineering, 27 - in science majors, 26 - in humanities, and 13 - in social sciences. All participants signed a consent form.  They participated in the project voluntarily and did not receive any incentives. Interviews were conducted in online mode and lasted between 27 and 93 minutes.

There were eleven interviewers, ten women and one man. All of them study in social science programs or work at university as research assistants, analysts or researchers. Also, all of them had previous experience of taking interviews in qualitative research projects. Before the interview, the research team conducted two meetings with interviewers to explain interview protocol, recruiting strategy, interview procedure, as well as to make sure that each interviewer has the same understanding of the purpose of the study and the questions included in the interview protocol.

The interview protocol was designed to cover student previous educational experience and family characteristics, expectations from study at university, daily learning activities and perceptions of first week at university as well as strategies, which students plan to apply at university to pass exams, communicate with classmates and faculty and combine curricular activities with extracurricular activities at university and life outside university.

***Analysis of the interview data***

The current study utilises thematic analysis strategy. As the number of interviews exceeds 100, we employ the three-step strategy to work with data. In the first step, we randomly selected 20 interviews to develop a code system. Each member of the research team (including three researchers with a doctoral degree in education or sociology) read these interviews and suggest their own code system. In discussion, on the basis of these systems, the merged code system was developed. The unit for analysis was a theme, for example, “tendency to act according to faculty expectations” or “willingness to receive the highest grade”. All citations covering these themes were coded in a certain way, and then were related to theoretical concepts, which can explain the described behavioral acts, perception or attitude, for example, conformity or motivation. In the second step, the developed code system was applied for analysis of the remaining 99 interviews. If we were meeting with new themes in these interviews, the developed code system was updated during the next round of discussion. On the third step, all codes were divided into two groups: 1) types of student behavior at university and 2) various characteristics of student and university experience that can influence student behavior. Thus, if a participant tell to an interviewer that he/she do not participate in student clubs and extracurricular events because he/she is shy and scared, because it is something unusual for him/her, something that he/she has never done before, we coded his/her behavior as non-participation in extracurricular activities, and, as possible factors considered the fear of new experience. To develop the items for the survey instrument, we focused on the first group of codes related to student behavior types. However, in the qualitative phase of study, we also observed the important role of motivation in shaping student behavior. The theoretical assumptions about the relations between student motivation and behavior was utilized to test construct-related validity of the instrument.

Table 1. Examples of developing items based on the results of interview data analysis

| Developed items | Citations from interview with students |
| --- | --- |
| *Academic diligence* | |
| In class, tried to take detailed notes so as not to forget important information | *“I took notes of all the lectures that I attended, but I also took notes on the ones that I missed”.* (Interview 11, female student from university 5 enrolled in social sciences) |
| Tried to constantly and carefully listen to an instructor in class so as not to miss anything important | *“Some instructors are boring, but studying is interesting. And, it is not about me to sit in class yawning and not understand anything. I do my best to prevent myself from this”.* (Interview 50, male student from university 5 enrolled in engineering) |
| Completed all homework thoroughly throughout the study week | *“It is necessary for me to read all the information that is given by instructors, to prepare for all classes during the study week. It's hard work. I need to work like a dog”.* (interview 57, male student from university 1 enrolled in social sciences) |
| Even if the course was not interesting to you, still forced yourself to learn the material and complete assignments | *“One day I can be very interested in learning, and on another day it can be somewhat boring. But, in general, I always try to work, even, sometimes, I need to overcome myself in order to get involved in the learning process”.* (Interview 74, male student from university 8 enrolled in computer science) |
| Paid attention to how your learning is organized and how to make it most effective (planned the time and chose specific places for doing homework) | *“If on the lecture an instructor explains course material poorly, then I would prefer do not attend these lectures and learn this material at home, because it will more easy way for me to understand this material”* (Interview 97, male student from university 3 enrolled in engineering) |
| Tried to learn and review course material in a timely manner, and not put everything off until the last minute | *“It is important for me to not accumulate study debts. Some students don’t understand this, they say why are you trying to fit into this schedule if instructors allow you to submit completed assignments later. But it is the wrong way, you would accumulate these incomplete assignments. I try to study, understand, and submit everything on time, so that there are no questions from instructors to me. For example, last weekend I did homework on history. I will need it on Wednesday, and I will take my notebook on Wednesday and go to class. I won’t think on Tuesday night that “Damn it, I still have to do my homework”.* (Interview 54, female student from university 6 enrolled in computer science) |
| Invested a lot of time and effort to complete an assignment that you could not done the first time | *“I'm dyslexic. I have very big problems with reading aloud. I read slowly. There was an assignment at an exam in the format of an oral interview where we needed to read the text. It was mentioned that it is very easy, and everyone will complete this assignment without additional preparation. But I understood that I would not be able to read this text in two minutes if I did not prepare. I had this book for the oral interview, and I read it every day taking into account the time spent on this and my progress in completing this assignment.  It was really very difficult and stressful for me. And when I got 20 points out of 22, it was just wow”.* (Interview 29, male student from university 7 enrolled in social sciences) |
| Coming home after classes, tried to review the covered course material and comprehend it | *“I come home at 6 p.m. Until about 7 p.m., I have time for dinner and rest. Then I begin to study. For example, if the mathematical analysis is scheduled tomorrow, I read my notes to remember the material, which were covered in the last class, and review this material”.* (Interview 73, male student from university 4 enrolled in computer science) |
| Coming home after classes, looked for additional sources of information (for example, lectures on YouTube) to better understand the course material | *“Then I search the material on this topics [covered in class] in the Internet, how they are explained on educational websites, in videos on YouTube”* (Interview 73, male student from university 4 enrolled in computer science) |
| Did extra work other than homework to ensure you have a good understanding of the course material | *“It is very important for me to absorb all the information that an instructor gives us. All additional books and internet sources. I always try to write everything down, attend all classes and read more materials than are covered in class”.* (Interview 100, female student from university 8 enrolled in science) |
| Skipped classes | *“Sometimes is skip classes on Saturday because I’m exhausted after study on Friday”* (Interview 107, male student from university 7 enrolled in computer science) |
| Came to class unprepared | *“I often neglect my homework. I rarely do it. And this is a reason why now I have serious problems with my grade points”.* (Interview 112, male student from university 8 enrolled in humanities) |
| Did things other than your studies during class | *“If a lecture is boring, we play chess, or do some bullshit things during it”.* (Interview 40, female student from university 5 enrolled in science) |
| *Active learning* | |
| Answered instructor’s questions or show work on an assignment at the board | *“I often volunteer to do study work at the board. It is a good way for me to understand course material”*. (Interview 102, male student from university 8 enrolled in computer science) |
| Did a group assignment, participated in group work in class | *“On seminars in history, we create study groups and work on group assignments, which later we present in class. To complete these assignments, we need knowledge in the subject”.* (Interview 106, male student from university 7 enrolled in computer science) |
| Asked an instructor / teacher assistant to help you when you could not understand some course material on your own | *“If I don’t understand, I ask instructors to explain to me some materials or how to complete a difficult assignment in their additional office hours. And they help me”.* (Interview 97, male student from university 3 enrolled in engineering) |
| *Social integration* | |
| Spent time free from studies and university activities with your classmates | *“After classes, I spend time with my classmates. We go to watch cartoons or dance, or something else”.* (Interview 114, female student from university 5 enrolled in humanities) |
| Tried to meet new interesting people at an university, looked for opportunities for this | *“I have already met a lot of interesting guys, and am going to meet more interesting people. My strategy is the following: more interesting people and interesting things that happen around me. You meet people and interesting things somehow begin to appear around you”.* (Interview 78, male student from university 1 enrolled in computer science) |
| Chatted with classmates in a group chat | *[About communication with classmates at the beginning of the study at university]: “and you type in the group chat a message with questions: how can I solve this problem? how can I find the program of the lecture? what should I do with it? And you, with such big eyes, run to each other with questions”.* (Interview 29, male student from university 7 enrolled in social sciences) |
| Communicated with classmates during breaks between classes | *“If we have a break between classes, I have lunch together with classmates and talking with them”* (Interview 114, female student from university 5 enrolled in humanities) |
| Asked classmates for help on academic issues | *“I always do my homework. However, sometimes it is too difficult. And I ask my classmates for help with it”* (Interview 87, male student from university 8 enrolled in science) |
| *Conformity behavior* | |
| Tried to behave in class in such a way as to show the teacher a good side | *“In classes, I try to show myself as a diligent student. It doesn’t always work out, I don’t always understand everything. <...> I try to answer when someone doesn’t answer. I try to show my best side.”* (Interview 87, male student from university 8 enrolled in science) |
| Agreed with what an instructor said, even if you thought that he/she was wrong | *“This is an old man and he is from the old school of teaching, i.e. he gives some material, and we must learn it word for word, as he gave it. I don’t like this at all, because I don’t see the point in simple cramming. If I learn it, it doesn't mean I understand the meaning. Why not just retell his material briefly and clearly, the meaning will not change. But demands are demands, and I did it”.* (Interview 79, female student from university 4 enrolled in science) |
| In class, tried to behave in a such way to demonstrate that you are good in course material while it was not true | *“Interviewer: Are you active in class?*  *Participant: Yes, very often. I am trying really hard to show that I am smart. Even if my knowledge is poor, I try to be active.”* (Interview 79, male student from university 2 enrolled in science) |
| *Extracurricular involvement* | |
| Attended student clubs organized at an university | *“Next year, I want to join some club, it would be interesting. I formed the board games club here. It is too loud to call it “a club”, but it is some student activity at least”.* (Interview 107, male student from university 7 enrolled in computer science) |
| Participated in a research, applied or creative project together with other university students | *“Recently, I took part in an engineering championship. To participate in this event, I needed a team. I invited my old friends who study here and were able to develop a project for this competition. Basically, I was the leader and captain of the team and organized the activities. Not everything is perfect as I wanted, but next time I will take into account all my mistakes”* (Interview 47, male student from university 2 enrolled in engineering) |
| Attended university extracurricular events (concerts, public lectures, workshops, etc.) | *“I go to some interesting events at universities, like theatrical performances, movies <...> There were a design festival and a book festival just recently”* (Interview 1, female student from university 4 enrolled in humanities) |
| Participated in meetings of student organizations | *“Once in a week I participate in the meetings of our student organization. When the festival of student organizations took place in my city, I join one of them. Since then, I attend the meeting of this organization each Wednesday after classes in physical training”.* (Interview 101, female student from university 8 enrolled in humanities) |
| Were engaged in organizing of university events | *“I went to the “studhouse”. It is on the campus of our university, a special place for extracurricular activities. The guys and I spent 2 hours preparing the rooms and props for a quest. At 3 p.m. we started the event, which lasted about 2.5 hours. It was very interesting and fun. This was my first time participating in organizing an event for students like me”.*  (Interview 30, female student from university 1 enrolled in humanities) |
| Were engaged in volunteering as part of university initiatives | *“I want to participate in volunteering at the university olympiad. I’m not sure if I will participate or not. But it is really interesting for me to do such things for my university.”*  (Interview 11, female student from university 5 enrolled in social sciences) |
