# Supplementary material for: Student behavior at university: The development and validation of a 10-dimensional scale
Source: PLoS One. 2024 Nov 15;19(11):e0313357. doi: 10.1371/journal.pone.0313357 (PMC11567547; doi:10.1371/journal.pone.0313357)
Supplement: S2 File — (DOCX) [file pone.0313357.s002.docx]

S2 File. The student behavior questionnaire in the English and Russian languages

*Table of contents*

English version of the instrument – p. 1-6

Original version of the instrument (in the Russian language) – p. 7-14

Table 1. Items measuring student behavior at university in the English and Russian languages – p. 15-19

Screenshots of the instrument measuring student behavior at university from the web survey (in Russian) – p. 20-47

*English version of the instrument measuring student behavior at university (translated from the original Russian version presented in the second part of this file).*

**In the following questions, we ask you how often you participate in different academic and non-academic activities at university. Try to answer these questions sincerely. Remember that there are no right or wrong answers, and no one will judge you for having certain attitudes towards your studying at university. All your responses are confidential and will be used only for research purposes and in an aggregate form.**

**1. {Academic diligence} Please, indicate how often you did the following at your university during the current academic year**

|  | 1 – Never | 2 – Rarely | 3 – Sometimes | 4 – Somewhat often | 5 – Very often | 99 – Do not know/ Not applicable |
| --- | --- | --- | --- | --- | --- | --- |
| In class, tried to take detailed notes so as not to forget important information |  |  |  |  |  |  |
| Tried to constantly and carefully listen to an instructor in class so as not to miss anything important |  |  |  |  |  |  |
| If an instructor asked students a question, tried to find the correct answer for yourself, even if you did not have to answer it out loud |  |  |  |  |  |  |
| When listen to a lecture or re-read notes, tried to deeper understand the new course material through relating it to what you already know |  |  |  |  |  |  |
| Dedicated almost all of your time to study on weekdays and weekends |  |  |  |  |  |  |
| Coming home after classes, tried to review the covered course material and comprehend it |  |  |  |  |  |  |
| Coming home after classes, looked for additional sources of information (for example, lectures on YouTube) to better understand the course material |  |  |  |  |  |  |
| Completed all homework thoroughly throughout the study week |  |  |  |  |  |  |
| Did extra work other than homework to ensure you have a good understanding of the course material |  |  |  |  |  |  |
| Tried to find more information about the topic being studied in the class |  |  |  |  |  |  |
| Even if the course was not interesting to you, still forced yourself to learn the material and complete assignments |  |  |  |  |  |  |
| Paid attention to how your learning is organized and how to make it most effective (planned the time and chose specific places for doing homework) |  |  |  |  |  |  |
| Tried to learn and review course material in a timely manner, and not put everything off until the last minute |  |  |  |  |  |  |
| Invested a lot of time and effort to complete an assignment that you could not done the first time |  |  |  |  |  |  |
| Skipped classes |  |  |  |  |  |  |
| Came to class unprepared |  |  |  |  |  |  |
| Submitted for assessment papers that were purchased or copied |  |  |  |  |  |  |
| Copied homework from your classmates which should be done independently |  |  |  |  |  |  |
| Did things other than your studies during class |  |  |  |  |  |  |
| Were busy with our own thoughts and did not listen to an instructor during class |  |  |  |  |  |  |

**2. {Active learning} Please, indicate how often you did the following at your university during the current academic year**

|  | 1 – Never | 2 – Rarely | 3 – Sometimes | 4 – Somewhat often | 5 – Very often | 99 – Do not know/ Not applicable |
| --- | --- | --- | --- | --- | --- | --- |
| Asked questions to an instructor during classes |  |  |  |  |  |  |
| Participated in discussions or debates on course materials in class |  |  |  |  |  |  |
| Made a class presentation |  |  |  |  |  |  |
| Answered instructor’s questions or show work on an assignment at the board |  |  |  |  |  |  |
| Speaking in front of an audience during class with spontaneous speech without prior preparation |  |  |  |  |  |  |
| Provided arguments to support your point of view in an oral or written discussion |  |  |  |  |  |  |
| Evaluated the work of other students and gave them “feedback” during class |  |  |  |  |  |  |
| Did a group assignment, participated in group work in class |  |  |  |  |  |  |
| Asked an instructor / teacher assistant to help you when you could not understand some course material on your own |  |  |  |  |  |  |
| Discussed topics related to a course with an instructor outside of class |  |  |  |  |  |  |
| Asked questions to an instructor during classes |  |  |  |  |  |  |

**3. {Social integration} Please, indicate how often you did the following at your university during the current academic year**

|  | 1 – Never | 2 – Rarely | 3 – Sometimes | 4 – Somewhat often | 5 – Very often | 99 – Do not know/ Not applicable |
| --- | --- | --- | --- | --- | --- | --- |
| Chatted with classmates in a group chat |  |  |  |  |  |  |
| Communicated with classmates during breaks between classes |  |  |  |  |  |  |
| Spent time free from studies and university activities with your classmates |  |  |  |  |  |  |
| Attended student parties |  |  |  |  |  |  |
| Learned course material or completed homework with classmates |  |  |  |  |  |  |
| Helped classmates understand complicated course material, explained how to complete an assignment that they could not do by their own |  |  |  |  |  |  |
| Asked classmates for help on academic issues |  |  |  |  |  |  |
| Asked classmates for help on personal issues not related to study |  |  |  |  |  |  |
| Tried to meet new interesting people at an university, looked for opportunities for this |  |  |  |  |  |  |
| Tried to remember as much information as possible about your classmates (names, what they are interested in, etc.) |  |  |  |  |  |  |
| Tried to build good relationships with classmates, made effort for this |  |  |  |  |  |  |

**4. {Conformity behavior} Please, indicate how often you did the following at your university during the current academic year**

|  | 1 – Never | 2 – Rarely | 3 – Sometimes | 4 – Somewhat often | 5 – Very often | 99 – Do not know/ Not applicable |
| --- | --- | --- | --- | --- | --- | --- |
| Attended absolutely all classes, even if they were useless for you |  |  |  |  |  |  |
| Tried to behave in class in such a way as to show the teacher a good side |  |  |  |  |  |  |
| Did some work that your instructor or other faculty ask you to do and that is not a part of your curriculum, even if you did not want to do it |  |  |  |  |  |  |
| Agreed with what an instructor said, even if you thought that he/she was wrong |  |  |  |  |  |  |
| In class, tried to behave in a such way to demonstrate that you are good in course material while it was not true |  |  |  |  |  |  |
| In conversation with classmates, mentioned a lesser amount of time than you actually spend on preparation to a test or exam |  |  |  |  |  |  |
| Put less effort into studying so as not to seem like a “nerd” to your university friends |  |  |  |  |  |  |

**5. {Extracurricular involvement} Please, indicate how often you did the following at your university during the current academic year**

|  | 1 – Never | 2 – Rarely | 3 – Sometimes | 4 – Somewhat often | 5 – Very often | 99 – Do not know/ Not applicable |
| --- | --- | --- | --- | --- | --- | --- |
| Attended student clubs organized at an university |  |  |  |  |  |  |
| Participated in a research, applied or creative project together with other university students |  |  |  |  |  |  |
| Attended university extracurricular events (concerts, public lectures, workshops, etc.) |  |  |  |  |  |  |
| Participated in meetings of student organizations |  |  |  |  |  |  |
| Were engaged in organizing of university events |  |  |  |  |  |  |
| Were engaged in volunteering as part of university initiatives |  |  |  |  |  |  |

*Original version of the instrument measuring student behavior at university in the Russian language*

**В следующих вопросах мы спросим у вас о вашей деятельности в университете, а также о том, как часто вам приходится совершать различные учебные и неучебные действия в разных ситуациях. Постарайтесь отвечать искренне на эти вопросы. Помните, что здесь нет правильных и неправильных ответов, и никто не осудит вас за определенное отношение к учебе. Кроме того, все ваши ответы являются конфиденциальными и будут использоваться только в исследовательских целях и обобщенном виде.**

**1. {Академическое усердие} Укажите, пожалуйста, как часто во время обучения в университете в этом учебном году вы делали следующее**

|  | 1 – Никогда | 2 – Крайне редко | 3 – Время от времени | 4 – Скорее часто | 5 – Очень часто | 99 – Затрудняюсь ответить/ Не применимо ко мне |
| --- | --- | --- | --- | --- | --- | --- |
| На лекциях и семинарах старались вести подробные конспекты, чтобы не забыть важную информацию |  |  |  |  |  |  |
| Прикладывали усилия, чтобы постоянно внимательно слушать преподавателя на занятиях и не пропустить что-то важное |  |  |  |  |  |  |
| Если преподаватель задавал вопрос студентам, старались найти на него правильный ответ для себя, даже если вам не нужно отвечать на него вслух |  |  |  |  |  |  |
| Старались понять весь материал и соотнести его с тем, что уже знаете, когда слушали лекцию или перечитывали конспекты |  |  |  |  |  |  |
| Посвящали учебе практически все свое время в будние и выходные дни |  |  |  |  |  |  |
| Приходя домой после учебы, пытались освежить в памяти пройденный материал и осмыслить его |  |  |  |  |  |  |
| Приходя домой после занятий, искали дополнительную литературу и источники (например, лекции в youtube) по теме занятий и изучали их, чтобы лучше разобраться в материале |  |  |  |  |  |  |
| Досконально выполняли все домашние задания в течение всей учебной недели |  |  |  |  |  |  |
| Делали дополнительную работу, за исключением домашних заданий, чтобы убедиться, что вы хорошо разбираетесь в материале |  |  |  |  |  |  |
| Пытались найти больше информации об изучаемой теме, чем это предусмотрено в рамках занятий |  |  |  |  |  |  |
| Даже если предмет не интересен, все равно заставляли себя учить материал и выполнять задания |  |  |  |  |  |  |
| Уделяли внимание тому, как организовано Ваше обучение и как сделать его наиболее эффективным: планировали учебную работу, выбирали определенные место для занятий |  |  |  |  |  |  |
| Старались своевременно изучать и повторять материал, не откладывать все на последний момент |  |  |  |  |  |  |
| Прикладывали много времени и усилий, чтобы выполнить задание, которое не получалось сделать сразу |  |  |  |  |  |  |
| Пропускали занятия без уважительной причины |  |  |  |  |  |  |
| Приходили на занятия неподготовленными |  |  |  |  |  |  |
| Сдавали преподавателю купленную или списанную работу |  |  |  |  |  |  |
| Списывали у однокурсников задание, которое нужно было выполнить самостоятельно |  |  |  |  |  |  |
| Занимались делами не по учебе во время занятия |  |  |  |  |  |  |
| Были заняты своими мыслями и не слушали преподавателя во время занятия |  |  |  |  |  |  |

**2. {Учебная активность} Укажите, пожалуйста, как часто во время обучения в университете в этом учебном году вы делали следующее**

|  | 1 – Никогда | 2 – Крайне редко | 3 – Время от времени | 4 – Скорее часто | 5 – Очень часто | 99 – Затрудняюсь ответить/ Не применимо ко мне |
| --- | --- | --- | --- | --- | --- | --- |
| Задавали вопросы преподавателю во время занятий |  |  |  |  |  |  |
| Участвовали во время занятий в обсуждении учебных вопросов / дебатах |  |  |  |  |  |  |
| Выступали с докладом или презентацией во время занятий |  |  |  |  |  |  |
| Отвечали на вопросы преподавателя или разбирали задание перед аудиторией («у доски») |  |  |  |  |  |  |
| Выступали перед аудиторией во время занятия со спонтанной речью без предварительной подготовки |  |  |  |  |  |  |
| Участвовали в разыгрывании сценок по ролям |  |  |  |  |  |  |
| Приводили аргументы для подтверждения своей точки зрения в устной или письменной дискуссии |  |  |  |  |  |  |
| Оценивали работу других студентов, давали им «обратную связь» во время занятия |  |  |  |  |  |  |
| Выполняли групповое задание, работали в команде во время занятия |  |  |  |  |  |  |
| Просили помощи преподавателя / ассистента преподавателя, если не могли разобраться в учебном вопросе самостоятельно |  |  |  |  |  |  |
| Обсуждали с преподавателем во внеучебное время вопросы, связанные с учебной дисциплиной |  |  |  |  |  |  |

**3. {Социальная интеграция} Укажите, пожалуйста, как часто во время обучения в университете в этом учебном году вы делали следующее**

|  | 1 – Никогда | 2 – Крайне редко | 3 – Время от времени | 4 – Скорее часто | 5 – Очень часто | 99 – Затрудняюсь ответить/ Не применимо ко мне |
| --- | --- | --- | --- | --- | --- | --- |
| Общались с одногруппниками / однокурсниками в общем онлайн-чате |  |  |  |  |  |  |
| Общались с одногруппниками / однокурсниками в перерыве между занятиями |  |  |  |  |  |  |
| Проводили время, свободное от учебы и университетских активностей, вместе со своими одногруппниками / однокурсниками |  |  |  |  |  |  |
| Посещали студенческие вечеринки / квартирники |  |  |  |  |  |  |
| Разбирали учебный материал или выполняли домашнее задание вместе с одногруппниками |  |  |  |  |  |  |
| Помогали одногруппникам разобраться в сложном материале, объясняли как выполнить задание, которое они не понимают |  |  |  |  |  |  |
| Просили помощь у одногруппников / однокурсников по учебным вопросам |  |  |  |  |  |  |
| Просили помощь у одногруппников / однокурсников по личным вопросам, не связанным с учебой |  |  |  |  |  |  |
| Пытались познакомиться с новыми интересными людьми в университете, искали возможности для этого |  |  |  |  |  |  |
| Пытались запомнить как можно больше сведений о своих одногруппниках / однокурсниках (имена, чем они увлекаются и т.д.) |  |  |  |  |  |  |
| Пытались установить дружеские и доверительные отношения с одногруппниками / однокурсниками, прикладывали усилия для этого |  |  |  |  |  |  |

**4. {Конформность} Укажите, пожалуйста, как часто во время обучения в университете в этом учебном году вы делали следующее**

|  | 1 – Никогда | 2 – Крайне редко | 3 – Время от времени | 4 – Скорее часто | 5 – Очень часто | 99 – Затрудняюсь ответить/ Не применимо ко мне |
| --- | --- | --- | --- | --- | --- | --- |
| Посещали абсолютно все занятия, даже если считали их бесполезными для себя |  |  |  |  |  |  |
| Пытались вести на занятиях себя так, чтобы показать себя преподавателю с хорошей стороны |  |  |  |  |  |  |
| Выполняли просьбу преподавателя или другого сотрудника университета, даже если вам не хотелось это делать и это не является частью учебной программы |  |  |  |  |  |  |
| Были вынуждены согласиться с преподавателем, даже если считали что он/она не прав(а) |  |  |  |  |  |  |
| Во время занятия своими действиями и высказываниями делали вид, что хорошо разбираетесь в учебном материале, хотя это не так |  |  |  |  |  |  |
| Общаясь с одногруппниками, преуменьшали количество времени и усилий, затраченных на подготовку к занятиям, контрольной работе или зачету / экзамену |  |  |  |  |  |  |
| Прикладывали меньше усилий к обучению, чтобы не показаться «заучкой» своим друзьям из университета |  |  |  |  |  |  |

**5. {Внеучебная активность} Укажите, пожалуйста, как часто во время обучения в университете в этом учебном году вы делали следующее**

|  | 1 – Никогда | 2 – Крайне редко | 3 – Время от времени | 4 – Скорее часто | 5 – Очень часто | 99 – Затрудняюсь ответить/ Не применимо ко мне |
| --- | --- | --- | --- | --- | --- | --- |
| Посещали клубы по интересам и кружки, организованные в университете |  |  |  |  |  |  |
| Участвовали в научно-исследовательском, прикладном или творческом проекте совместно с другими студентами университета |  |  |  |  |  |  |
| Посещали мероприятия, организованные в университете: концерты, публичные лекции и мастер-классы и т.д. |  |  |  |  |  |  |
| Посещали собрания студенческих организаций |  |  |  |  |  |  |
| Организовывали (участвовали в организации) университетского мероприятия |  |  |  |  |  |  |
| Занимались волонтерством в рамках университетских инициатив |  |  |  |  |  |  |

Table 1. Items measuring student behavior at university in the English and Russian languages

| Items in the English language | Items in the Russian language |
| --- | --- |
| **1. Academic diligence** | **1. Академическое усердие** |
| ***1.1. Interaction with course content in class*** | ***1.1. Взаимодействие с учебным материалом во время занятий*** |
| In class, tried to take detailed notes so as not to forget important information | На лекциях и семинарах старались вести подробные конспекты, чтобы не забыть важную информацию |
| Tried to constantly and carefully listen to an instructor in class so as not to miss anything important | Прикладывали усилия, чтобы постоянно внимательно слушать преподавателя на занятиях и не пропустить что-то важное |
| If an instructor asked students a question, tried to find the correct answer for yourself, even if you did not have to answer it out loud | Если преподаватель задавал вопрос студентам, старались найти на него правильный ответ для себя, даже если вам не нужно отвечать на него вслух |
| When listen to a lecture or re-read notes, tried to deeper understand the new course material through relating it to what you already know | Старались понять весь материал и соотнести его с тем, что уже знаете, когда слушали лекцию или перечитывали конспекты |
| ***1.2. Persistence*** | ***1.2. Упорство*** |
| Dedicated almost all of your time to study on weekdays and weekends | Посвящали учебе практически все свое время в будние и выходные дни |
| Completed all homework thoroughly throughout the study week | Досконально выполняли все домашние задания в течение всей учебной недели |
| Even if the course was not interesting to you, still forced yourself to learn the material and complete assignments | Даже если предмет не интересен, все равно заставляли себя учить материал и выполнять задания |
| Paid attention to how your learning is organized and how to make it most effective (planned the time and chose specific places for doing homework) | Уделяли внимание тому, как организовано ваше обучение и как сделать его наиболее эффективным: планировали учебную работу, выбирали определенные место для занятий |
| Tried to learn and review course material in a timely manner, and not put everything off until the last minute | Старались своевременно изучать и повторять материал, не откладывать все на последний момент |
| Invested a lot of time and effort to complete an assignment that you could not done the first time | Прикладывали много времени и усилий, чтобы выполнить задание, которое не получалось сделать сразу |
| ***1.3. Self-learning*** | ***1.3. Самообучение*** |
| Coming home after classes, tried to review the covered course material and comprehend it | Приходя домой после учебы, пытались освежить в памяти пройденный материал и осмыслить его |
| Coming home after classes, looked for additional sources of information (for example, lectures on YouTube) to better understand the course material | Приходя домой после занятий, искали дополнительную литературу и источники (например, лекции в youtube) по теме занятий и изучали их, чтобы лучше разобр |
| Did extra work other than homework to ensure you have a good understanding of the course material | Делали дополнительную работу, за исключением домашних заданий, чтобы убедиться, что вы хорошо разбираетесь в материале |
| Tried to find more information about the topic being studied in the class | Пытались найти больше информации об изучаемой теме, чем это предусмотрено в рамках занятий |
| ***1.4. Irresponsible learning behavior*** | ***1.4. Нарушение учебной дисциплины*** |
| Skipped classes | Пропускали занятия без уважительной причины |
| Came to class unprepared | Приходили на занятия неподготовленными |
| Submitted for assessment papers that were purchased or copied | Сдавали преподавателю купленную или списанную работу |
| Copied homework from your classmates which should be done independently | Списывали у однокурсников задание, которое нужно было выполнить самостоятельно |
| Did things other than your studies during class | Занимались делами не по учебе во время занятия |
| Were busy with our own thoughts and did not listen to an instructor during class | Были заняты своими мыслями и не слушали преподавателя во время занятия |
| **2. Active learning** | **2. Учебная активность** |
| Asked questions to an instructor during classes | Задавали вопросы преподавателю во время занятий |
| Participated in discussions or debates on course materials in class | Участвовали во время занятий в обсуждении учебных вопросов / дебатах |
| Made a class presentation | Выступали с докладом или презентацией во время занятий |
| Answered instructor’s questions or show work on an assignment at the board | Отвечали на вопросы преподавателя или разбирали задание перед аудиторией («у доски») |
| Speaking in front of an audience during class with spontaneous speech without prior preparation | Выступали перед аудиторией во время занятия со спонтанной речью без предварительной подготовки |
| Provided arguments to support your point of view in an oral or written discussion | Приводили аргументы для подтверждения своей точки зрения в устной или письменной дискуссии |
| Evaluated the work of other students and gave them “feedback” during class | Оценивали работу других студентов, давали им «обратную связь» во время занятия |
| Did a group assignment, participated in group work in class | Выполняли групповое задание, работали в команде во время занятия |
| Asked an instructor / teacher assistant to help you when you could not understand some course material on your own | Просили помощи преподавателя / ассистента преподавателя, если не могли разобраться в учебном вопросе самостоятельно |
| Discussed topics related to a course with an instructor outside of class | Обсуждали с преподавателем во внеучебное время вопросы, связанные с учебной дисциплиной |
| **3. Social integration** | **3. Социальная интеграция** |
| ***3.1. Student friendship*** | ***3.1. Студенческая дружба*** |
| Spent time free from studies and university activities with your classmates | Проводили время, свободное от учебы и университетских активностей, вместе со своими одногруппниками / однокурсниками |
| Attended student parties | Посещали студенческие вечеринки / квартирники |
| Asked classmates for help on personal issues not related to study | Просили помощь у одногруппников / однокурсников по личным вопросам, не связанным с учебой |
| Tried to meet new interesting people at an university, looked for opportunities for this | Пытались познакомиться с новыми интересными людьми в университете, искали возможности для этого |
| Tried to remember as much information as possible about your classmates (names, what they are interested in, etc.) | Пытались запомнить как можно больше сведений о своих одногруппниках / однокурсниках (имена, чем они увлекаются и т.д.) |
| Tried to build good relationships with classmates, made effort for this | Пытались установить дружеские и доверительные отношения с одногруппниками / однокурсниками, прикладывали усилия для этого |
| ***3.2. Study collaboration*** | ***3.2. Совместное обучение*** |
| Chatted with classmates in a group chat | Общались с одногруппниками / однокурсниками в общем онлайн-чате |
| Communicated with classmates during breaks between classes | Общались с одногруппниками / однокурсниками в перерыве между занятиями |
| Learned course material or completed homework with classmates | Разбирали учебный материал или выполняли домашнее задание вместе с одногруппниками |
| Helped classmates understand complicated course material, explained how to complete an assignment that they could not do by their own | Помогали одногруппникам разобраться в сложном материале, объясняли как выполнить задание, которое они не понимают |
| Asked classmates for help on academic issues | Просили помощь у одногруппников / однокурсников по учебным вопросам |
| **4. Conformity behavior** | **4.** **Конформное поведение** |
| ***4.1. Obedience*** | ***4.1. Послушание*** |
| Attended absolutely all classes, even if they were useless for you | Посещали абсолютно все занятия, даже если считали их бесполезными для себя |
| Tried to behave in class in such a way as to show the teacher a good side | Пытались вести на занятиях себя так, чтобы показать себя преподавателю с хорошей стороны |
| Did some work that your instructor or other faculty ask you to do and that is not a part of your curriculum, even if you did not want to do it | Выполняли просьбу преподавателя или другого сотрудника университета, даже если вам не хотелось это делать и это не является частью учебной программы |
| ***4.2. Creating a positive image of self*** | ***4.2. Создание положительного образа себя*** |
| Agreed with what an instructor said, even if you thought that he/she was wrong | Были вынуждены согласиться с преподавателем, даже если считали, что он/она не прав(а) |
| In class, tried to behave in a such way to demonstrate that you are good in course material while it was not true | Во время занятия своими действиями и высказываниями делали вид, что хорошо разбираетесь в учебном материале, хотя это не так |
| In conversation with classmates, mentioned a lesser amount of time than you actually spend on preparation to a test or exam | Общаясь с одногруппниками, преуменьшали количество времени и усилий, затраченных на подготовку к занятиям, контрольной работе или зачету / экзамену |
| Put less effort into studying so as not to seem like a “nerd” to your university friends | Прикладывали меньше усилий к обучению, чтобы не показаться «заучкой» своим друзьям из университета |
| **5. Extracurricular involvement** | **5. Внеучебная активность** |
| Attended student clubs organized at an university | Посещали клубы по интересам и кружки, организованные в университете |
| Participated in a research, applied or creative project together with other university students | Участвовали в научно-исследовательском, прикладном или творческом проекте совместно с другими студентами университета |
| Attended university extracurricular events (concerts, public lectures, workshops, etc.) | Посещали мероприятия, организованные в университете: концерты, публичные лекции и мастер-классы и т.д. |
| Participated in meetings of student organizations | Посещали собрания студенческих организаций |
| Were engaged in organizing of university events | Организовывали (участвовали в организации) университетского мероприятия |
| Were engaged in volunteering as part of university initiatives | Занимались волонтерством в рамках университетских инициатив |

**Screenshots of the instrument measuring student behavior at university from the web survey (in Russian)**

**
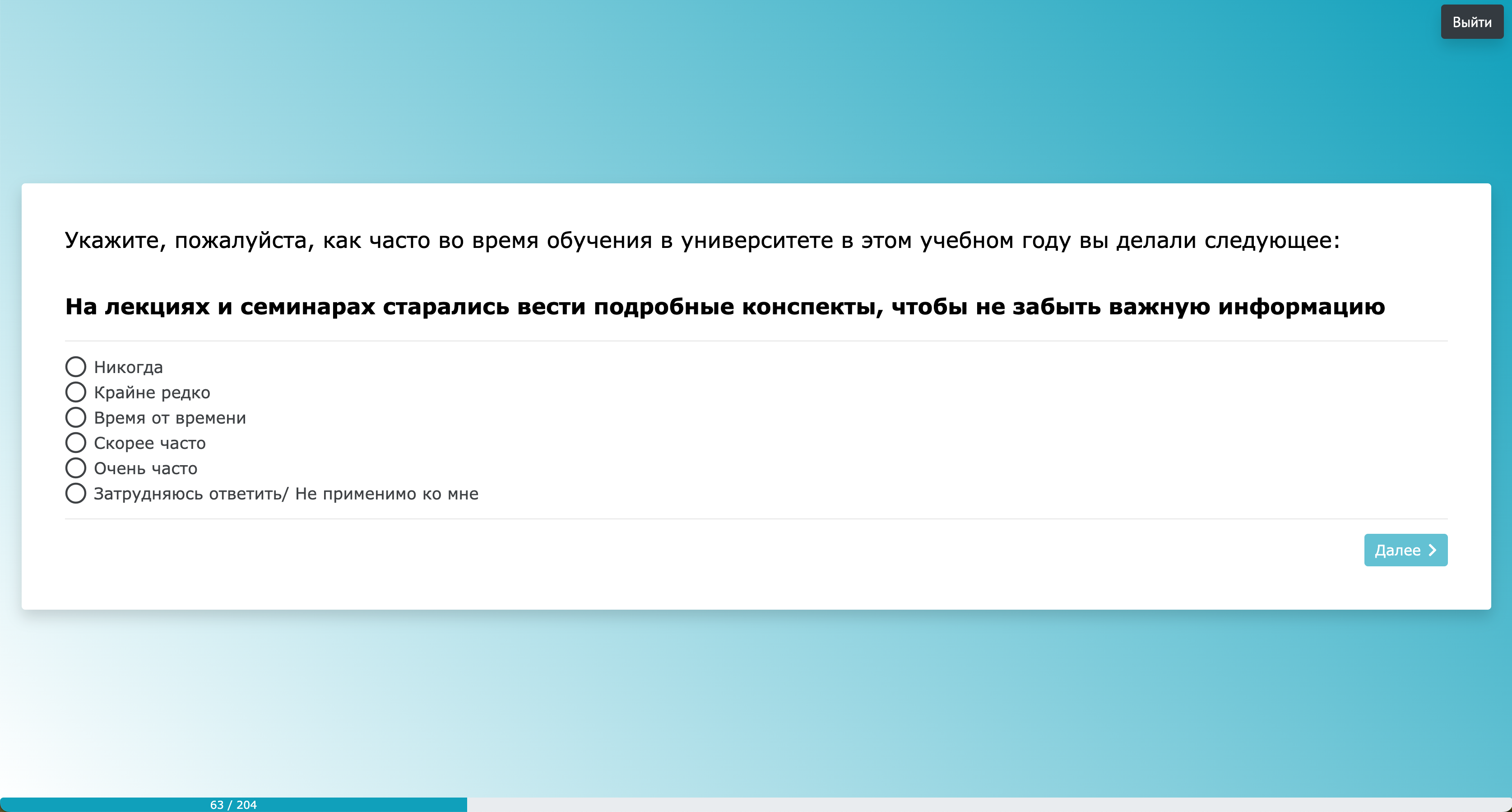
**

**
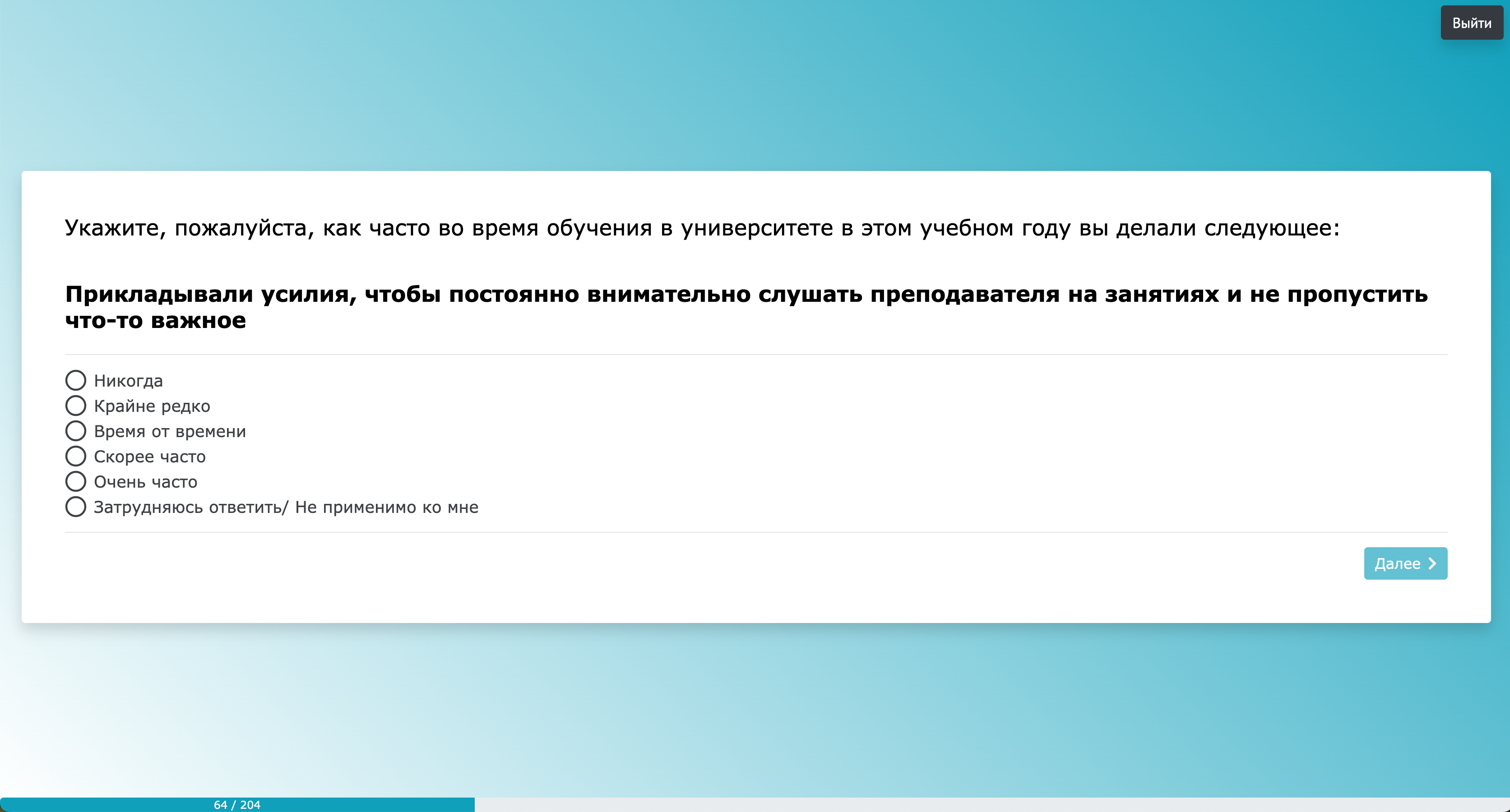
**

**
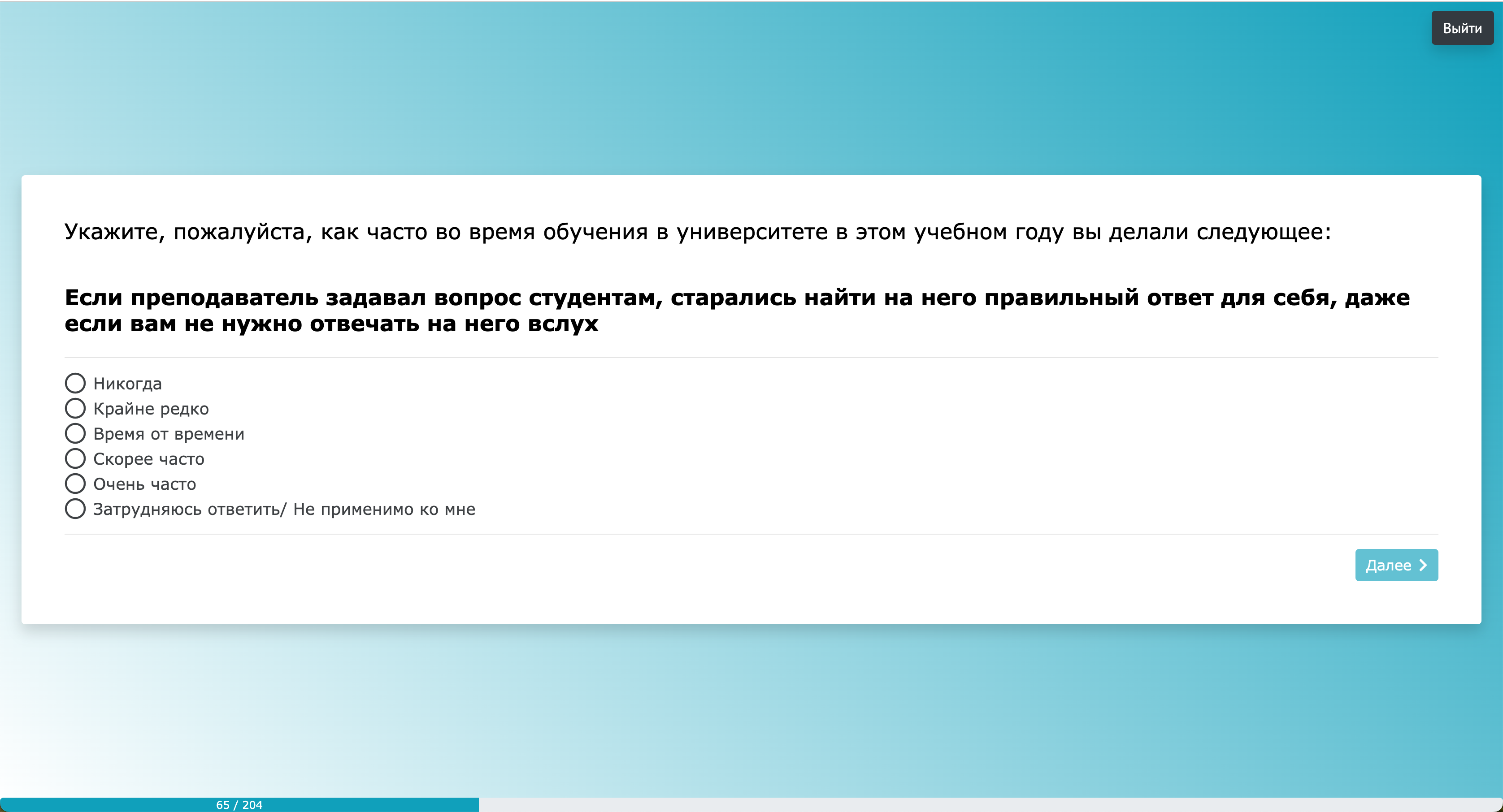
**

**
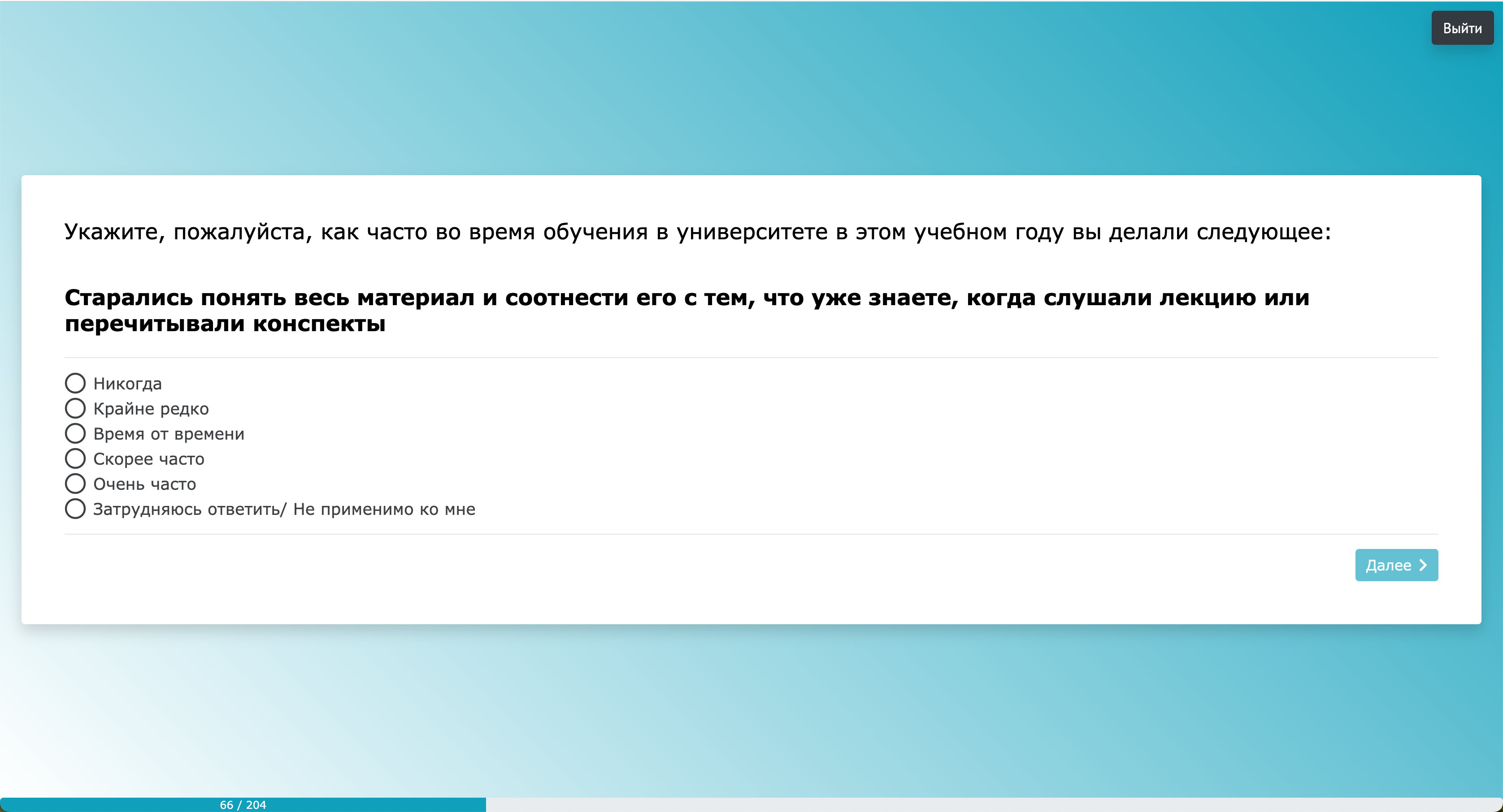
**

**
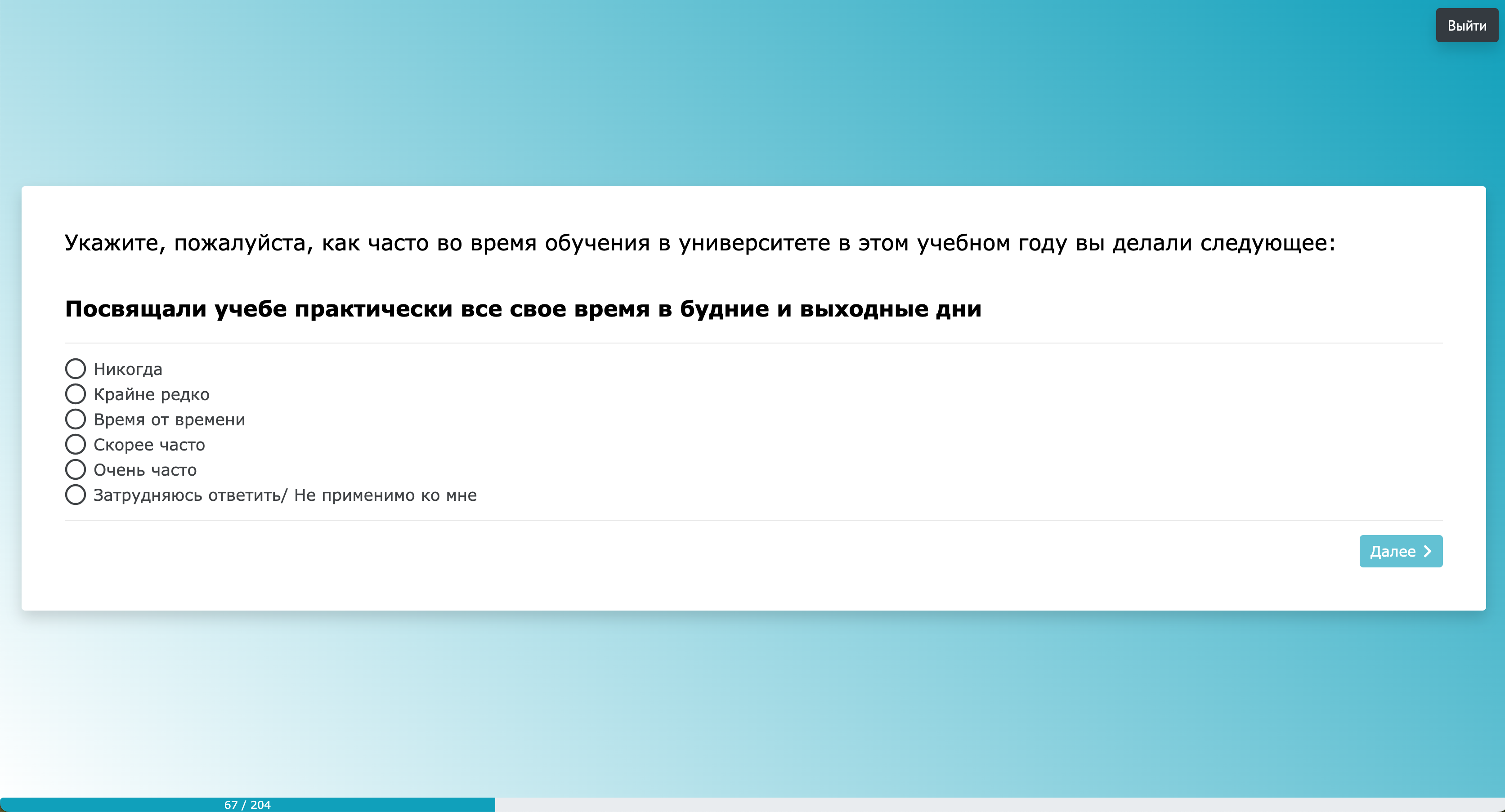
**

**
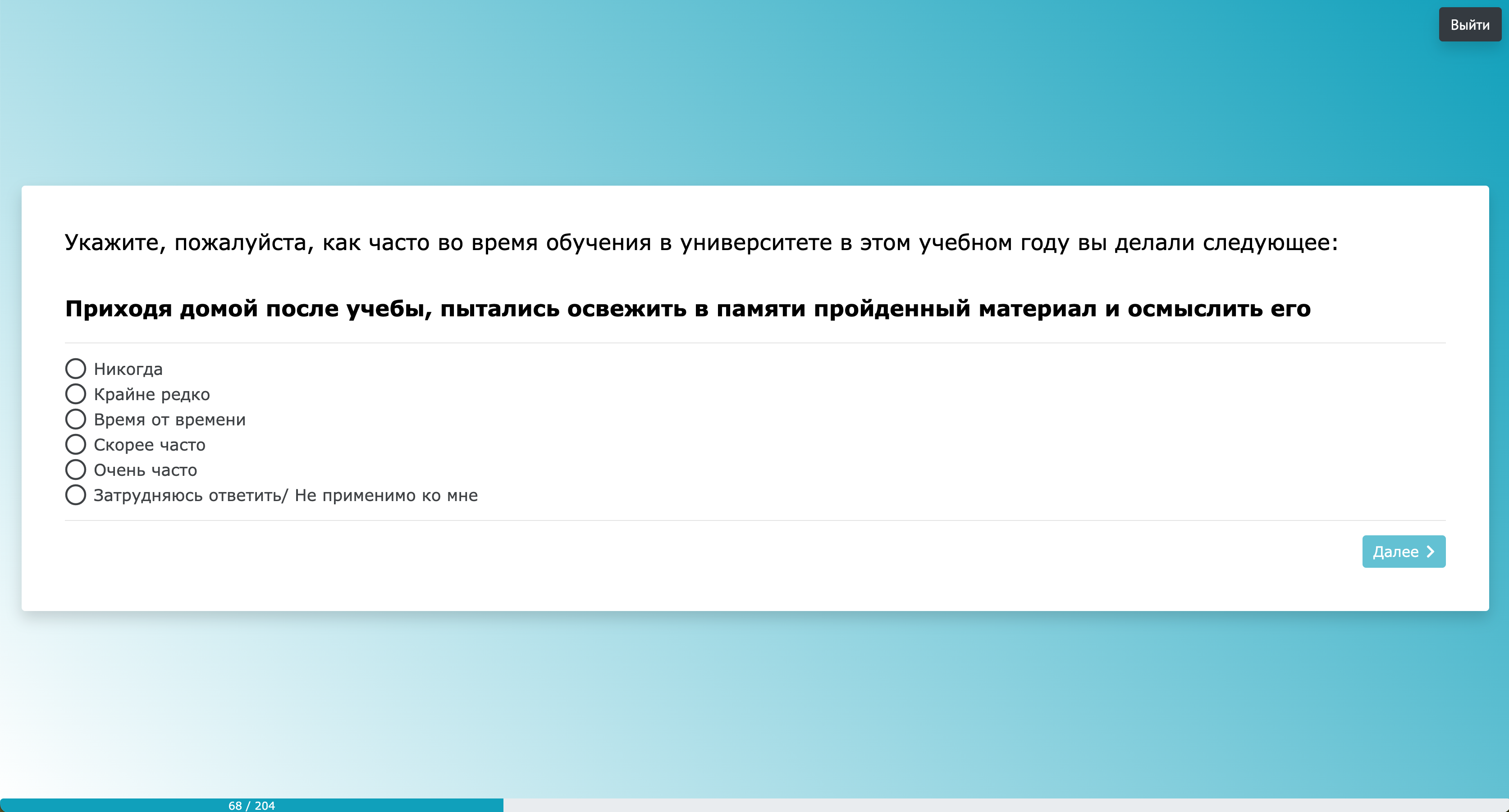
**

**
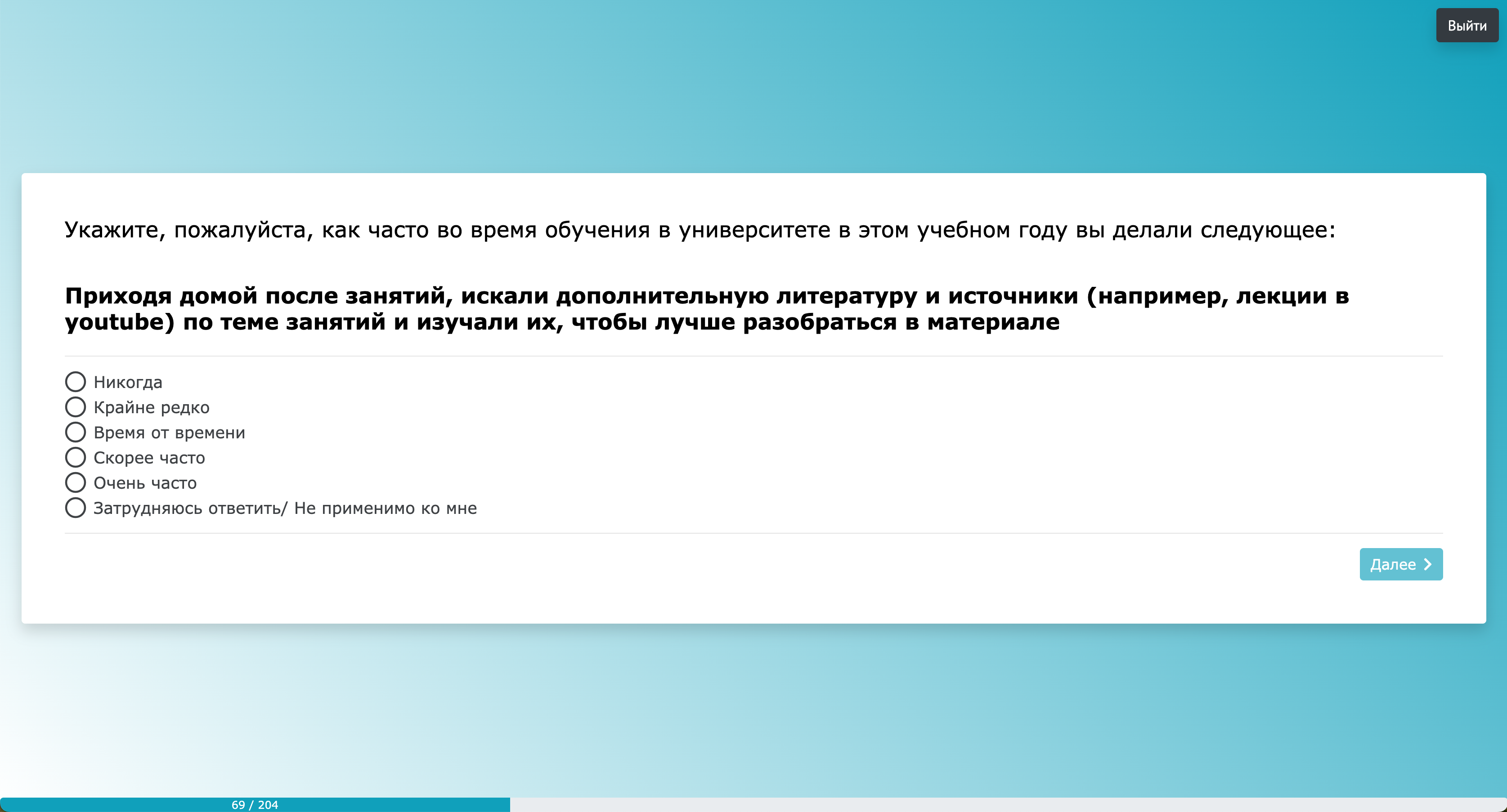
**

**
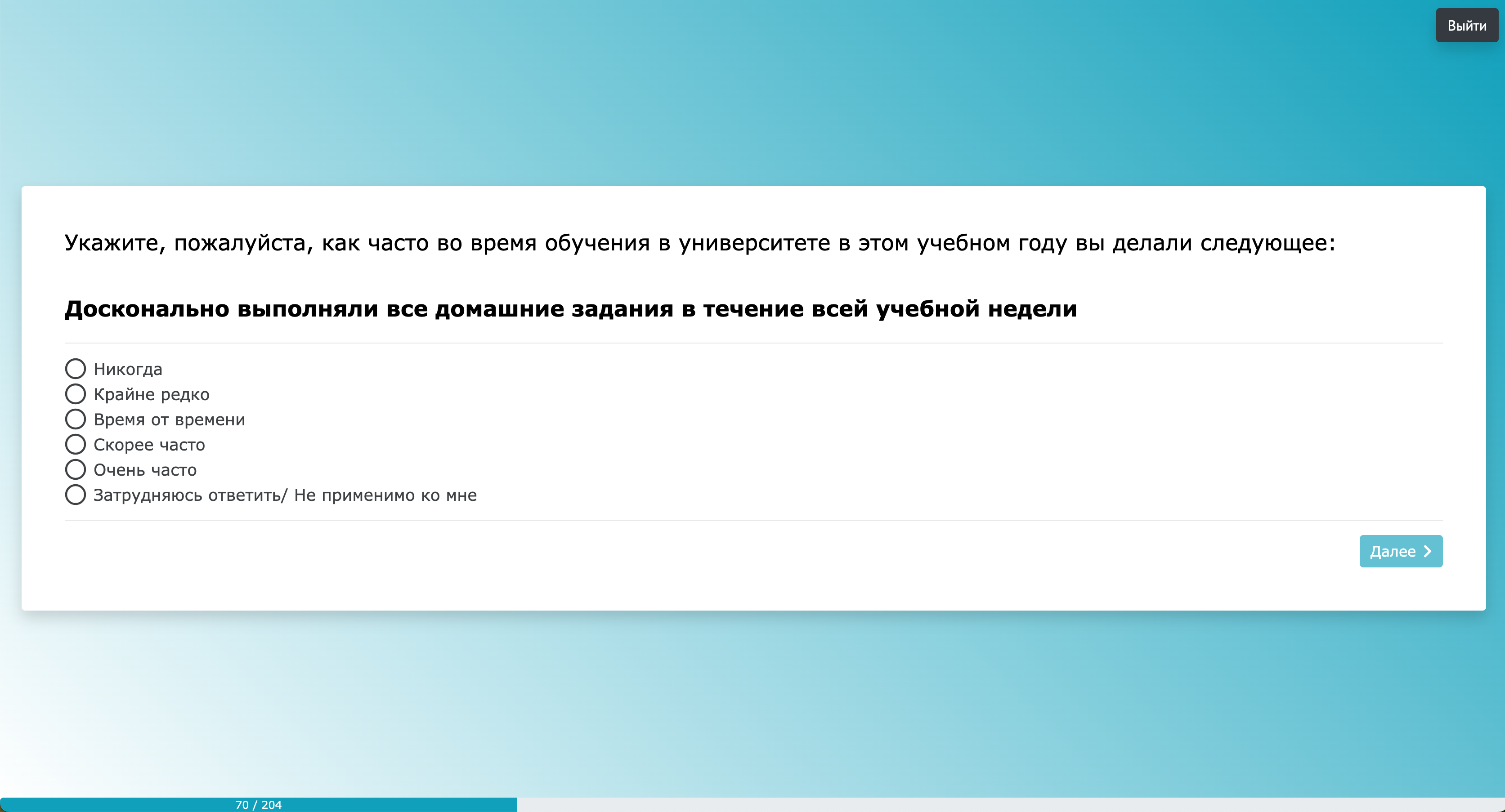
**

**
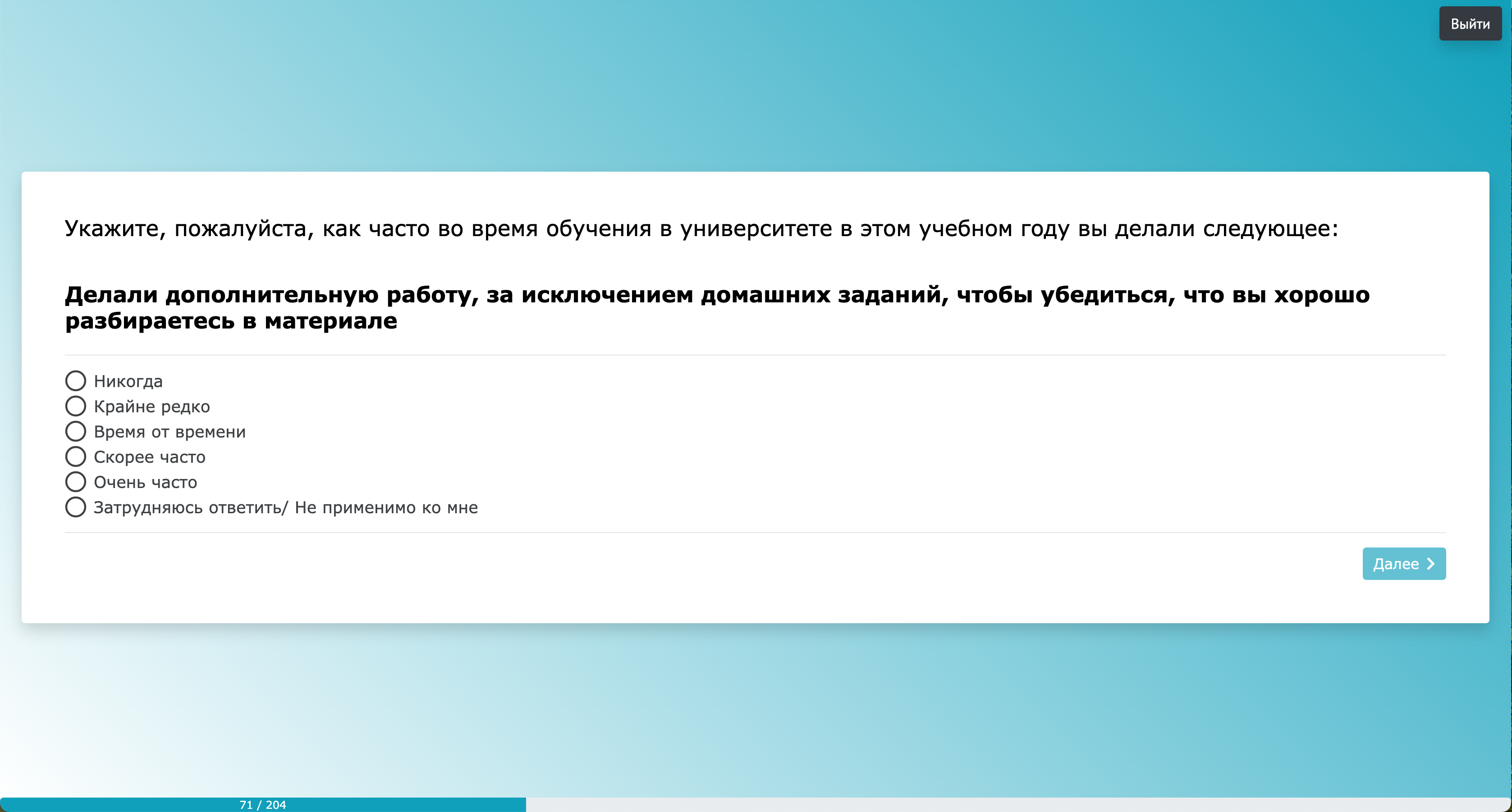
**

**
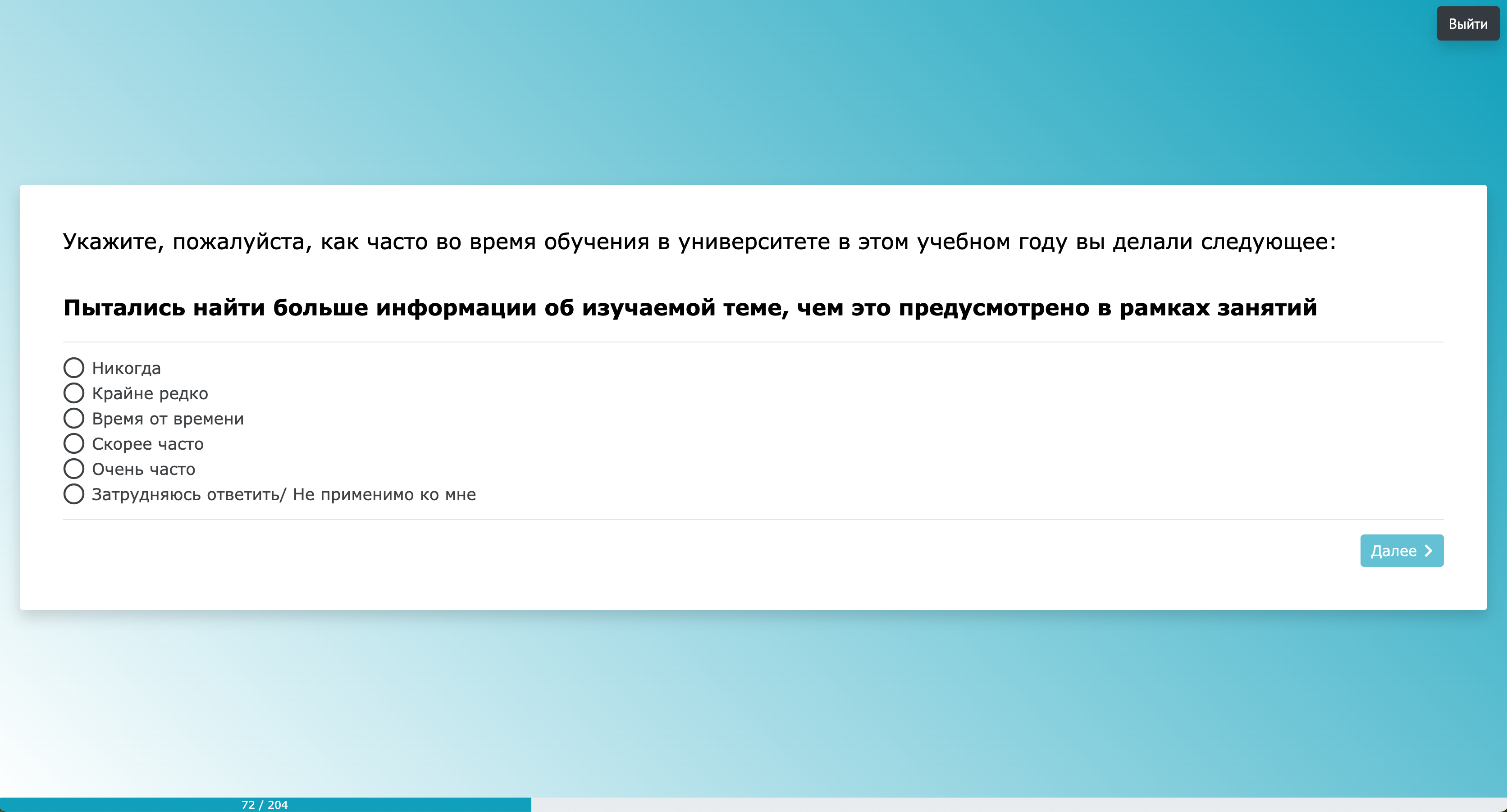
**

**
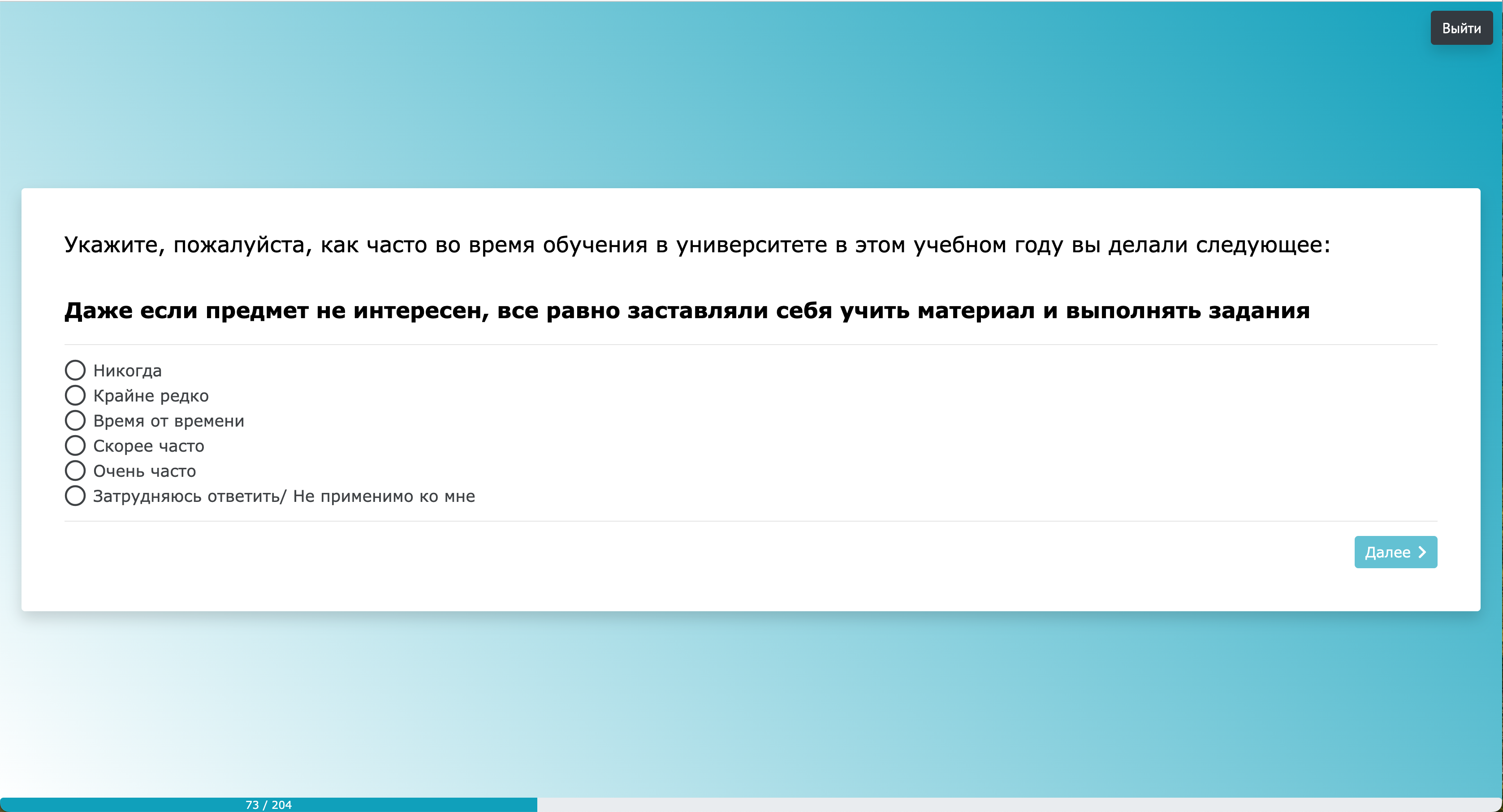
**

**
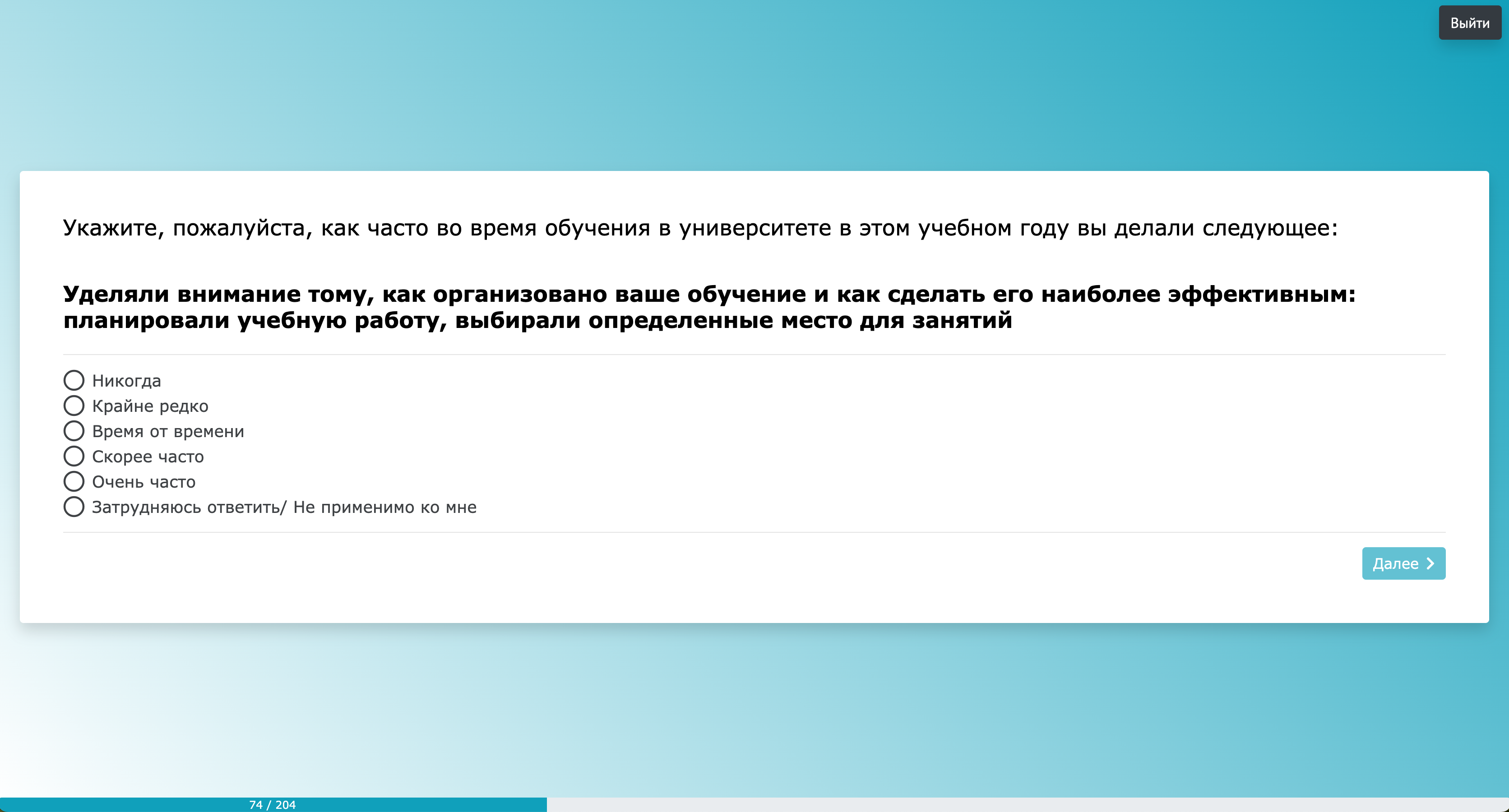
**

**
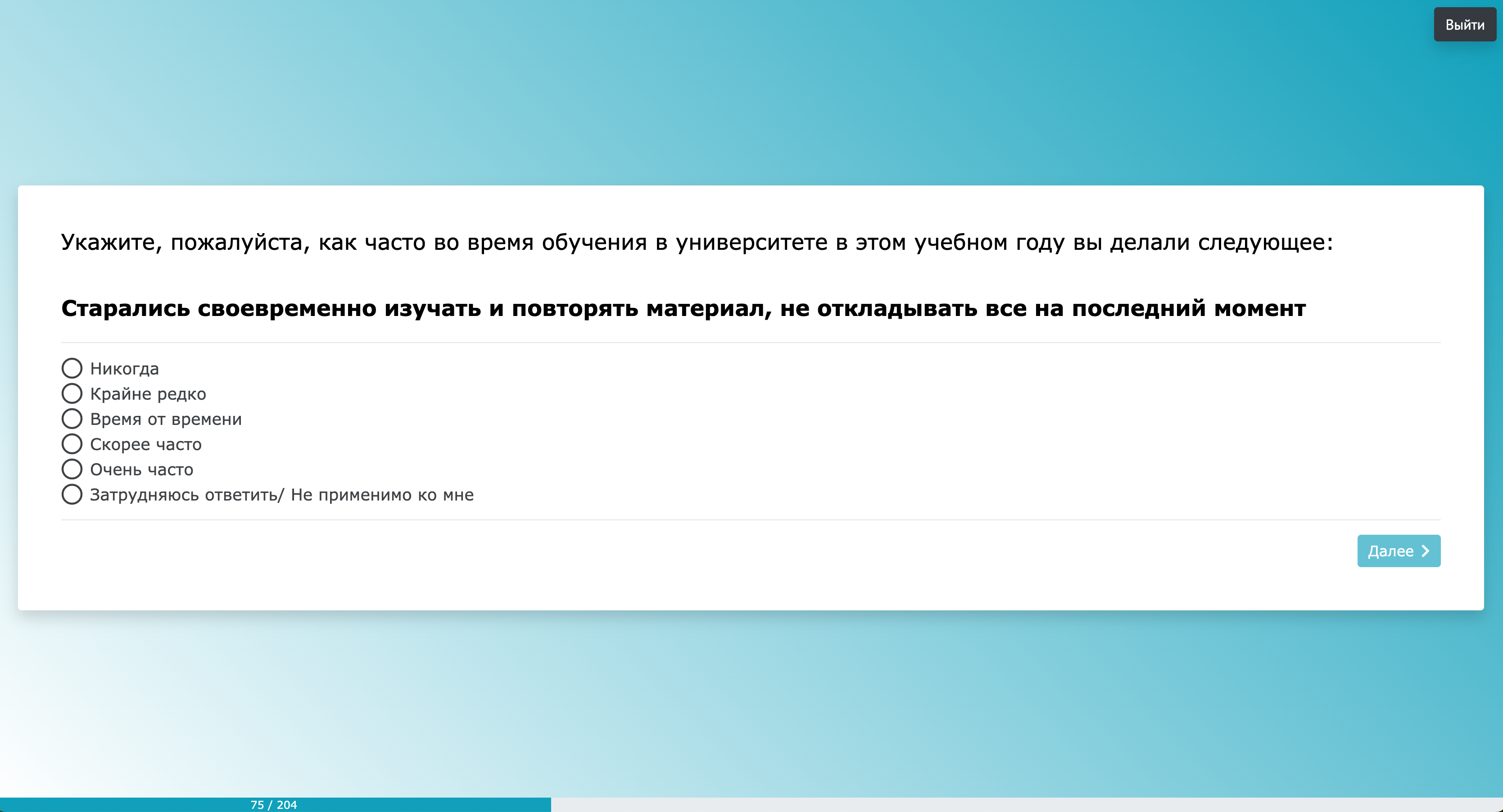
**

**
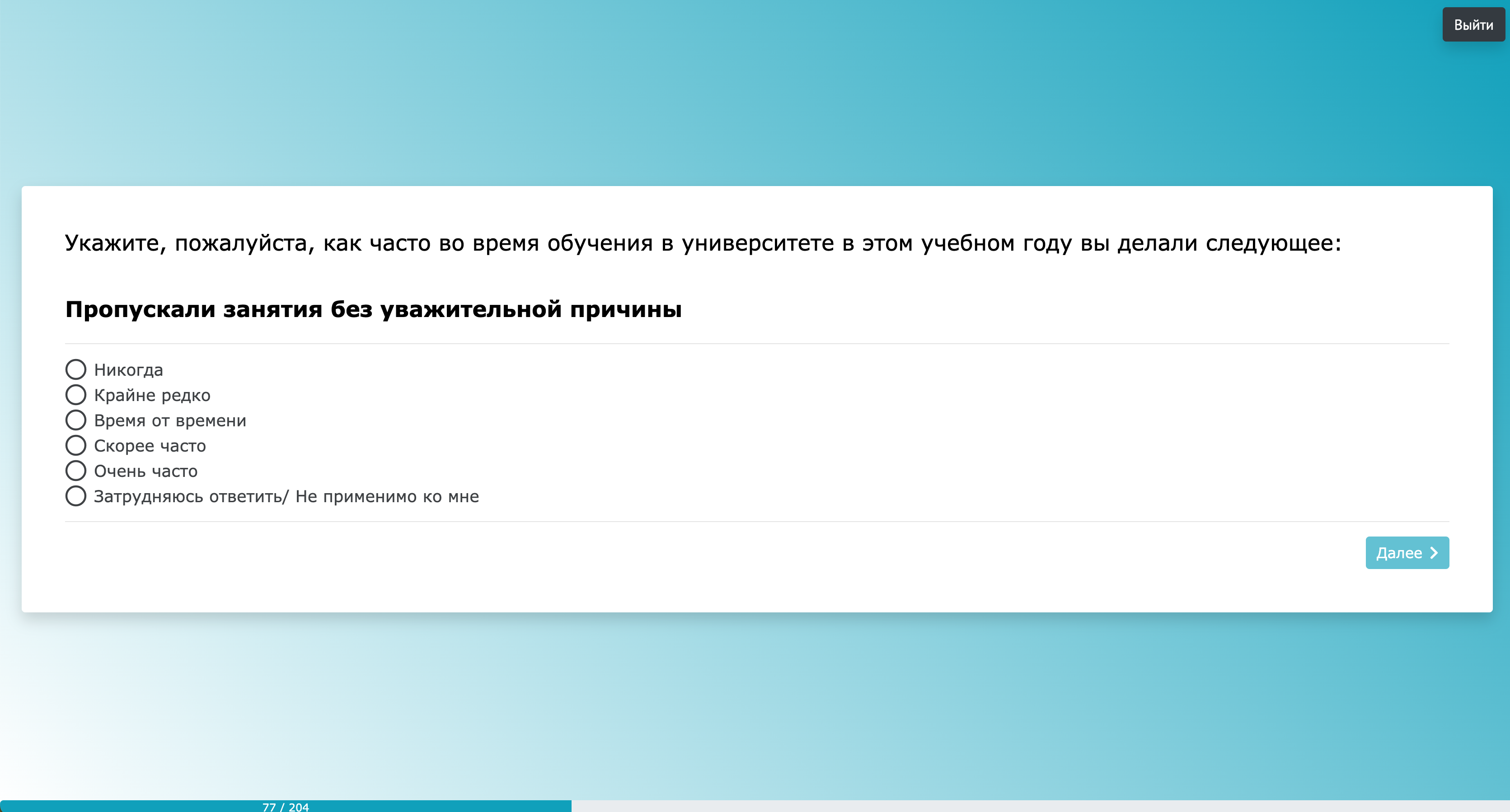
**

**
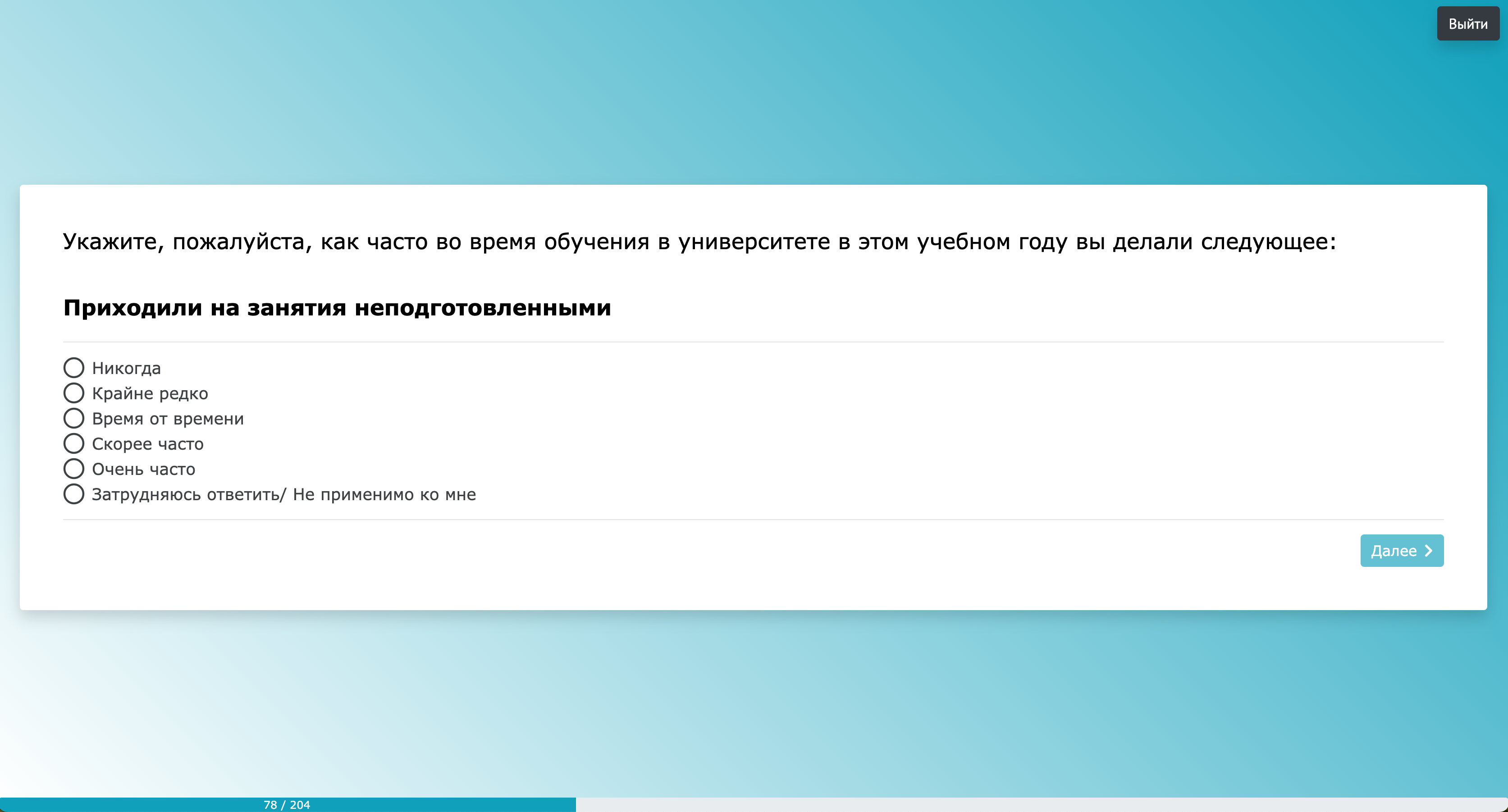
**

**
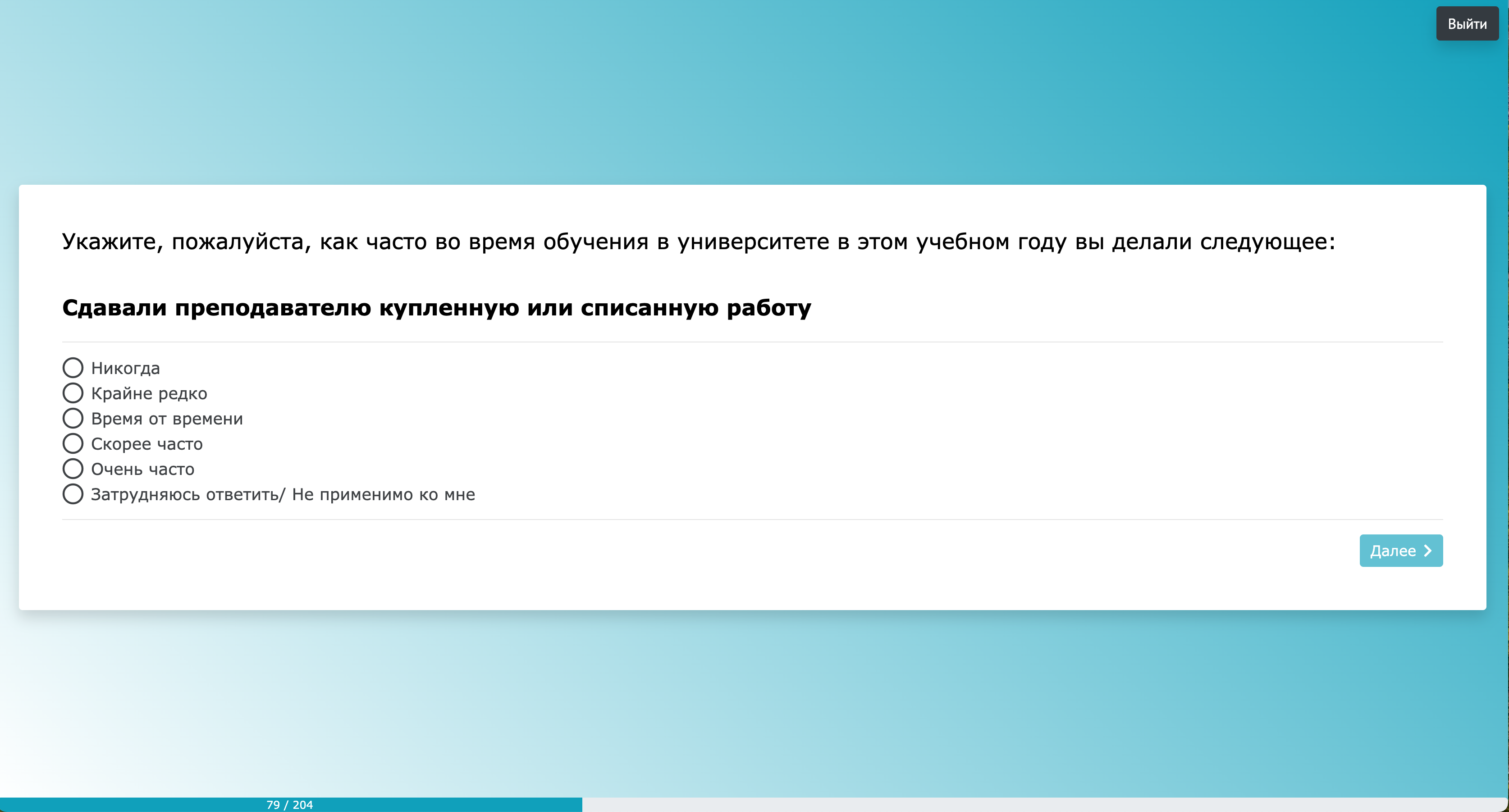
**

**
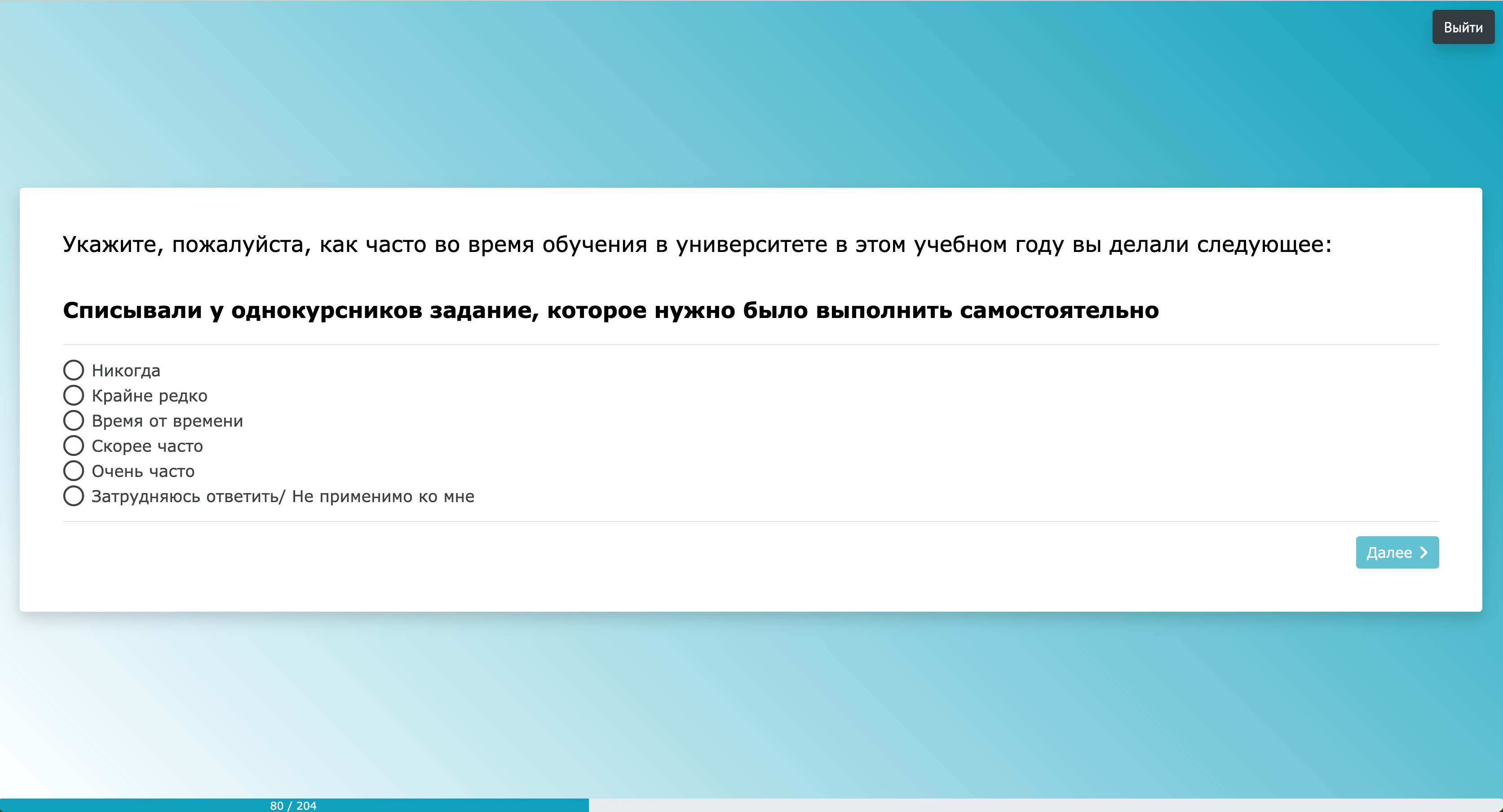
**

**
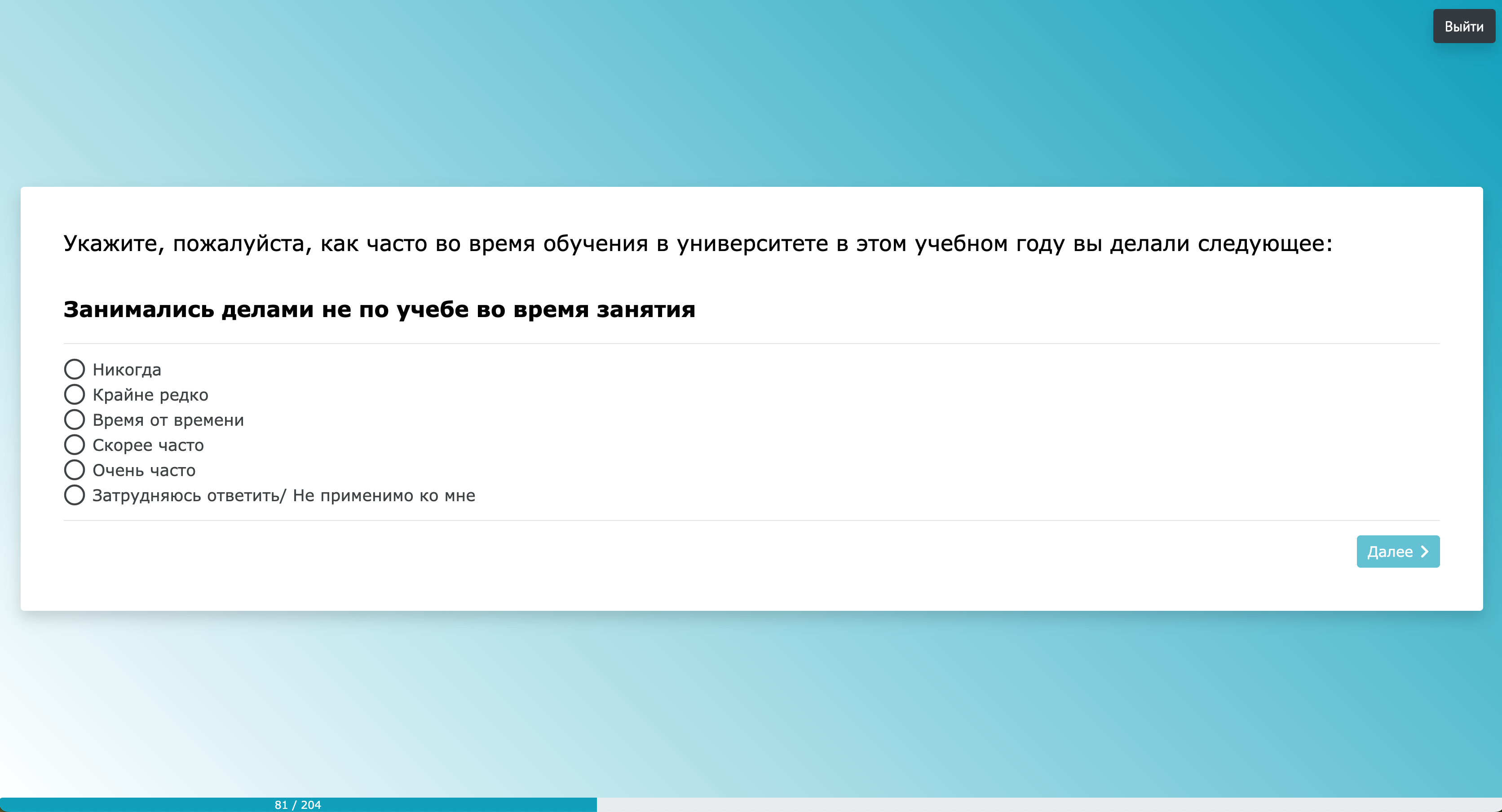
**

**
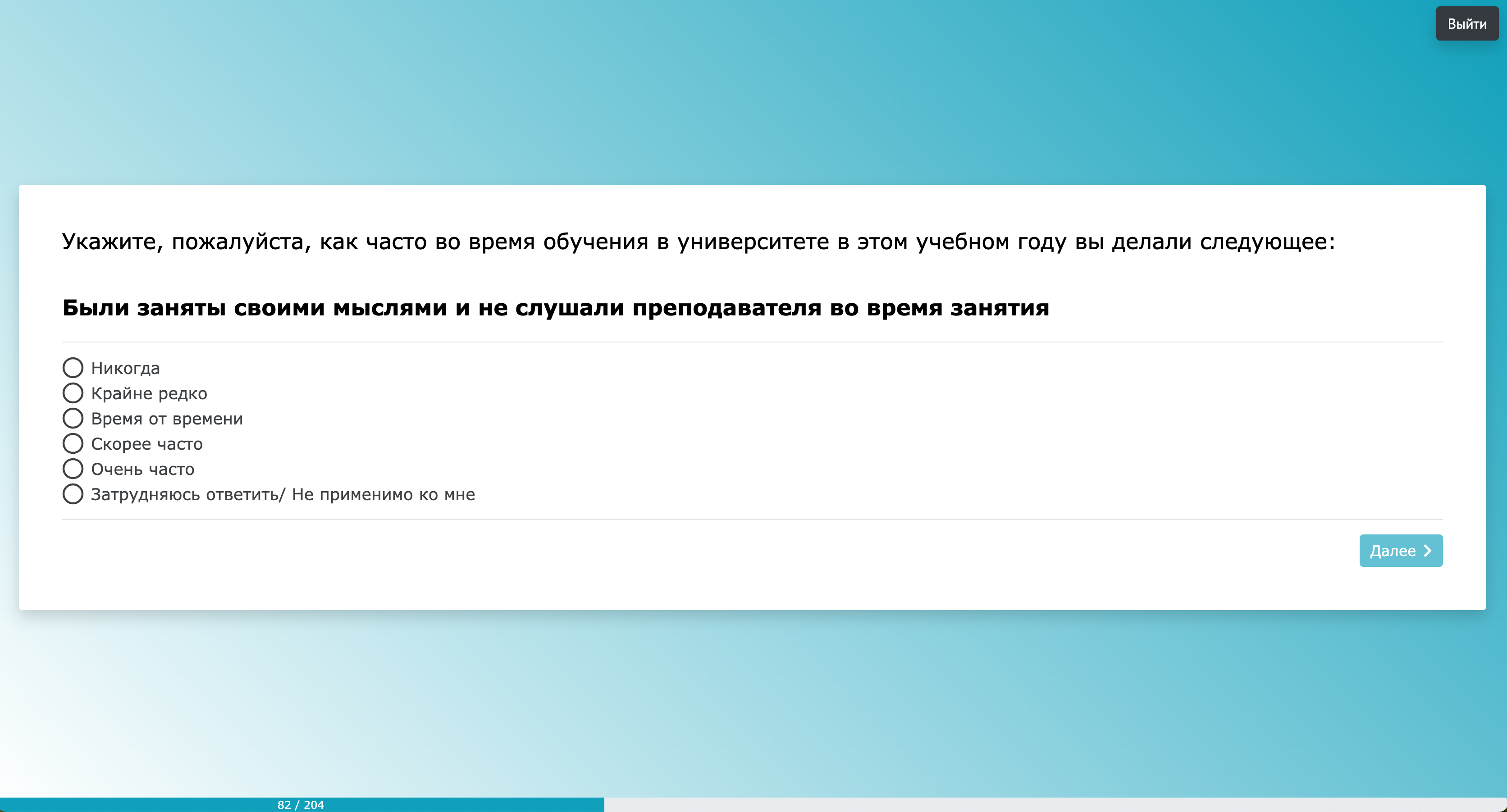
**

**
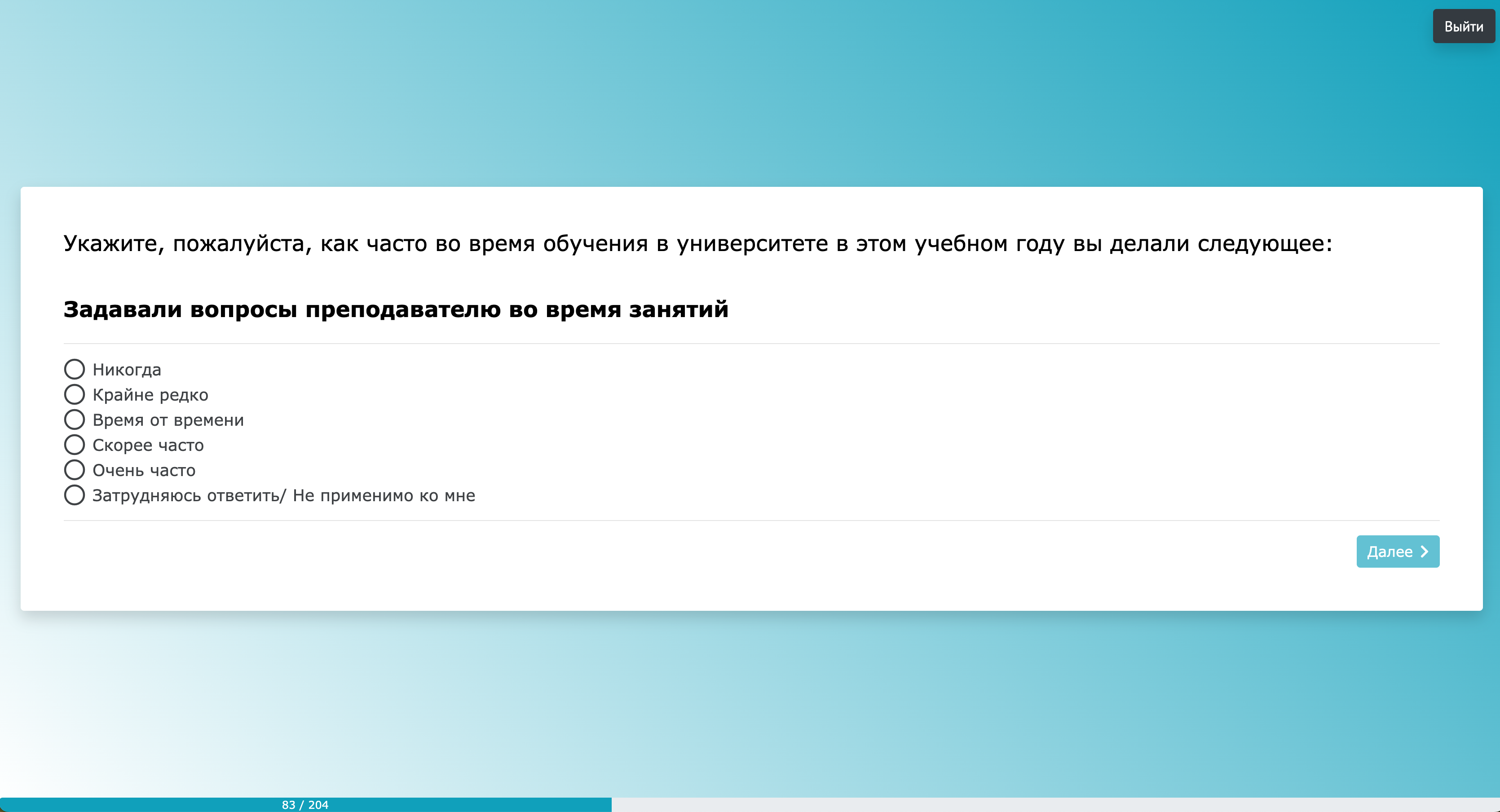
**

**
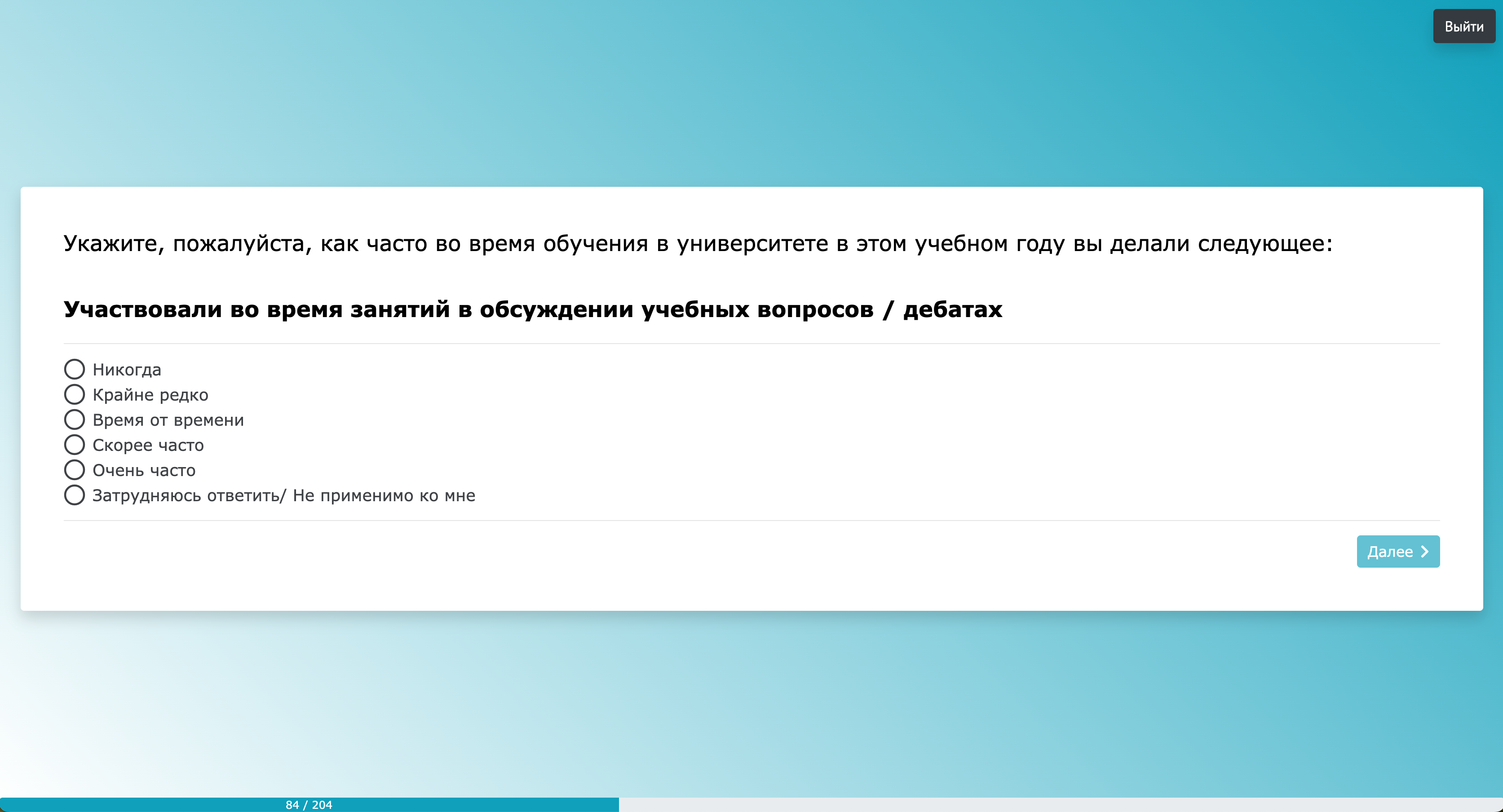
**

**
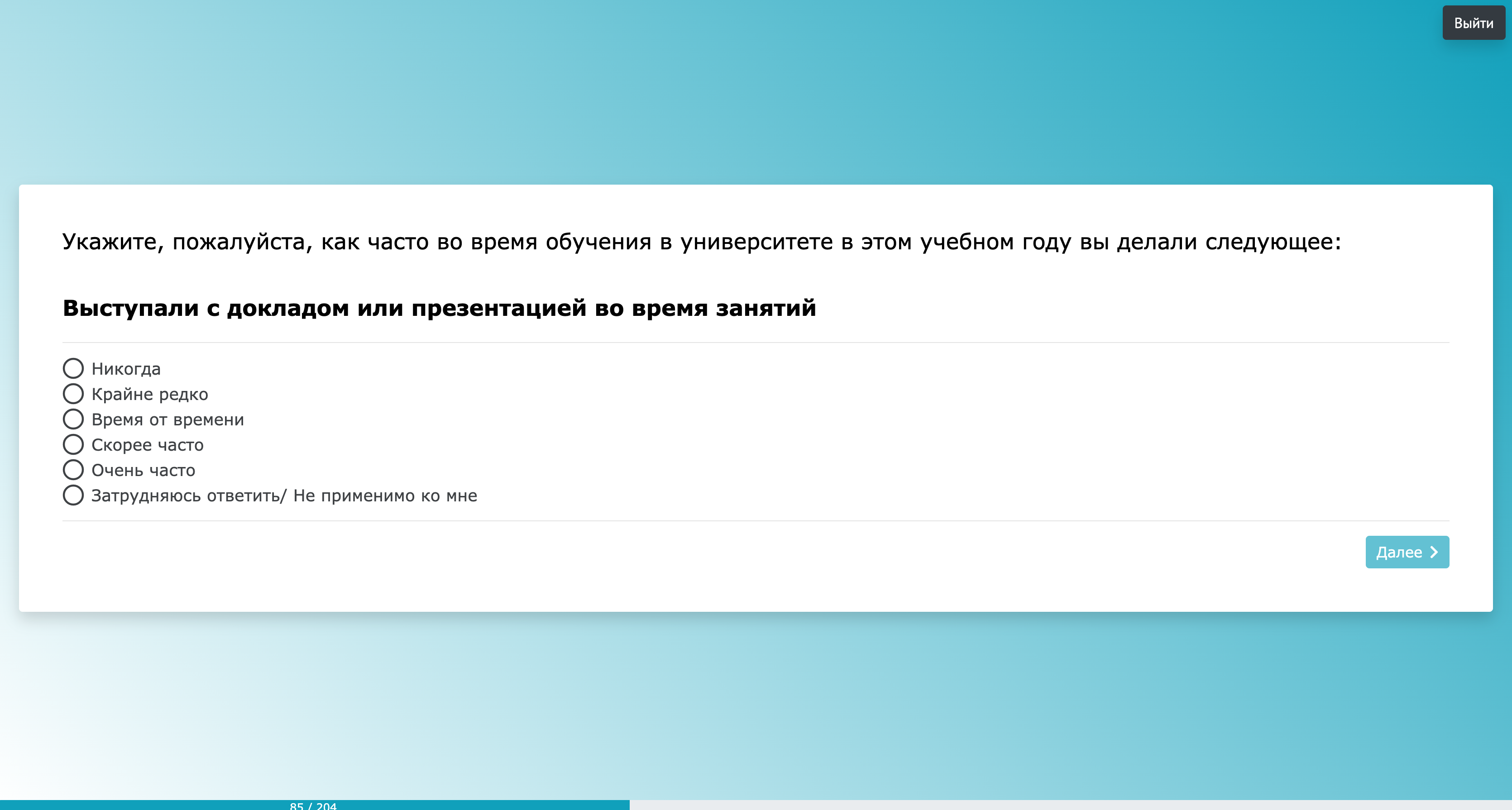
**

**
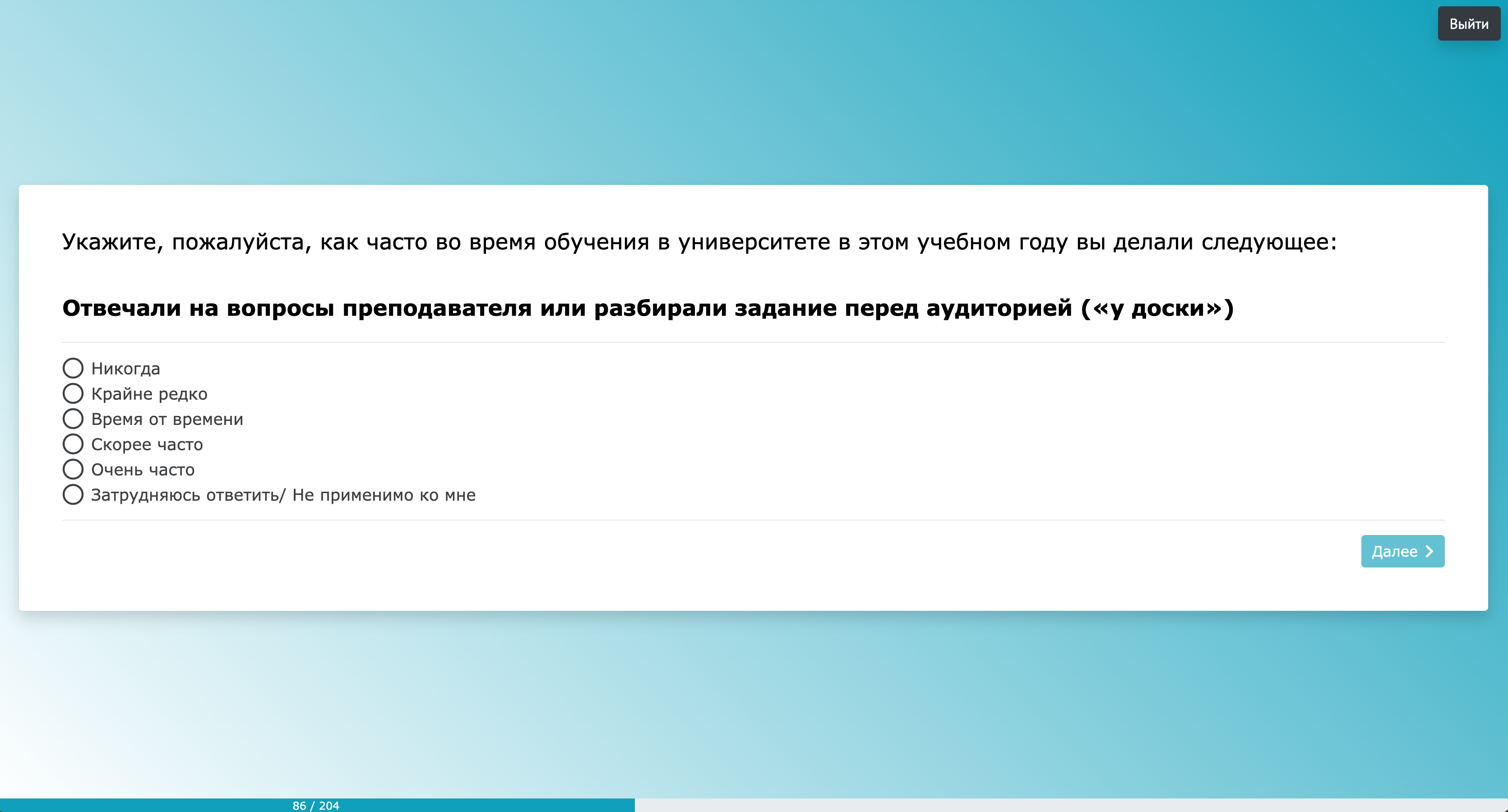
**

**
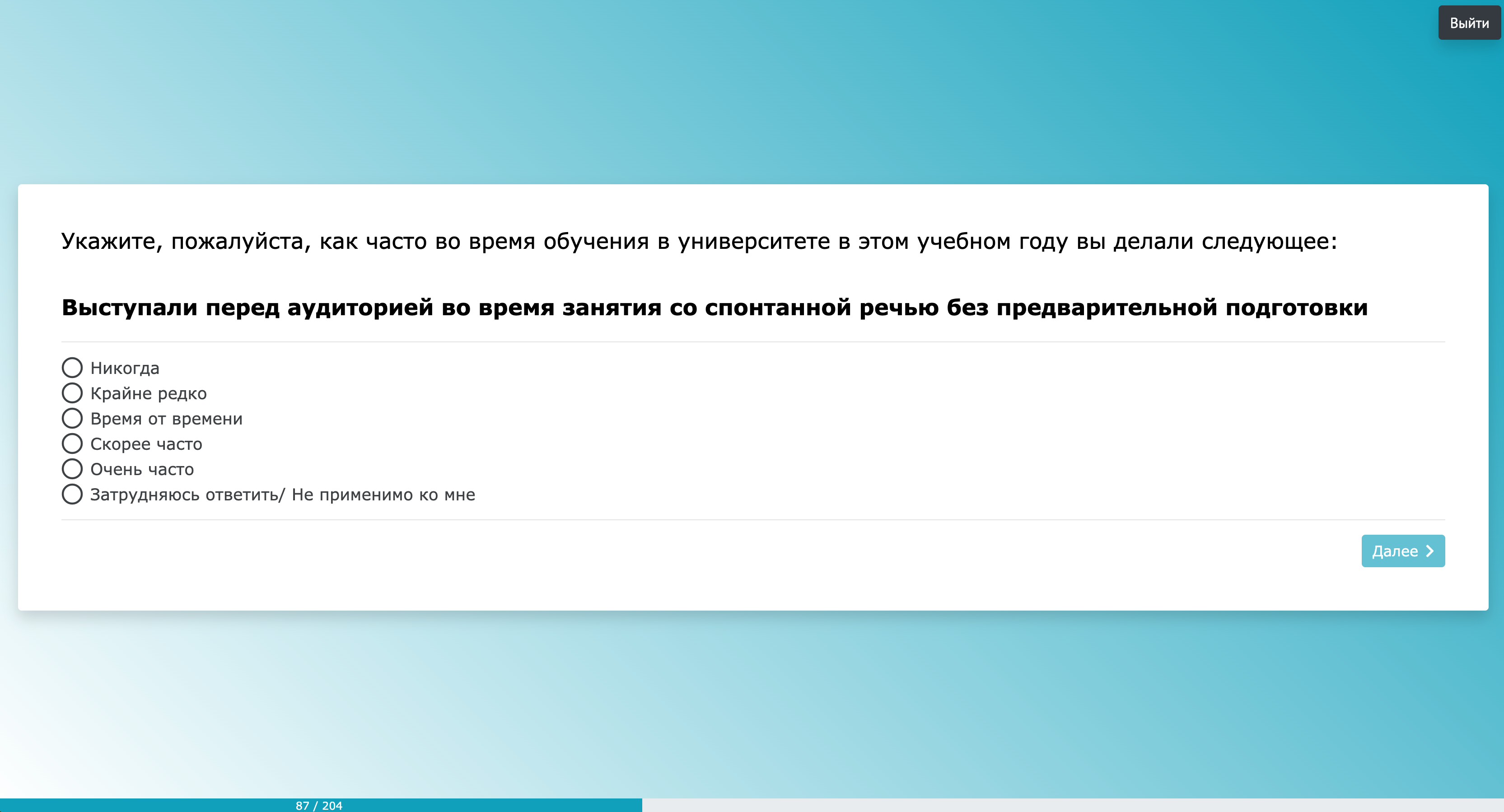
**

**
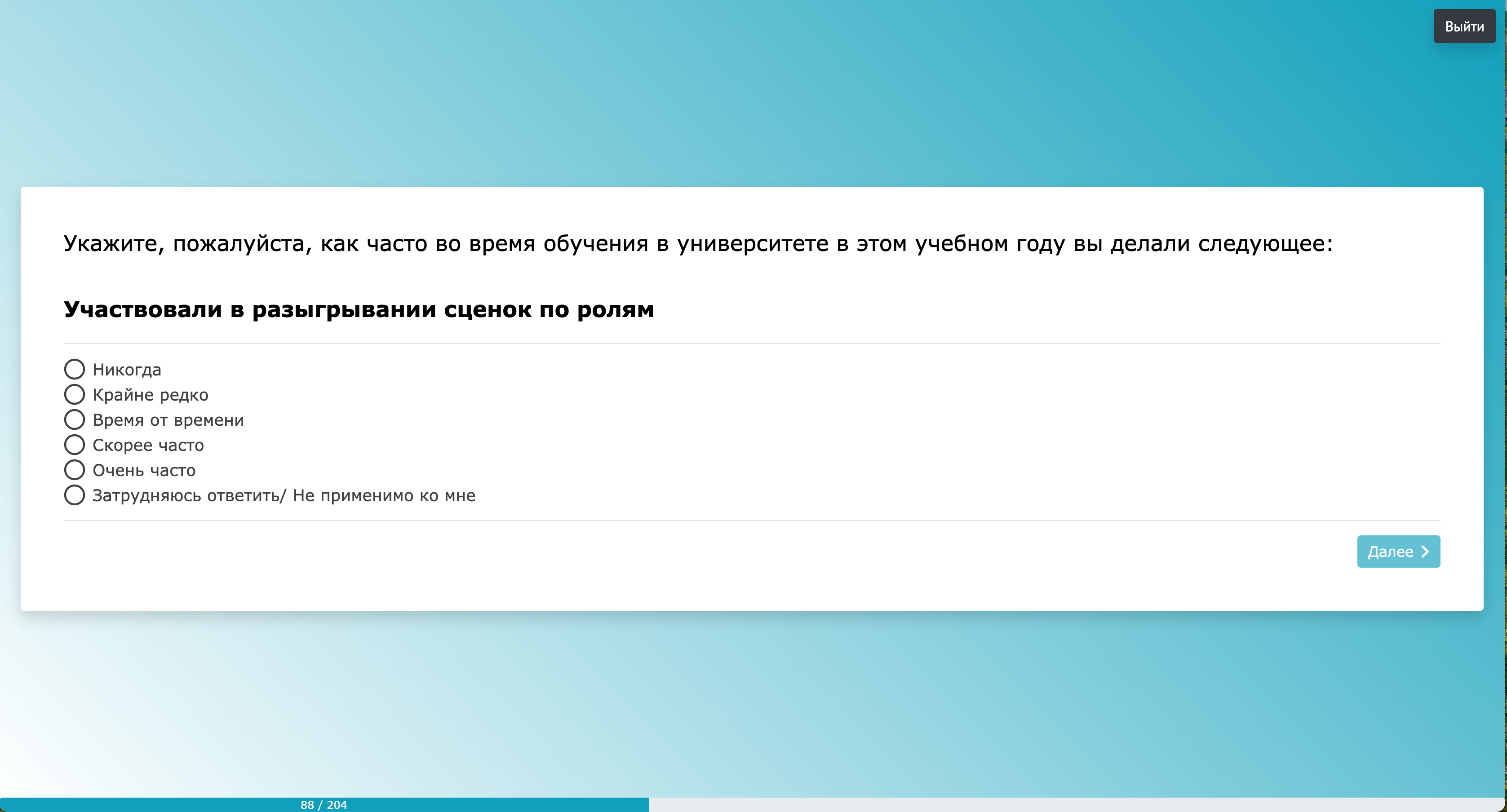
**

**
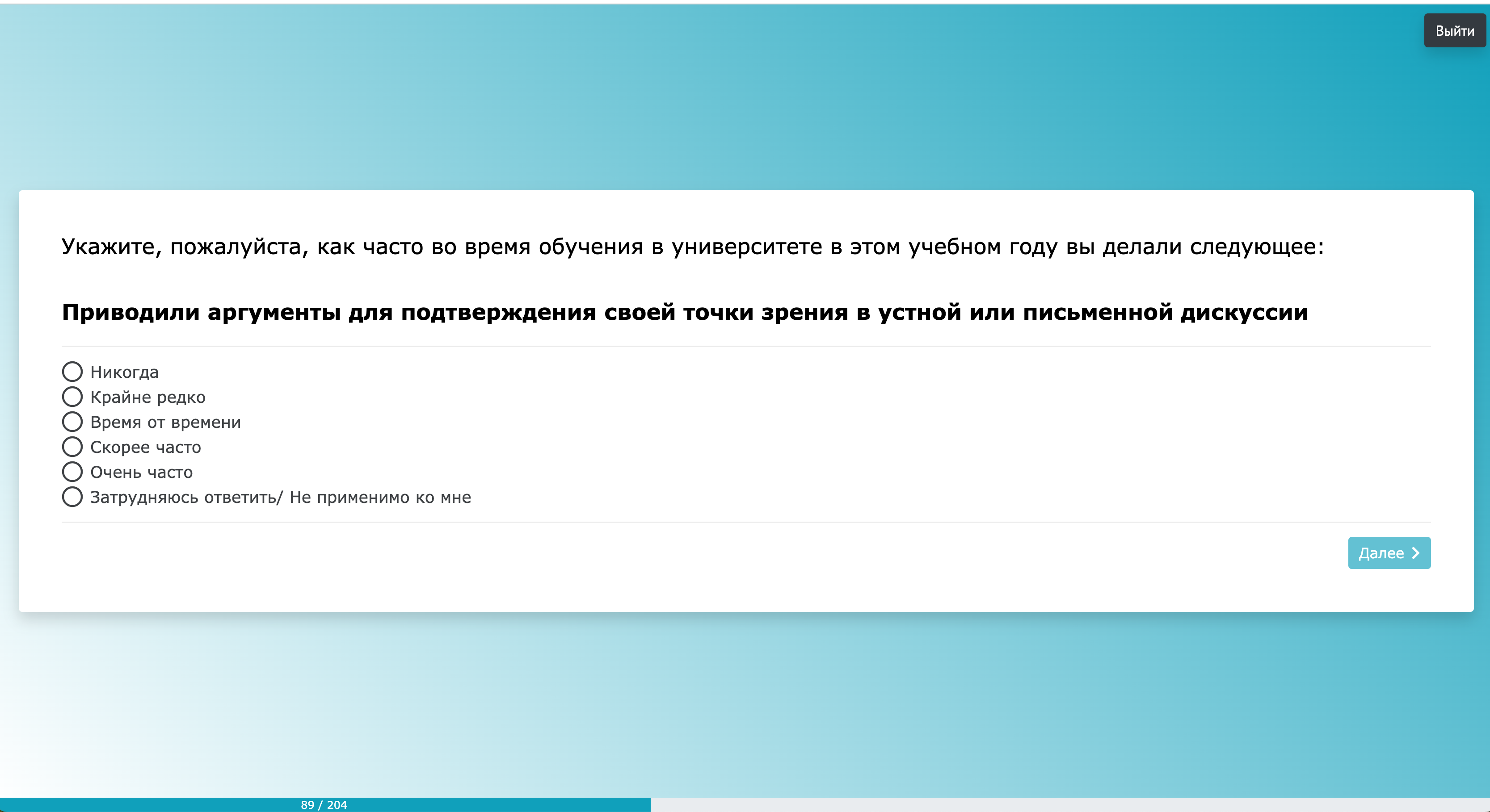
**

**
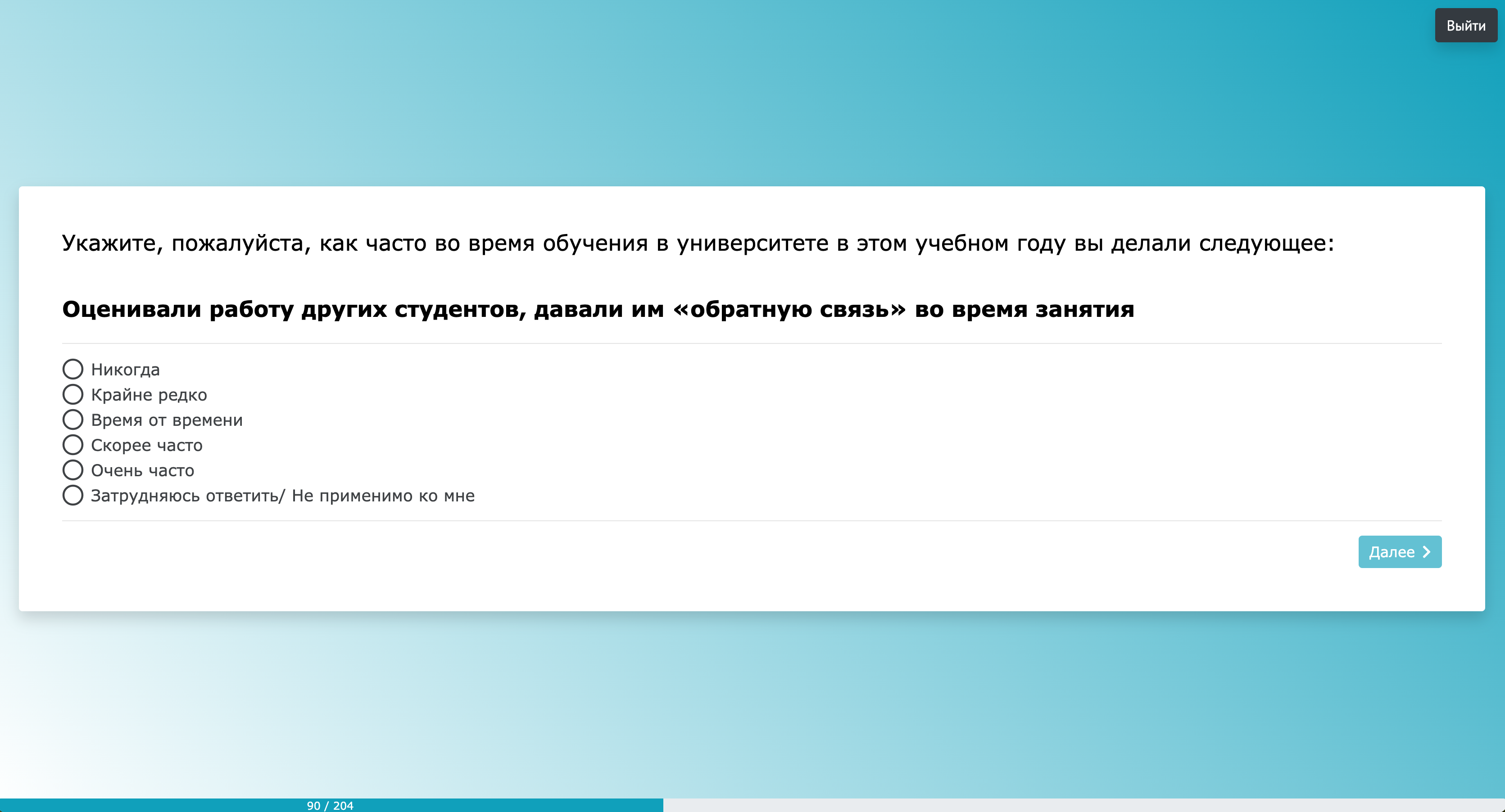
**

**
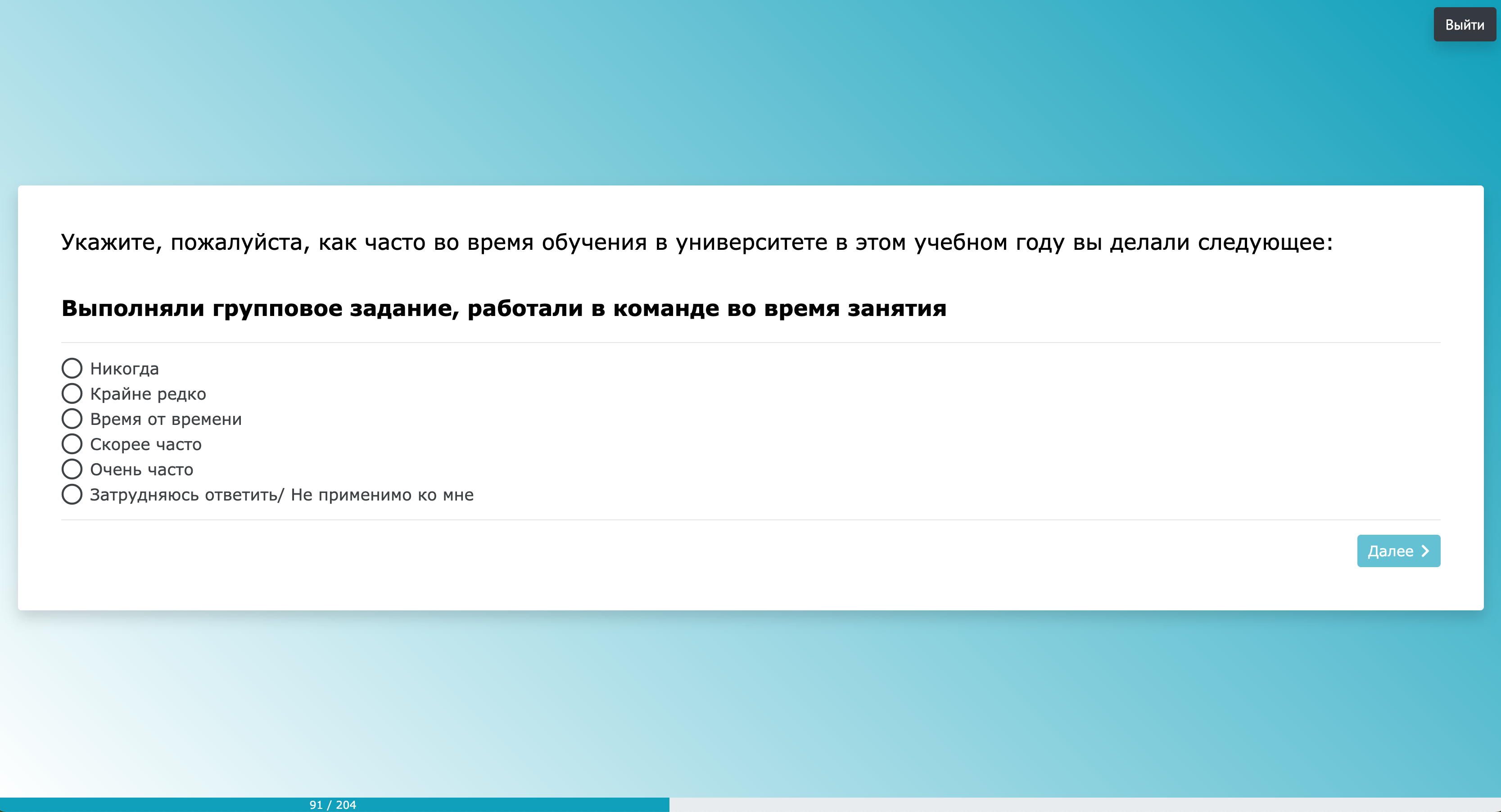
**

**
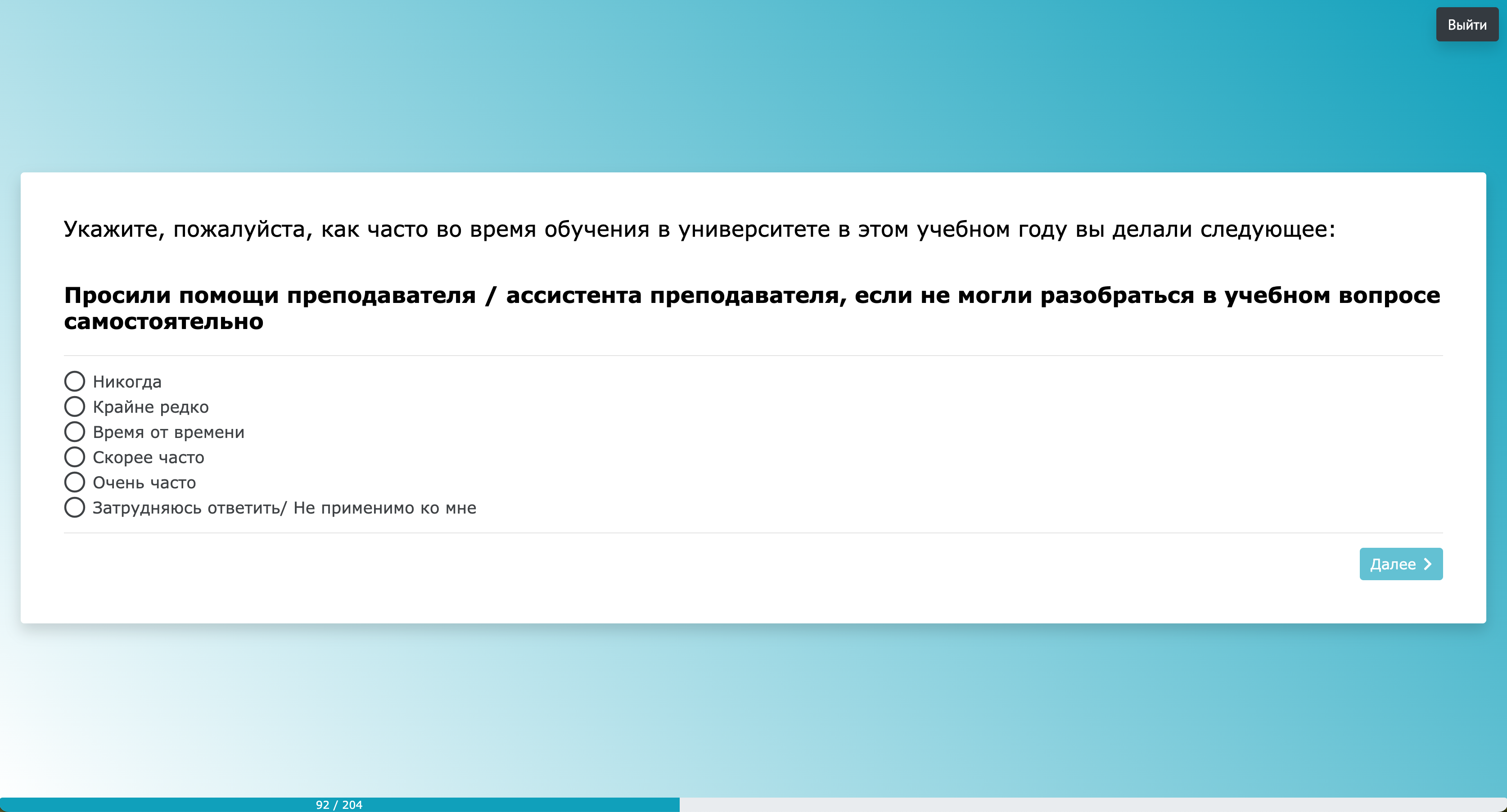
**

**
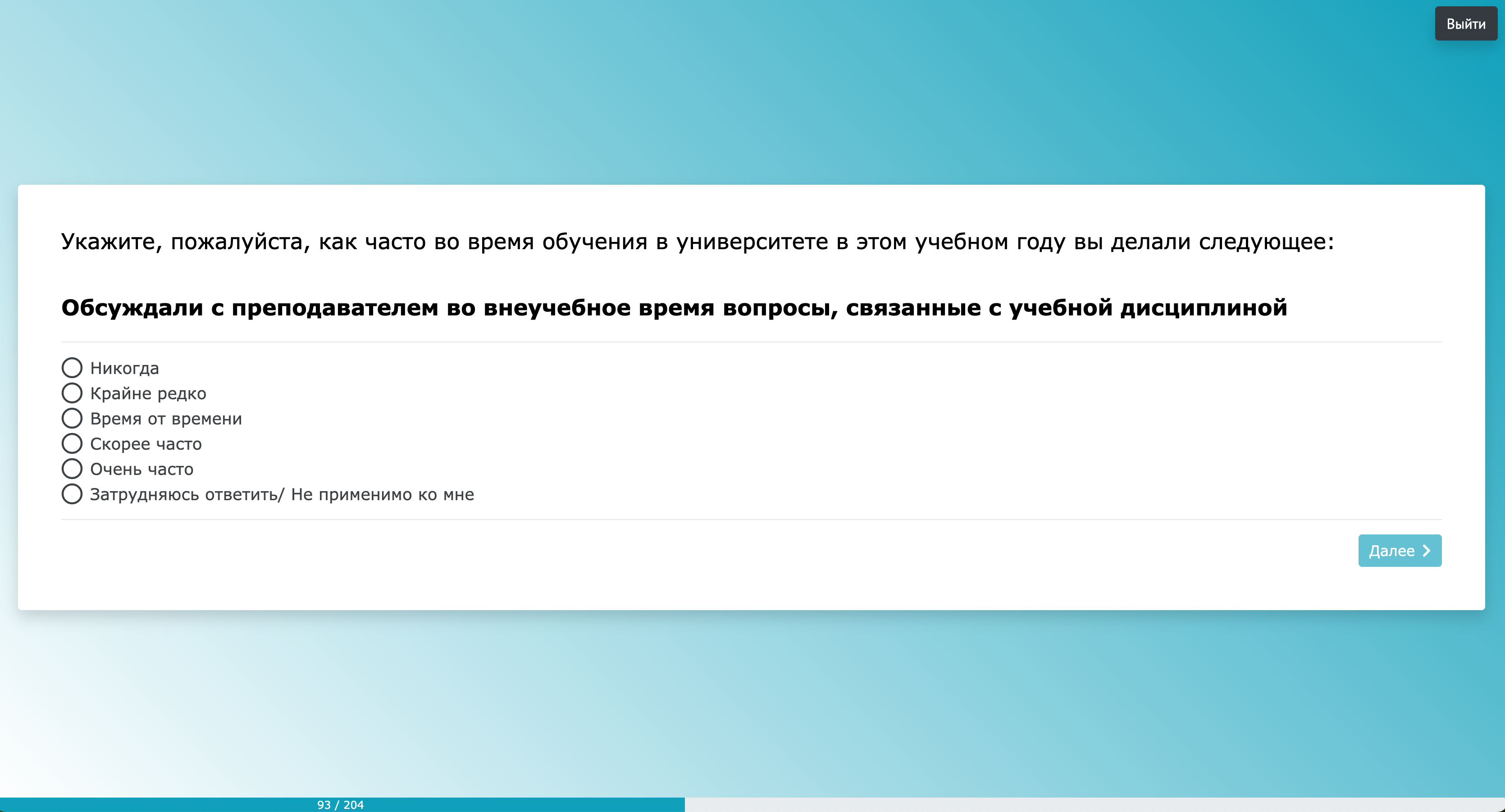
**

**
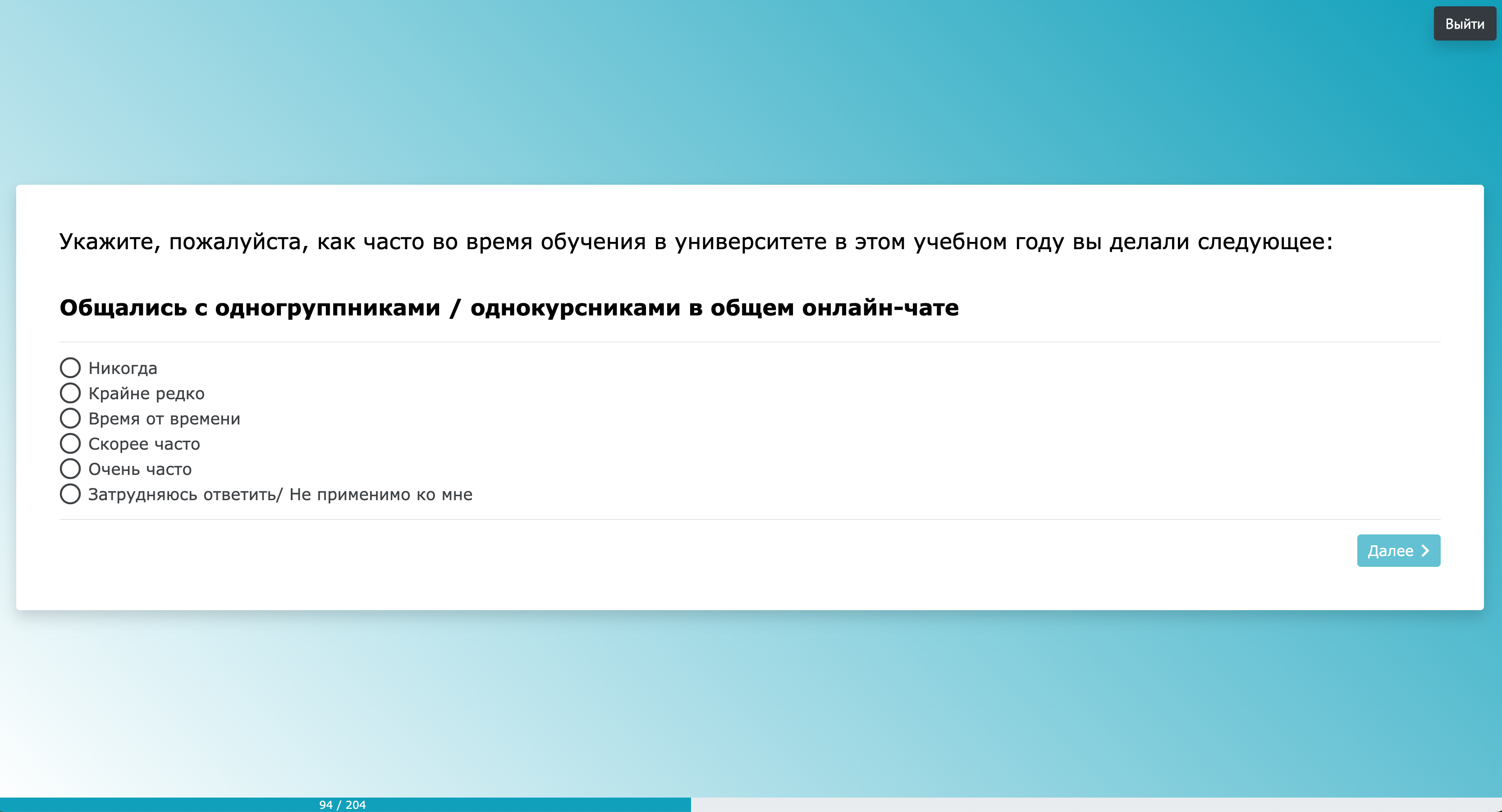
**

**
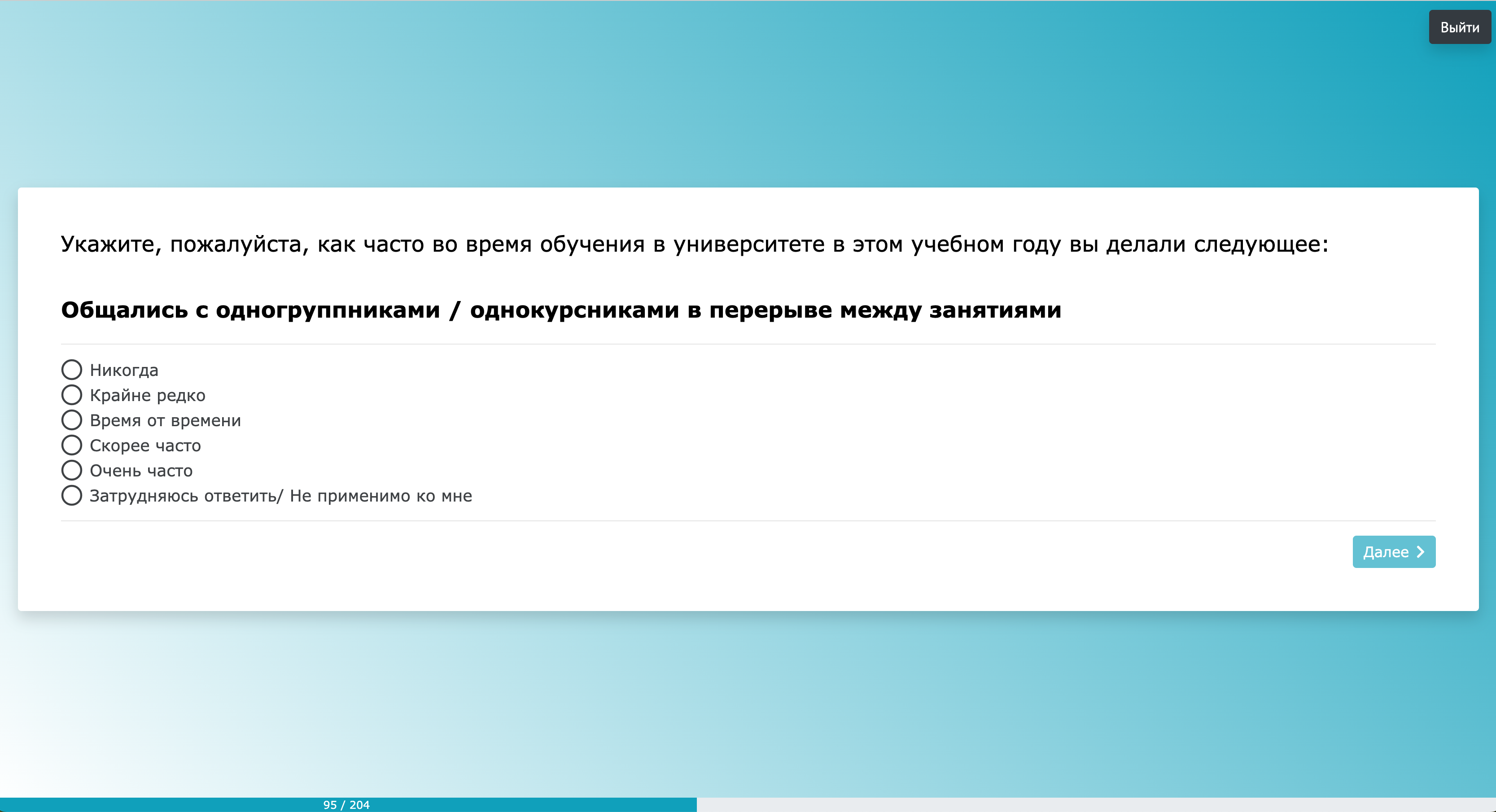
**

**
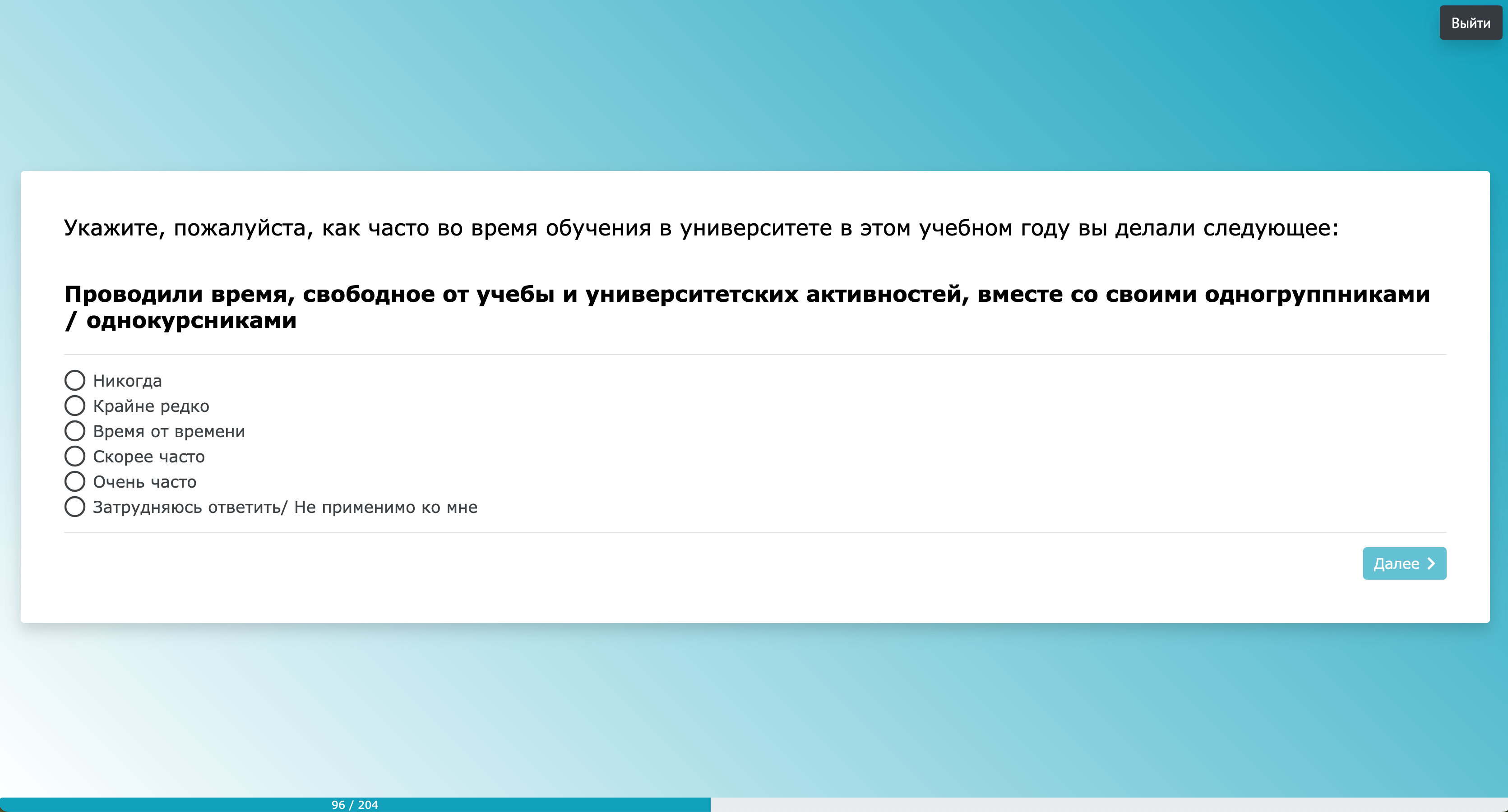
**

**
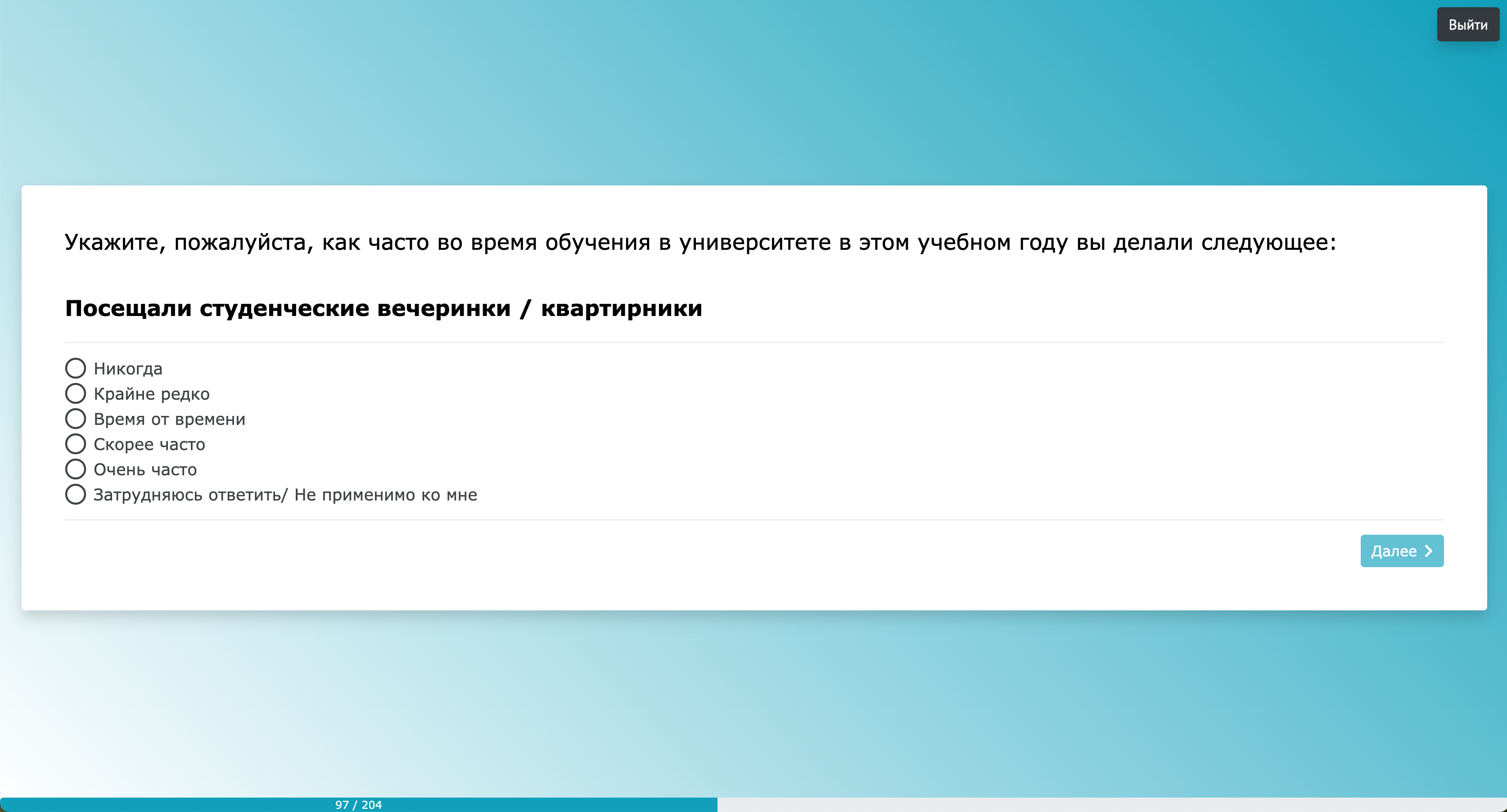
**

**
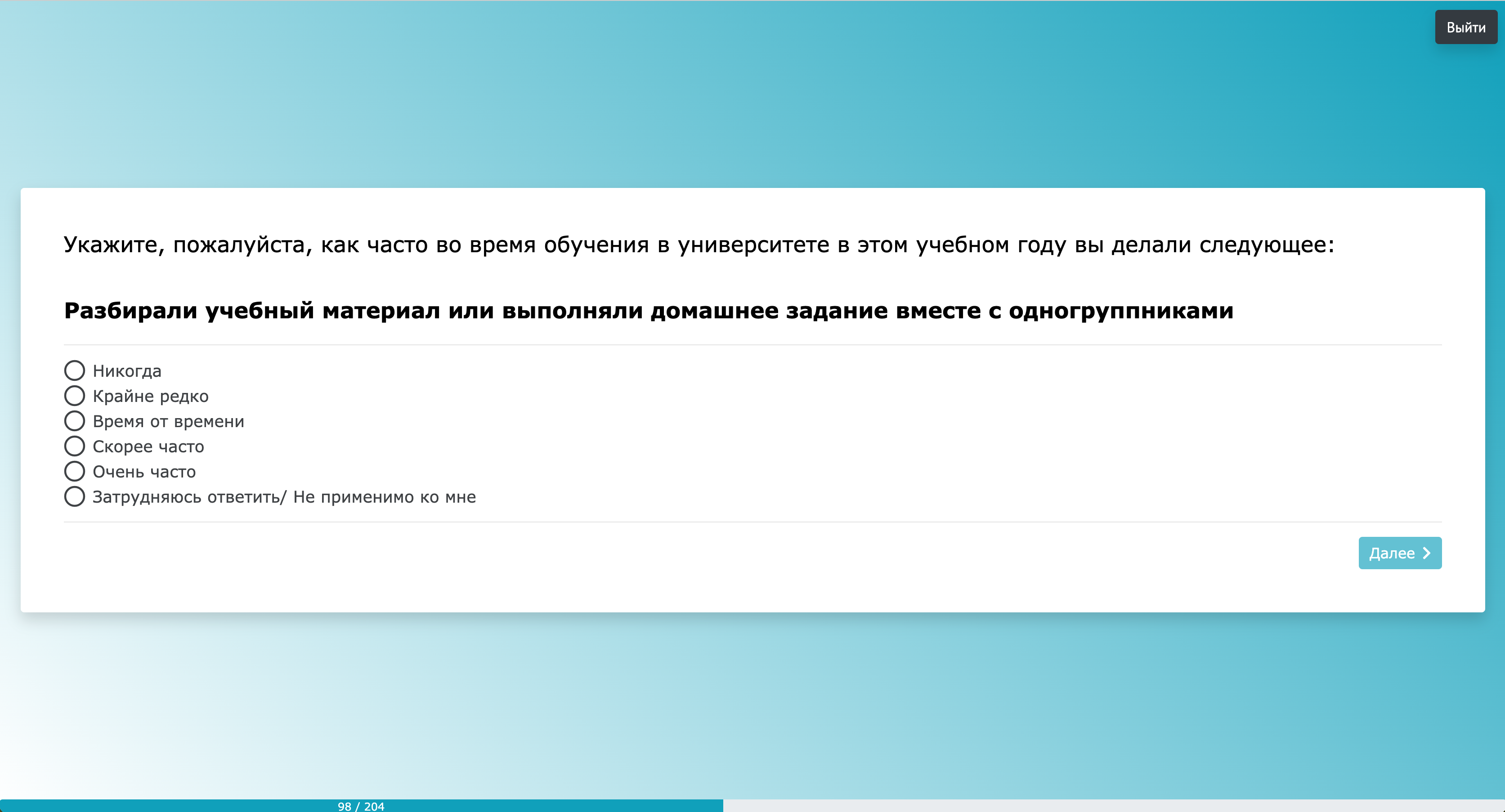
**

**
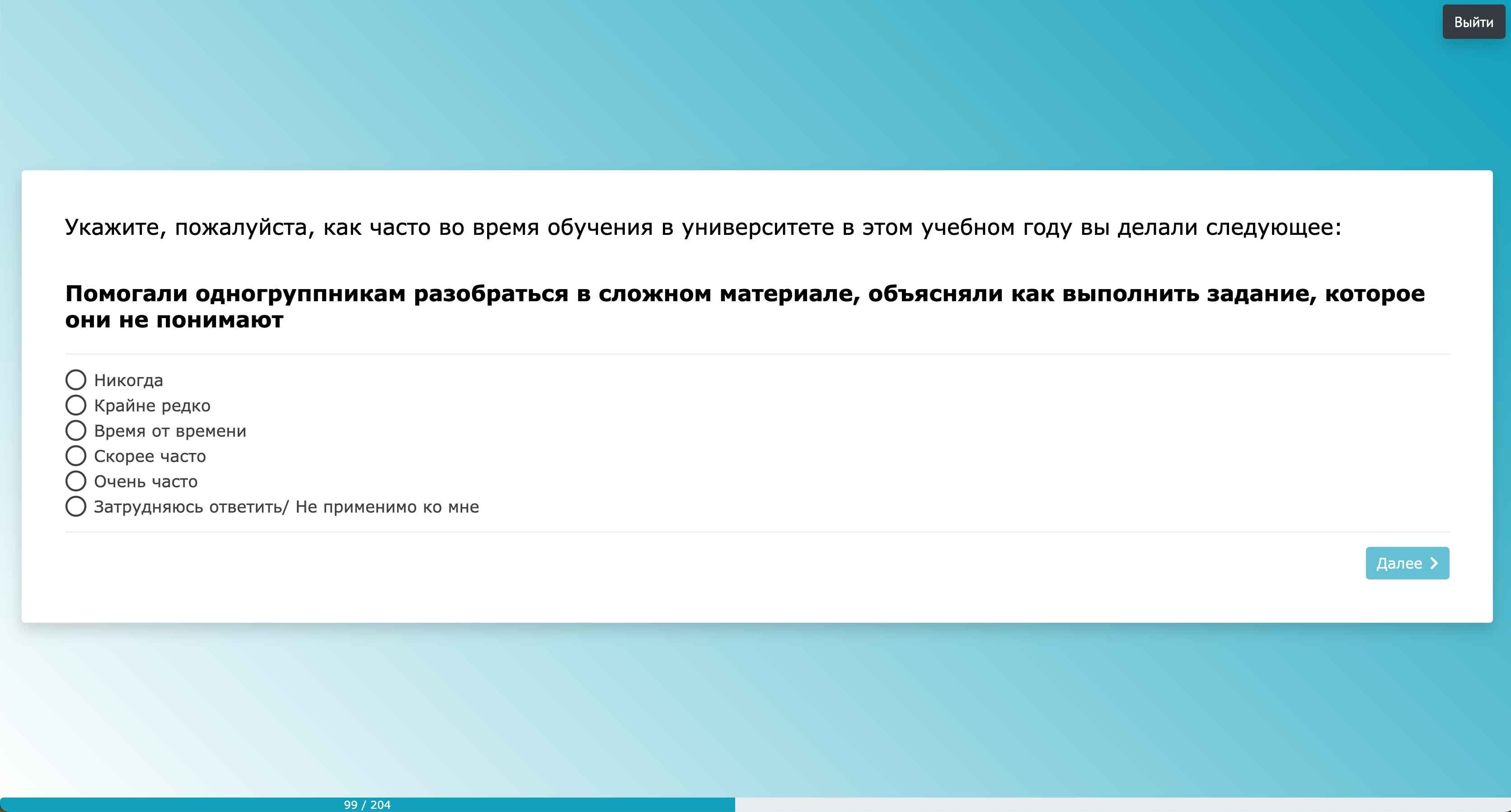
** **
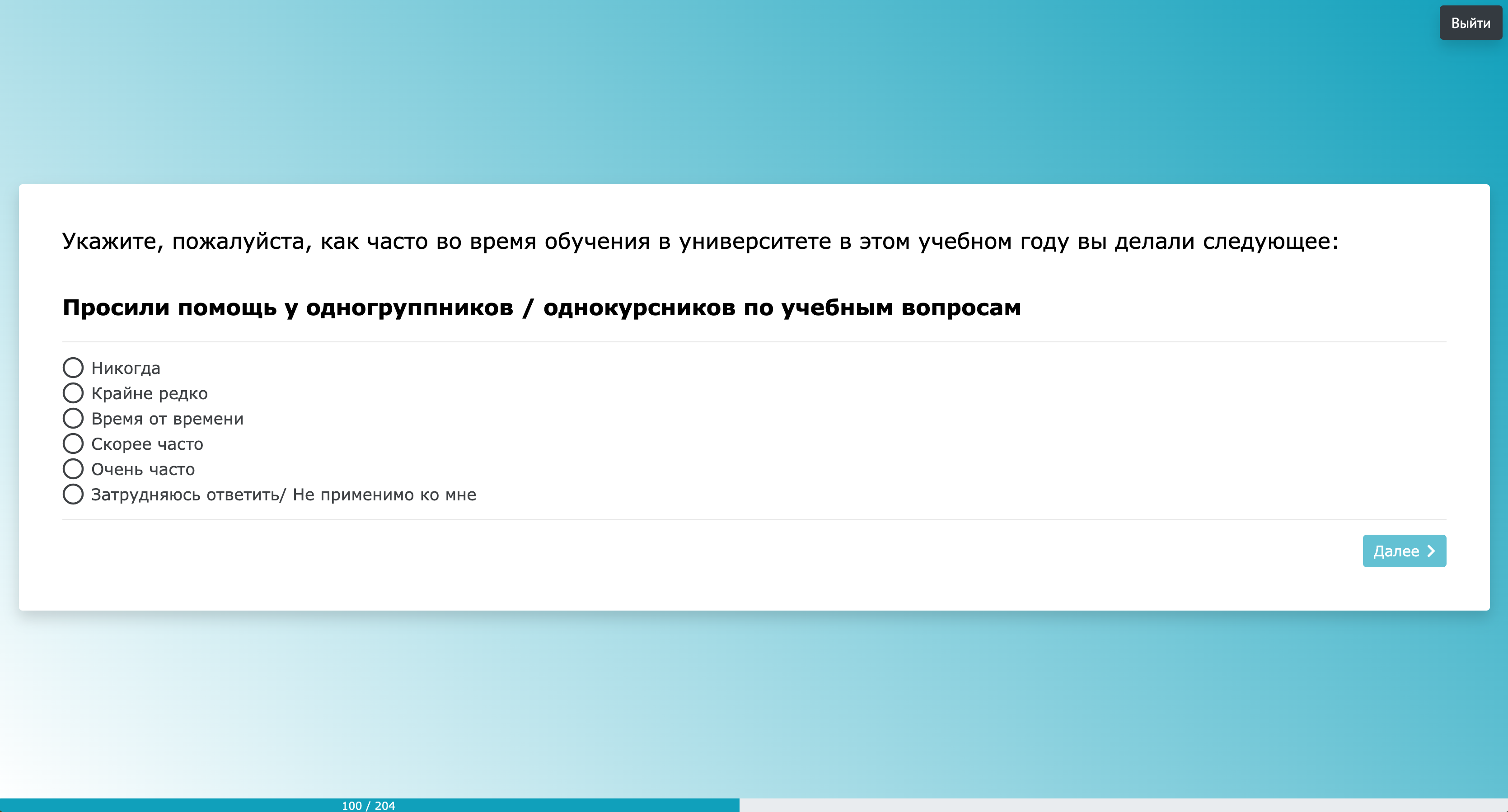
**

**
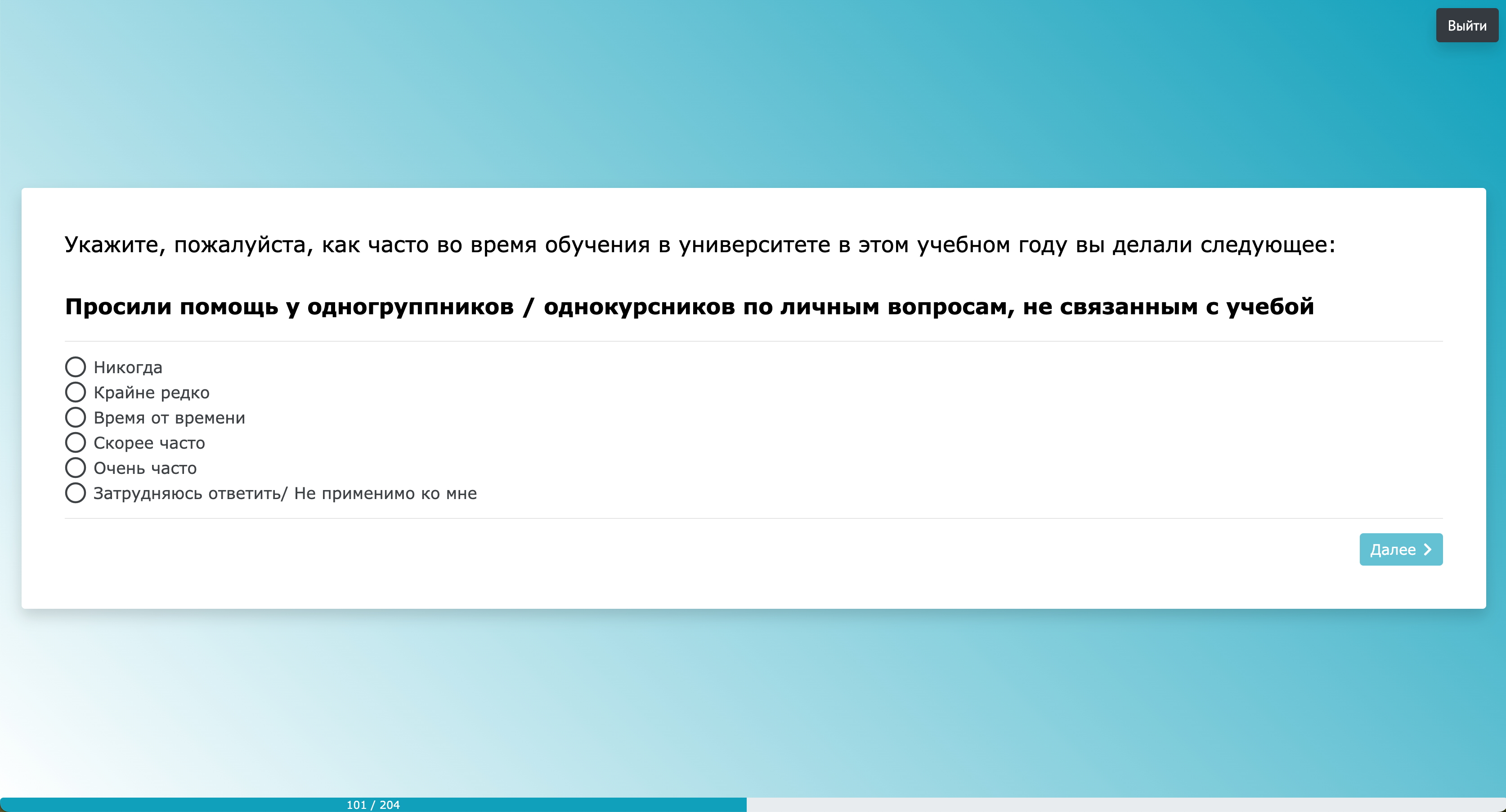
**

**
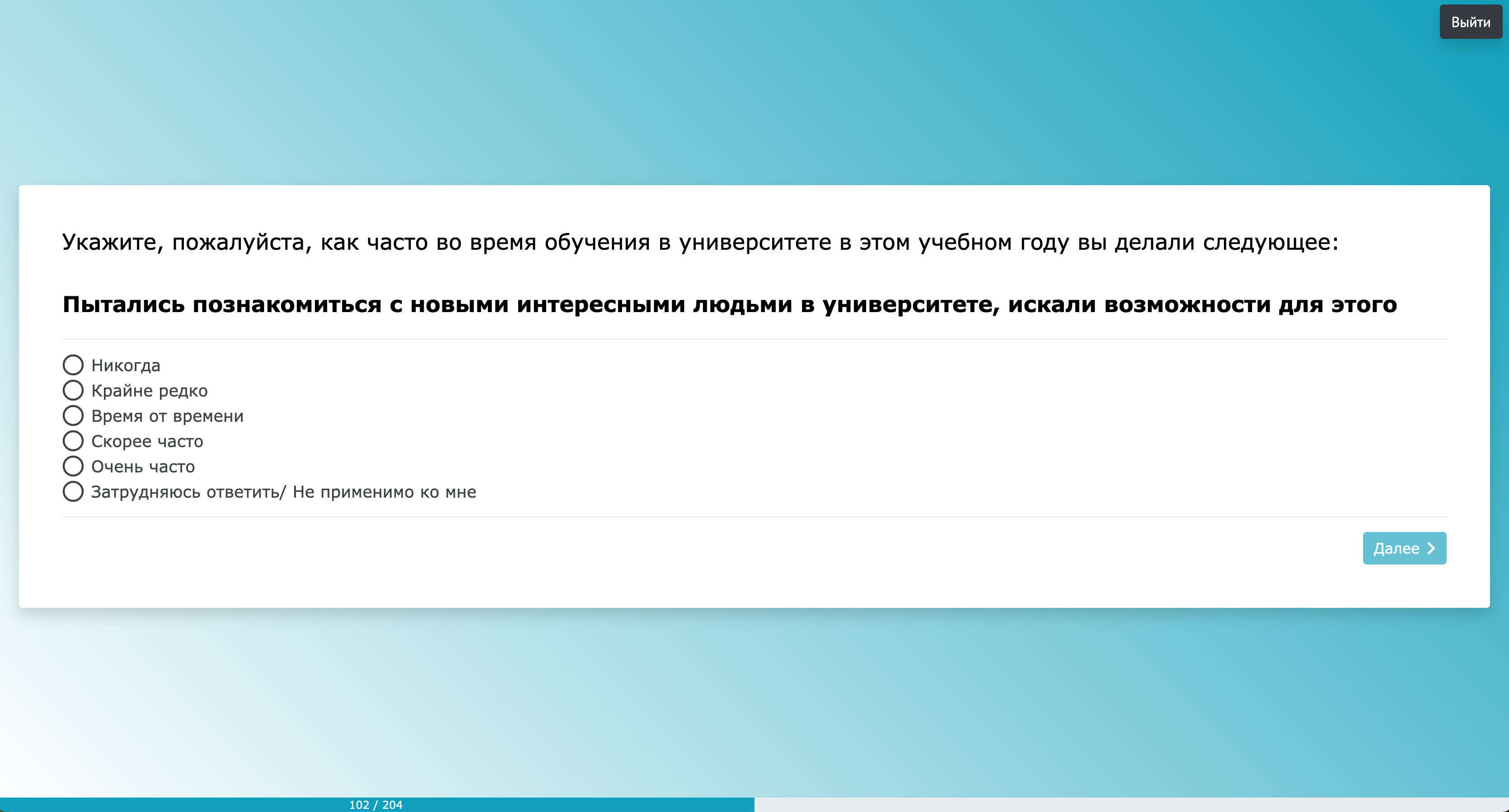
**

**
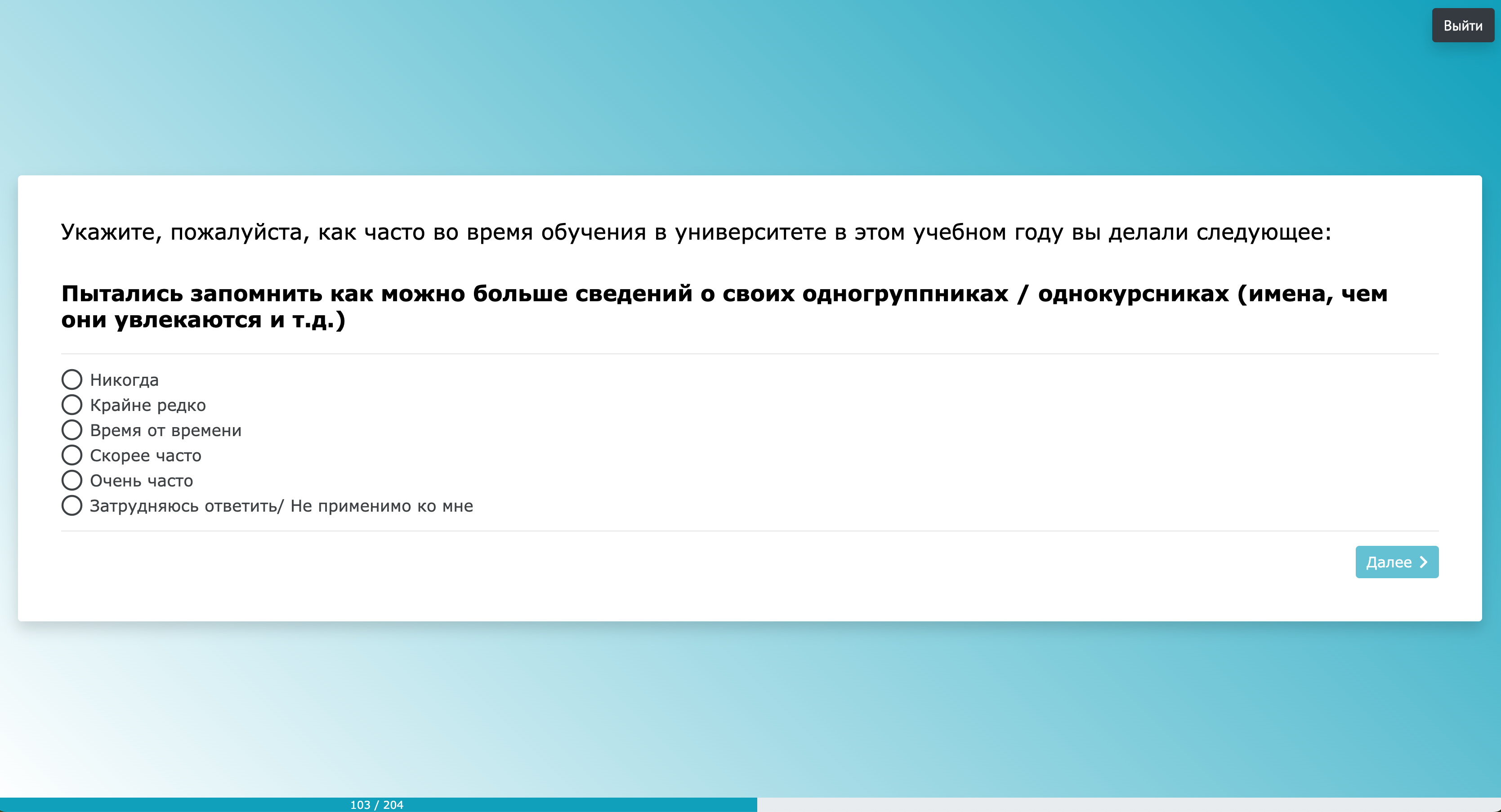
**

**
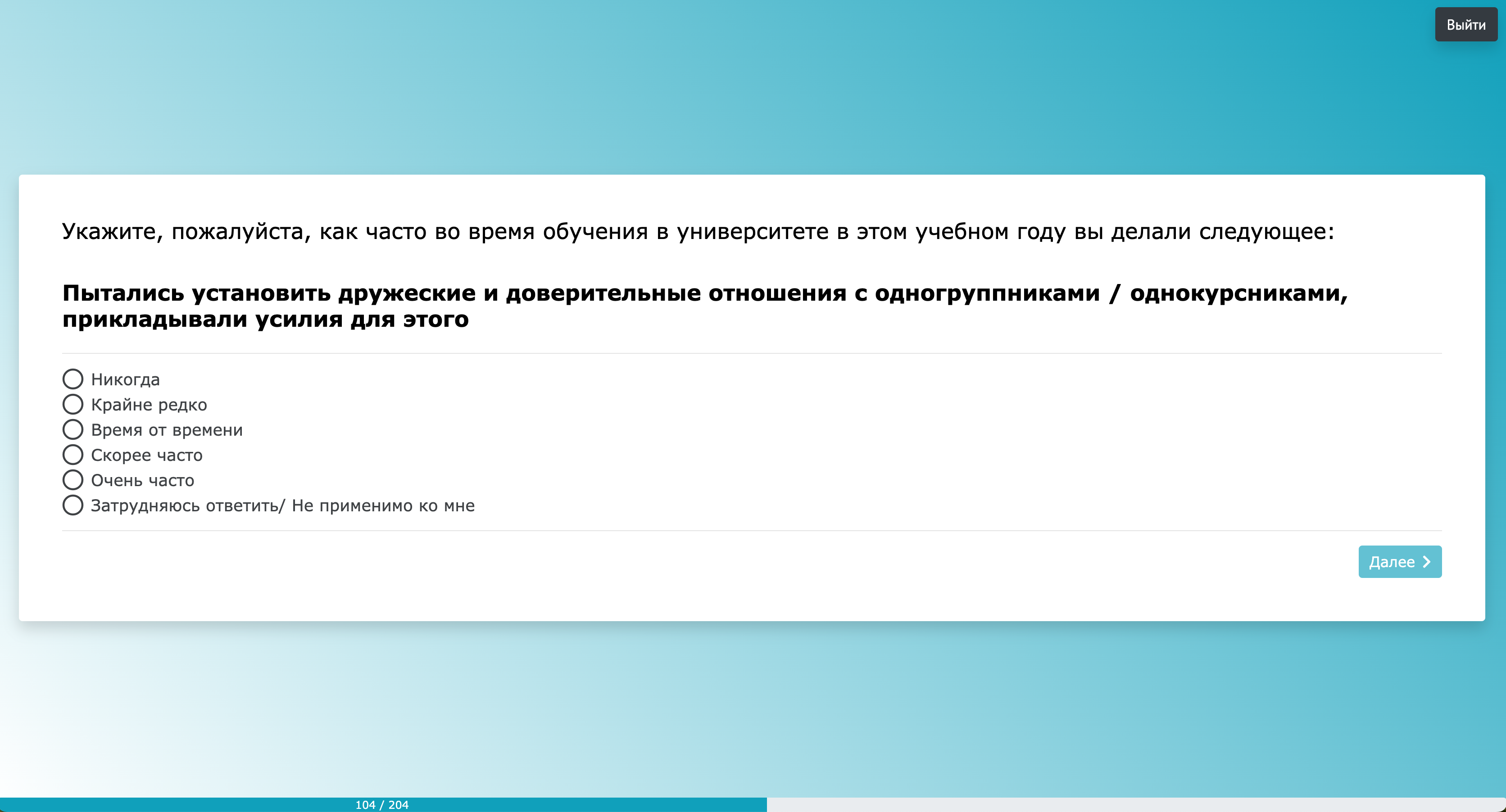
**

**
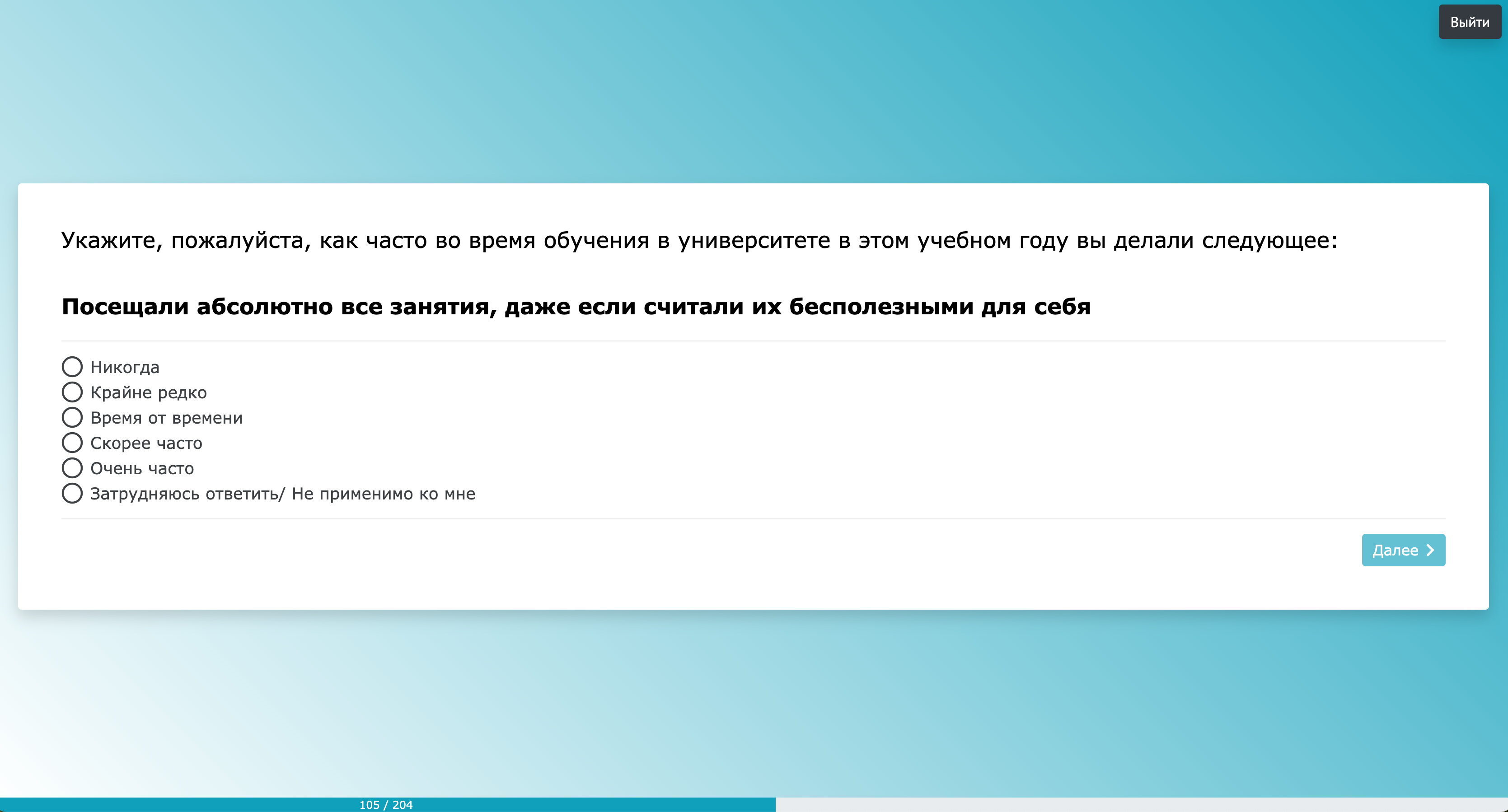
**

**
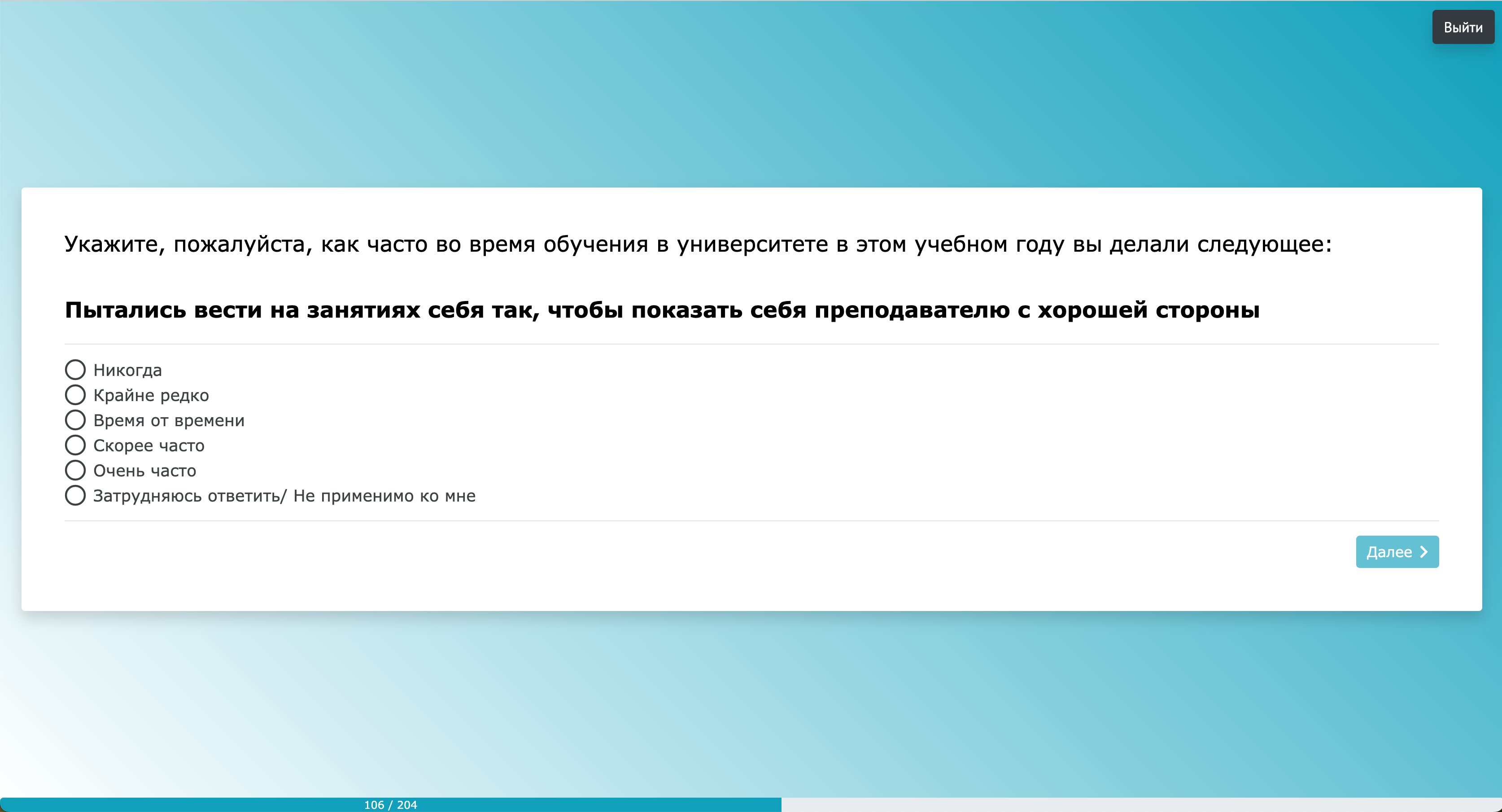
**

**
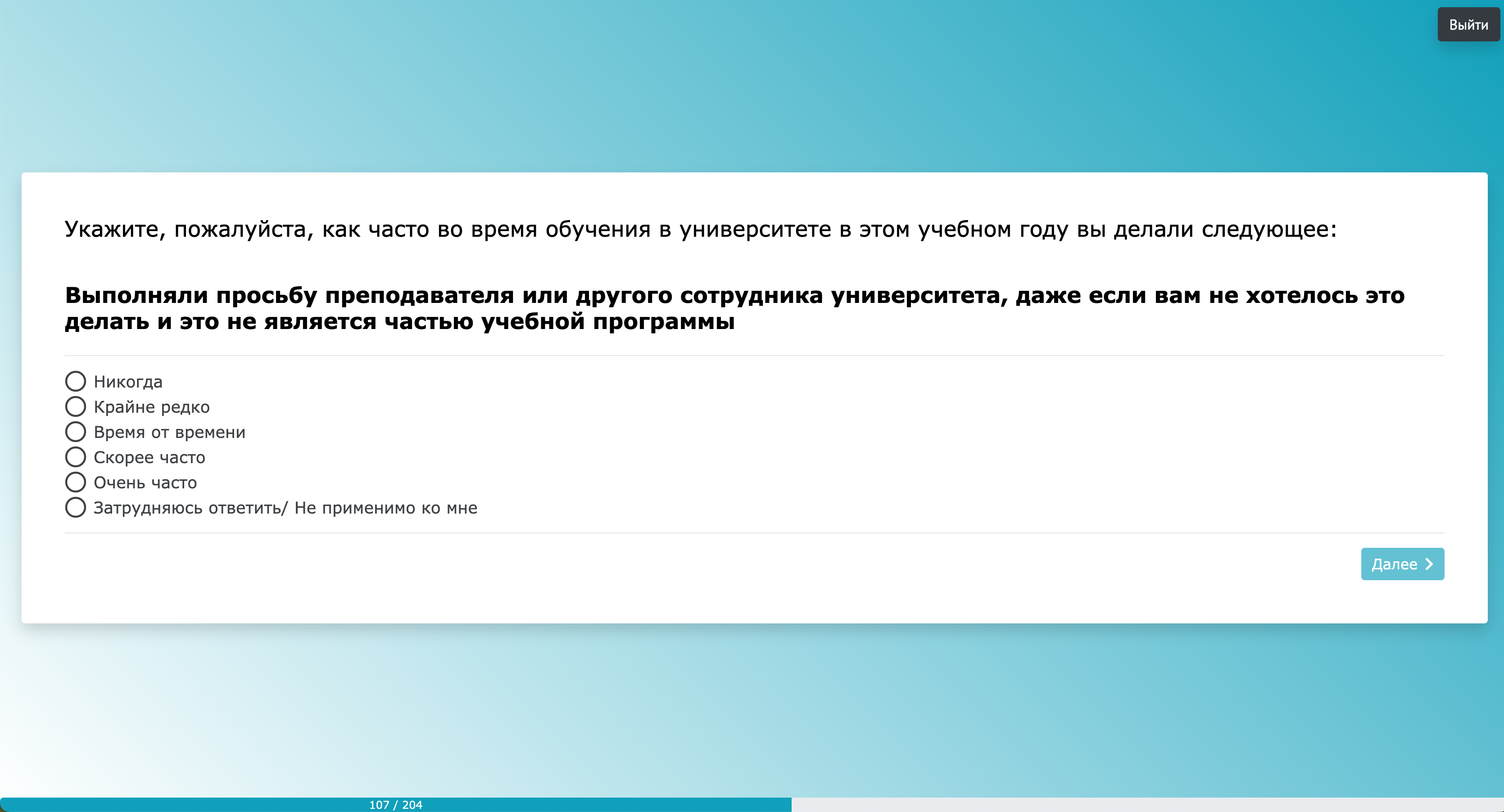
**

**
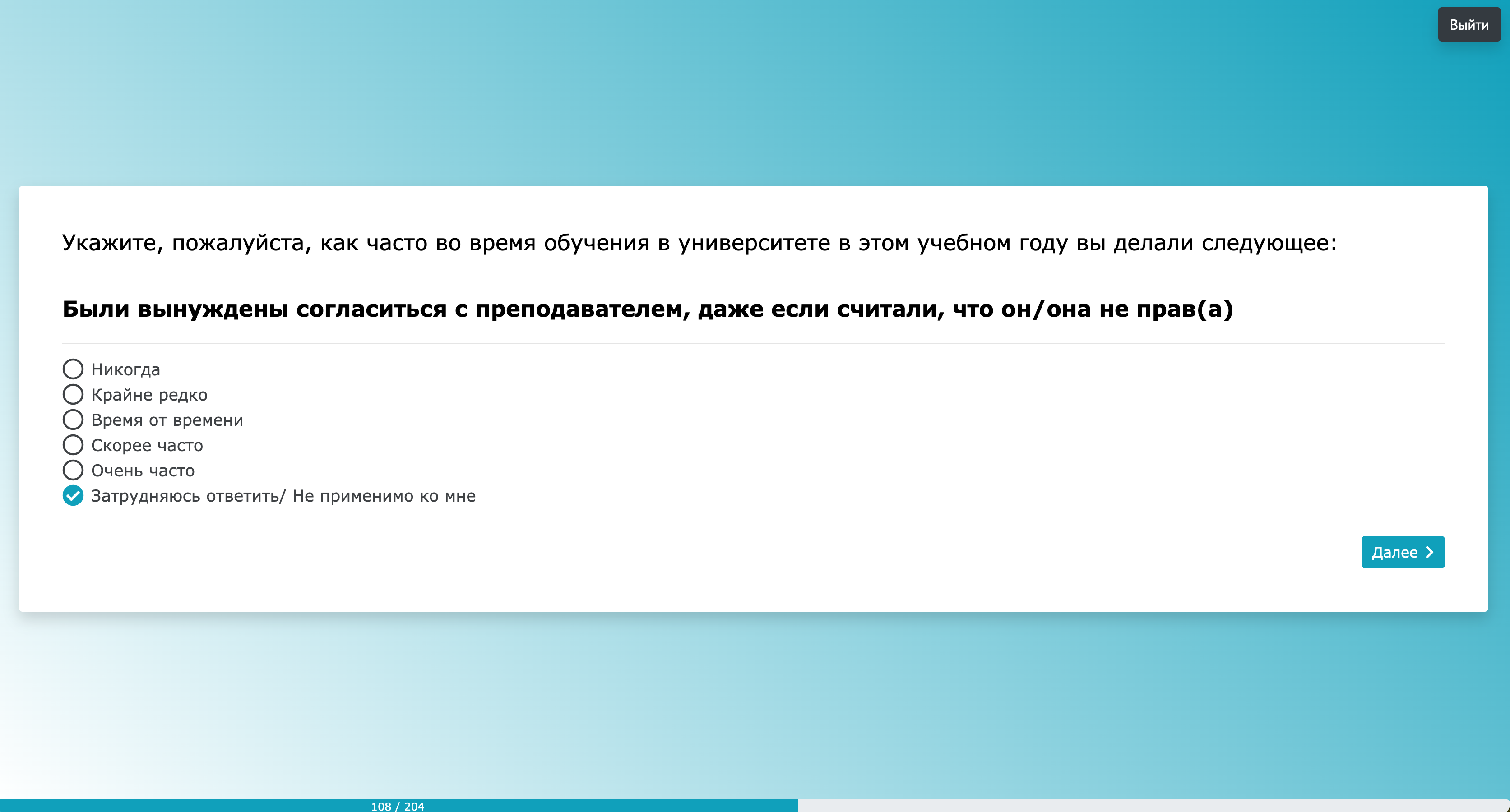
**

**
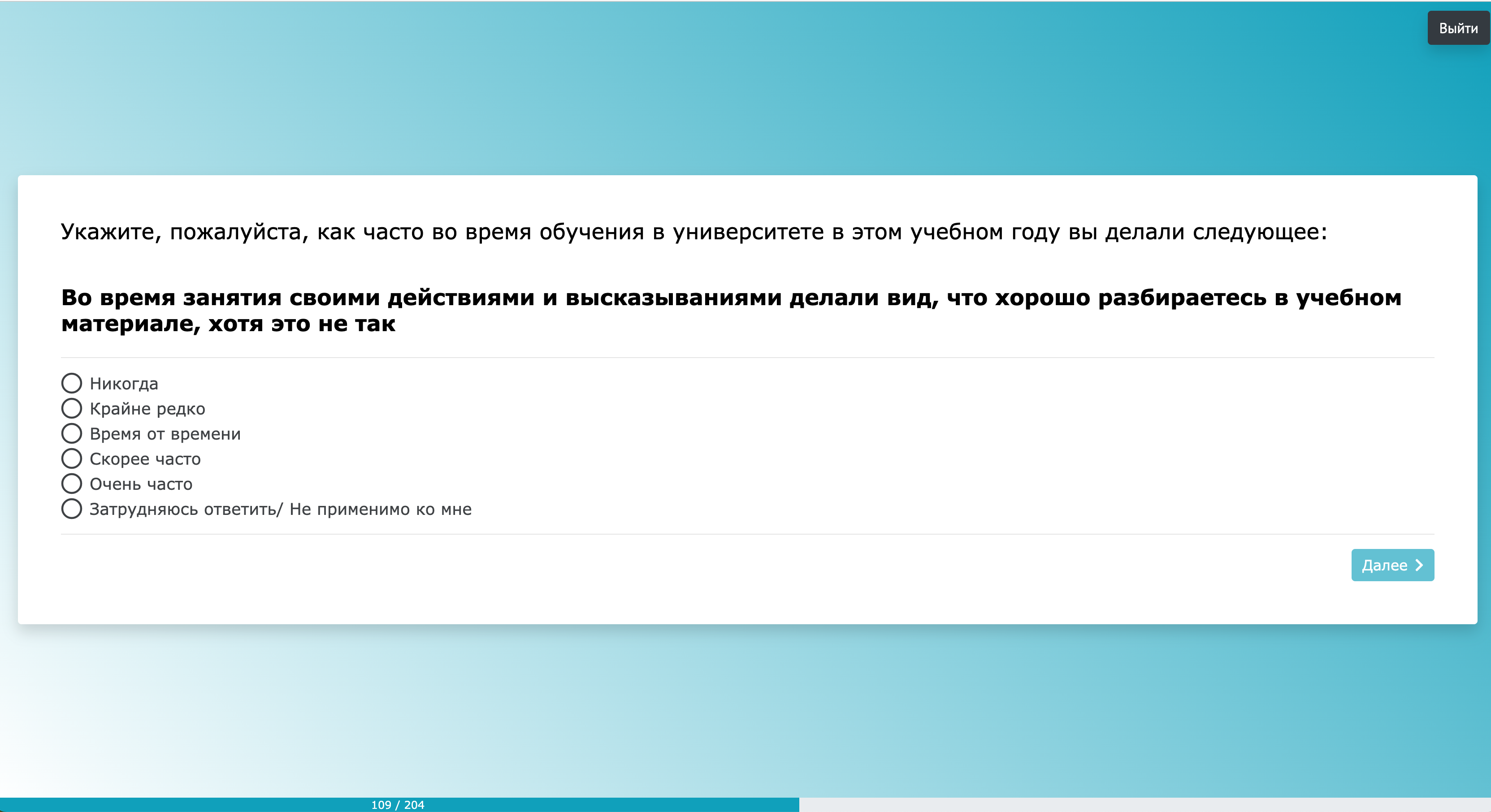
**

**
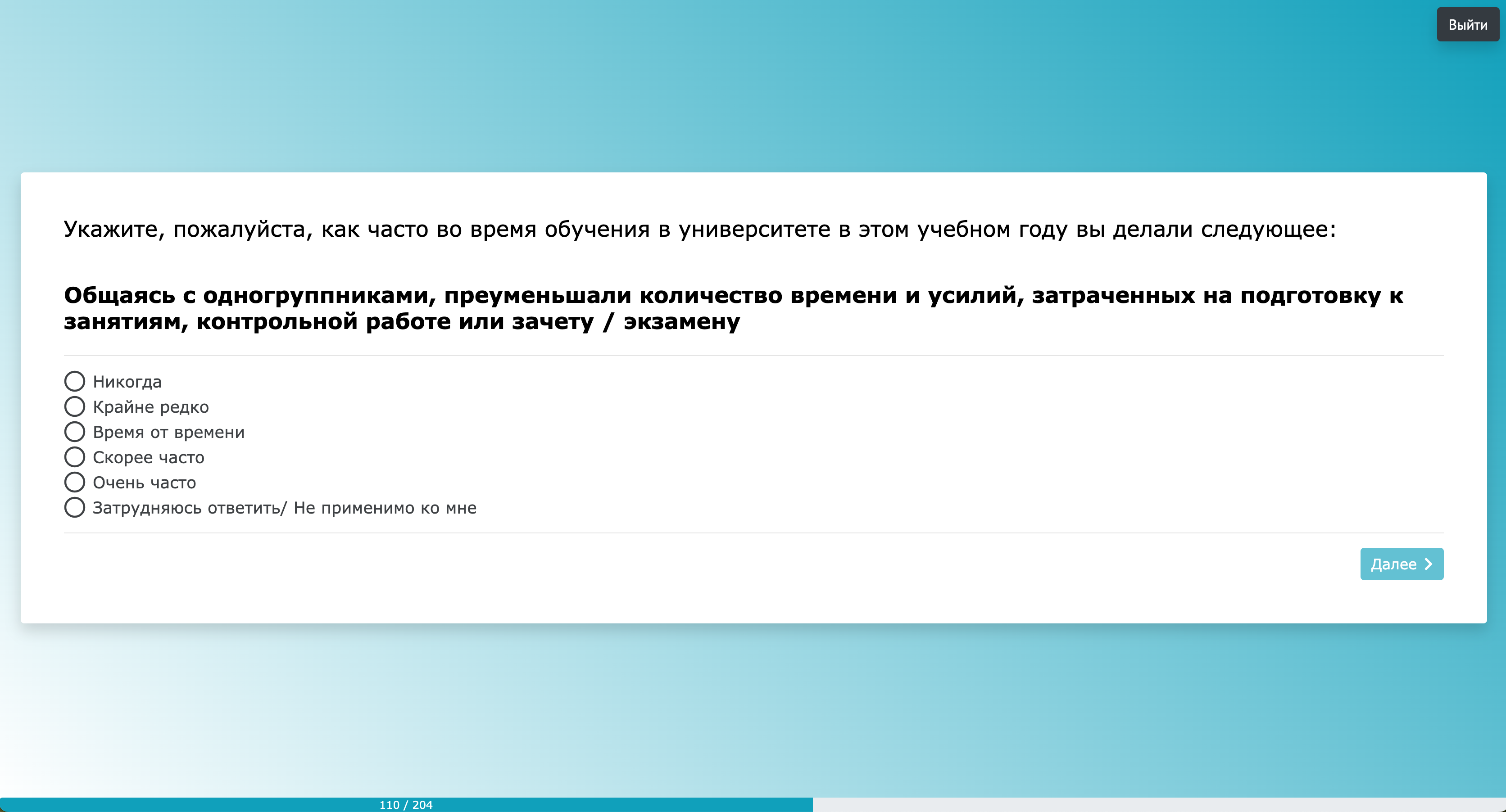
**

**
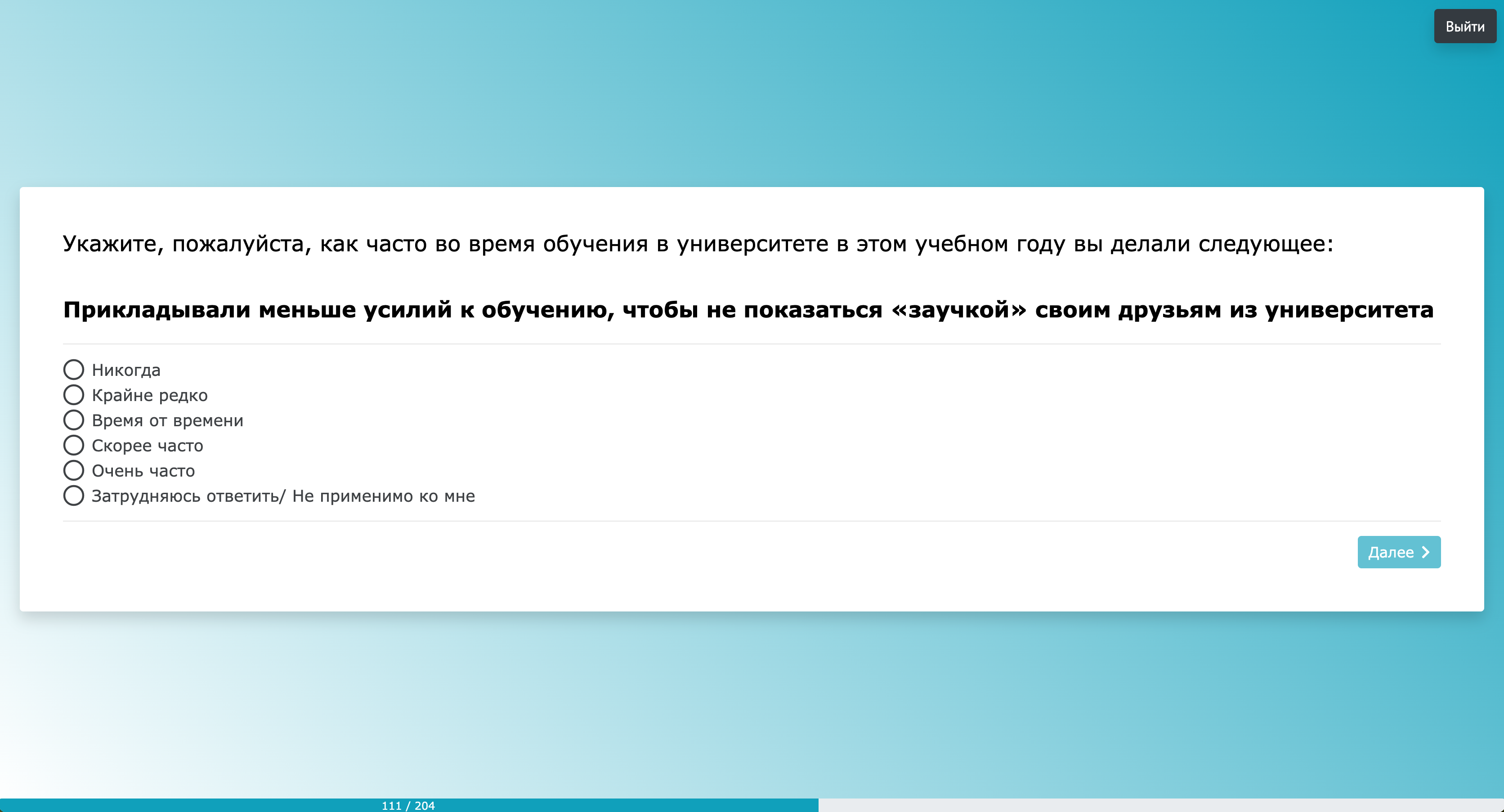
**

**
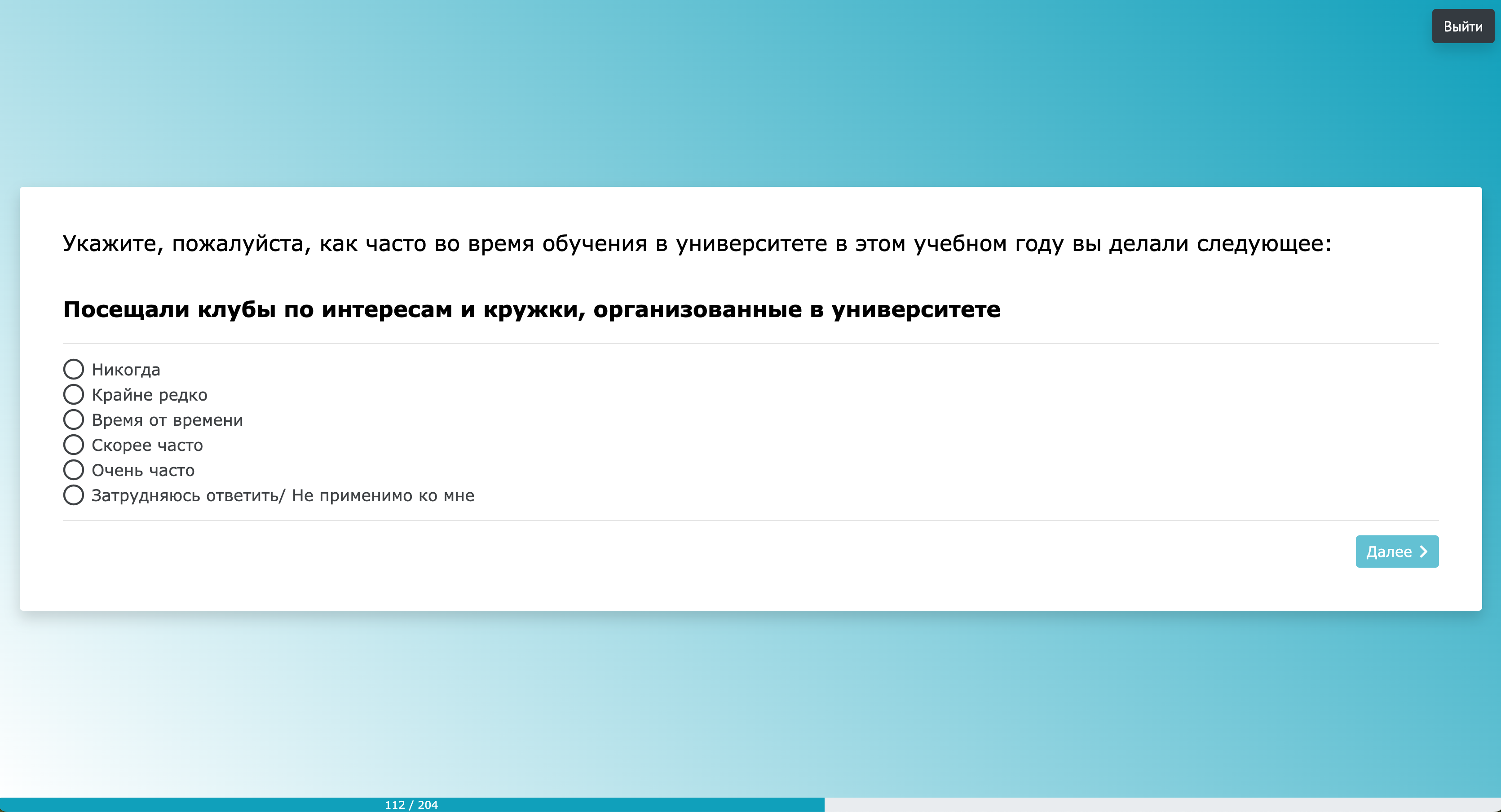
**

**
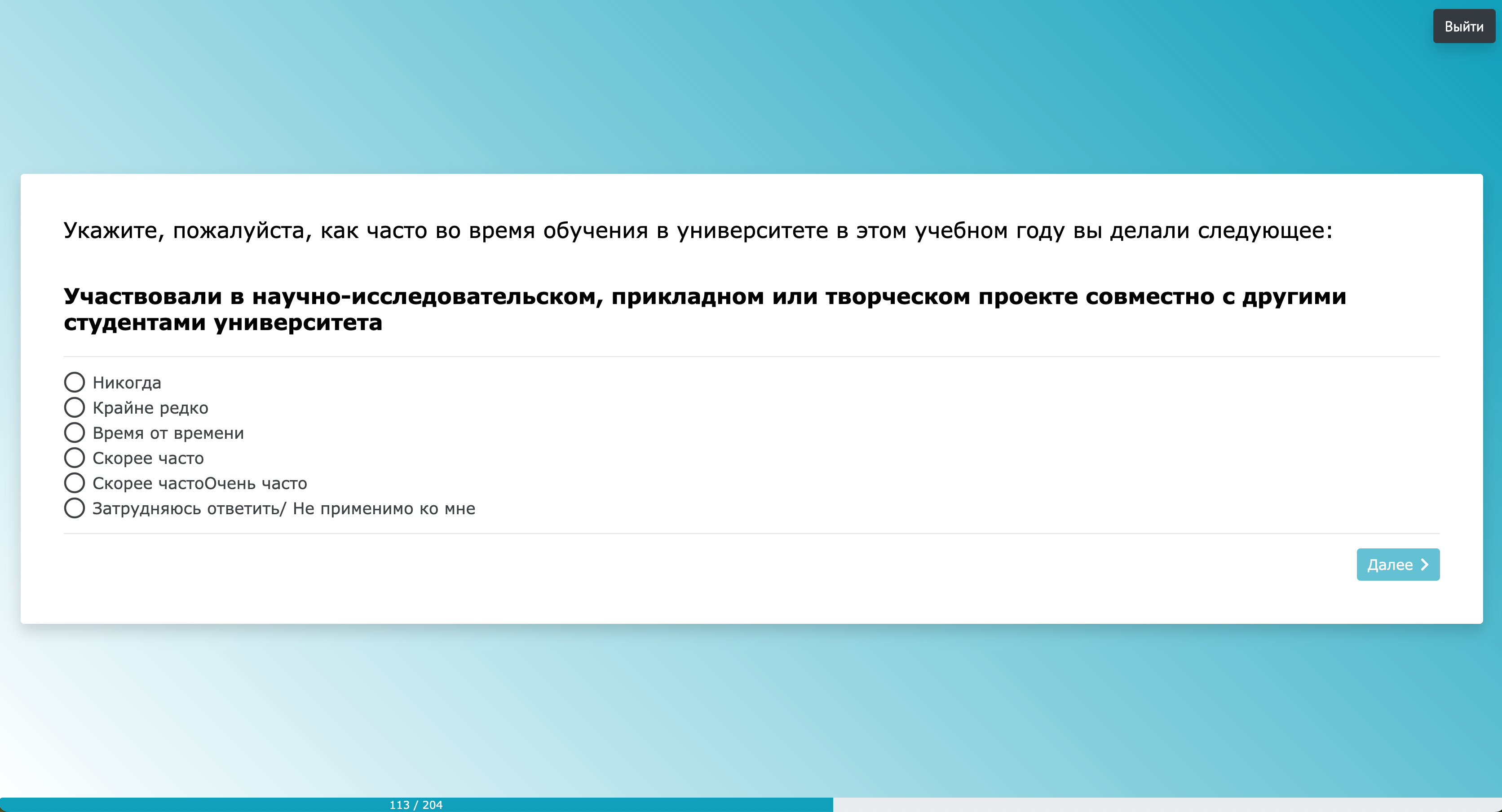
**

**
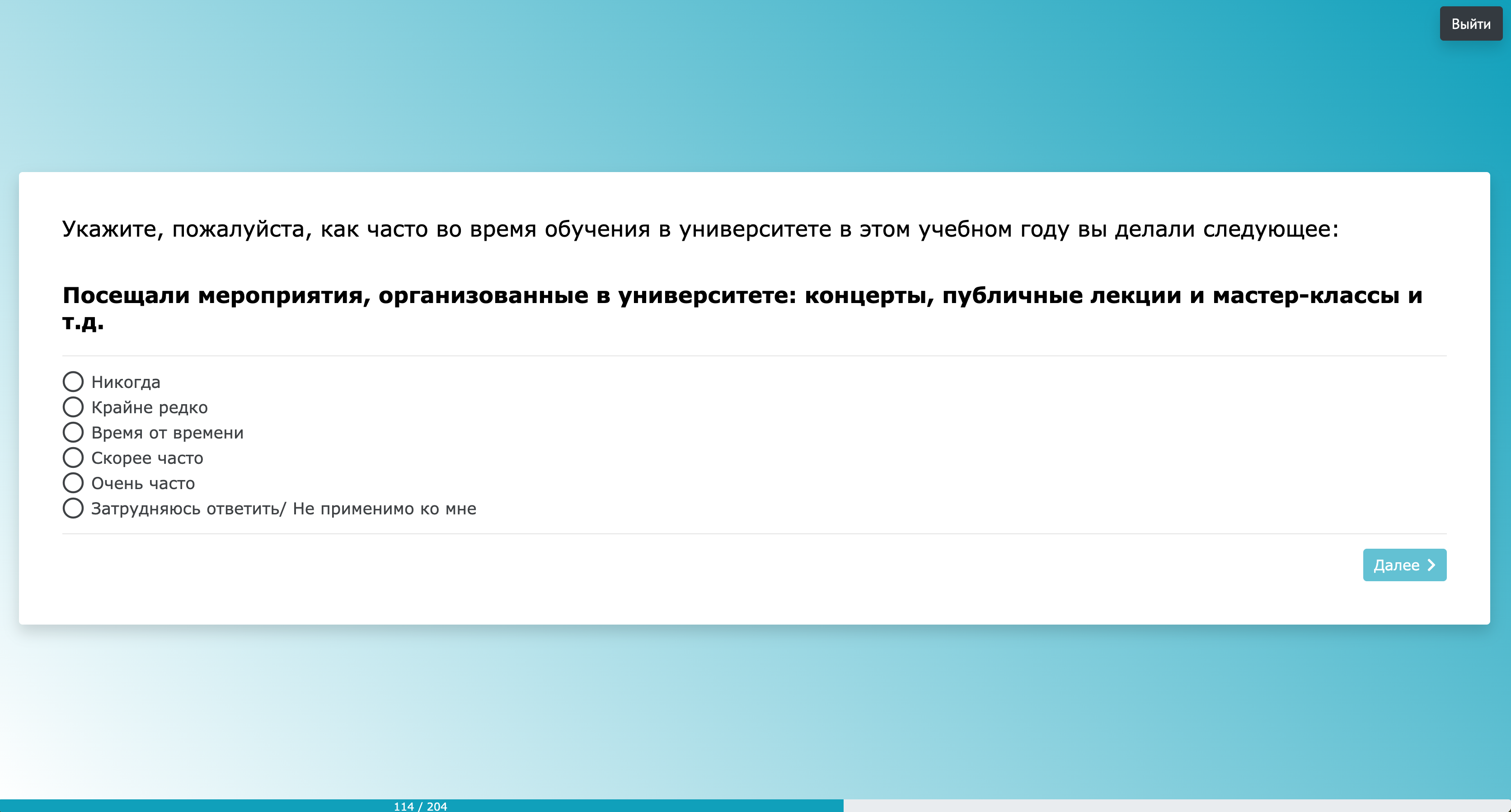
**

**
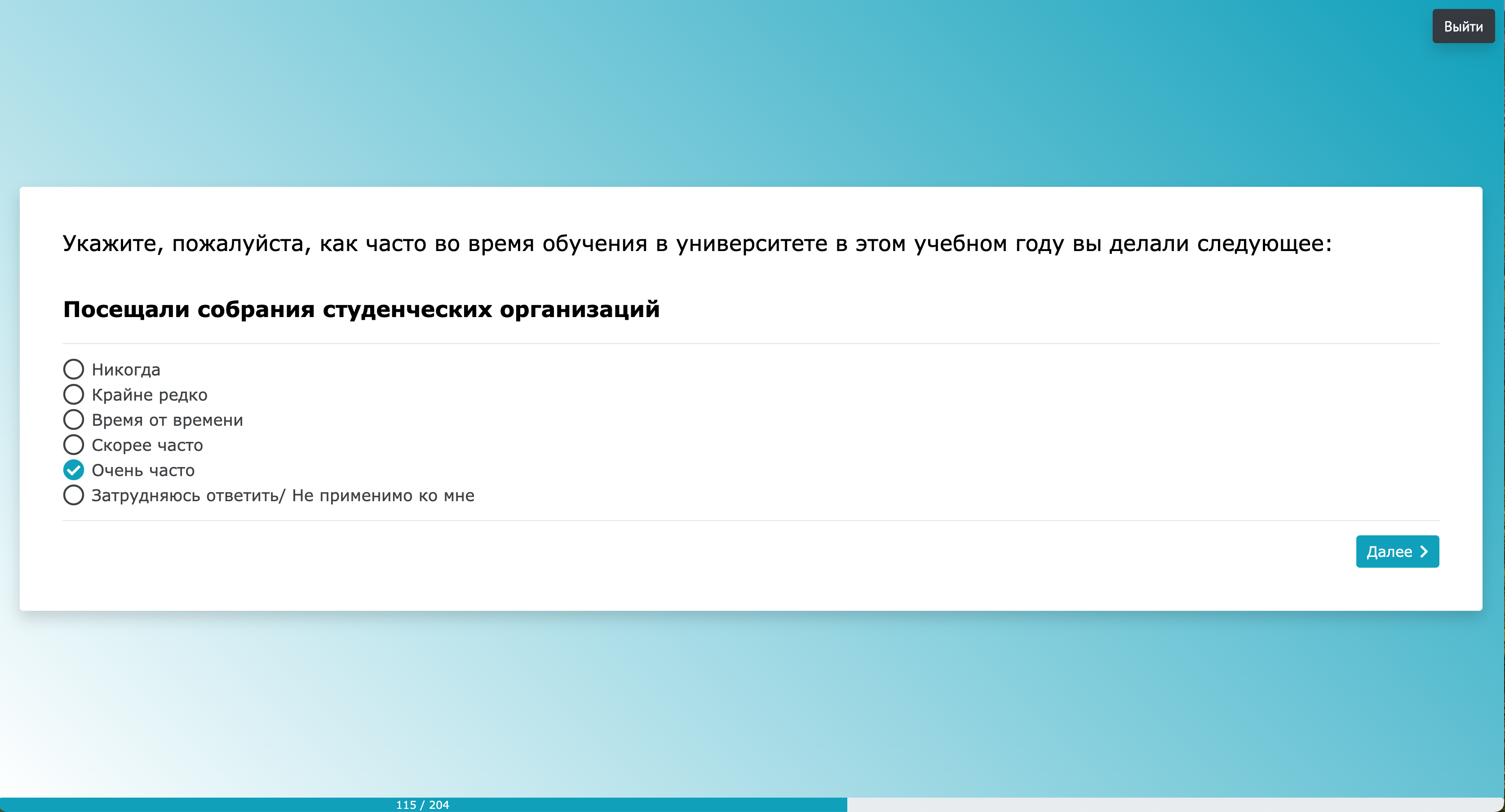
**

**
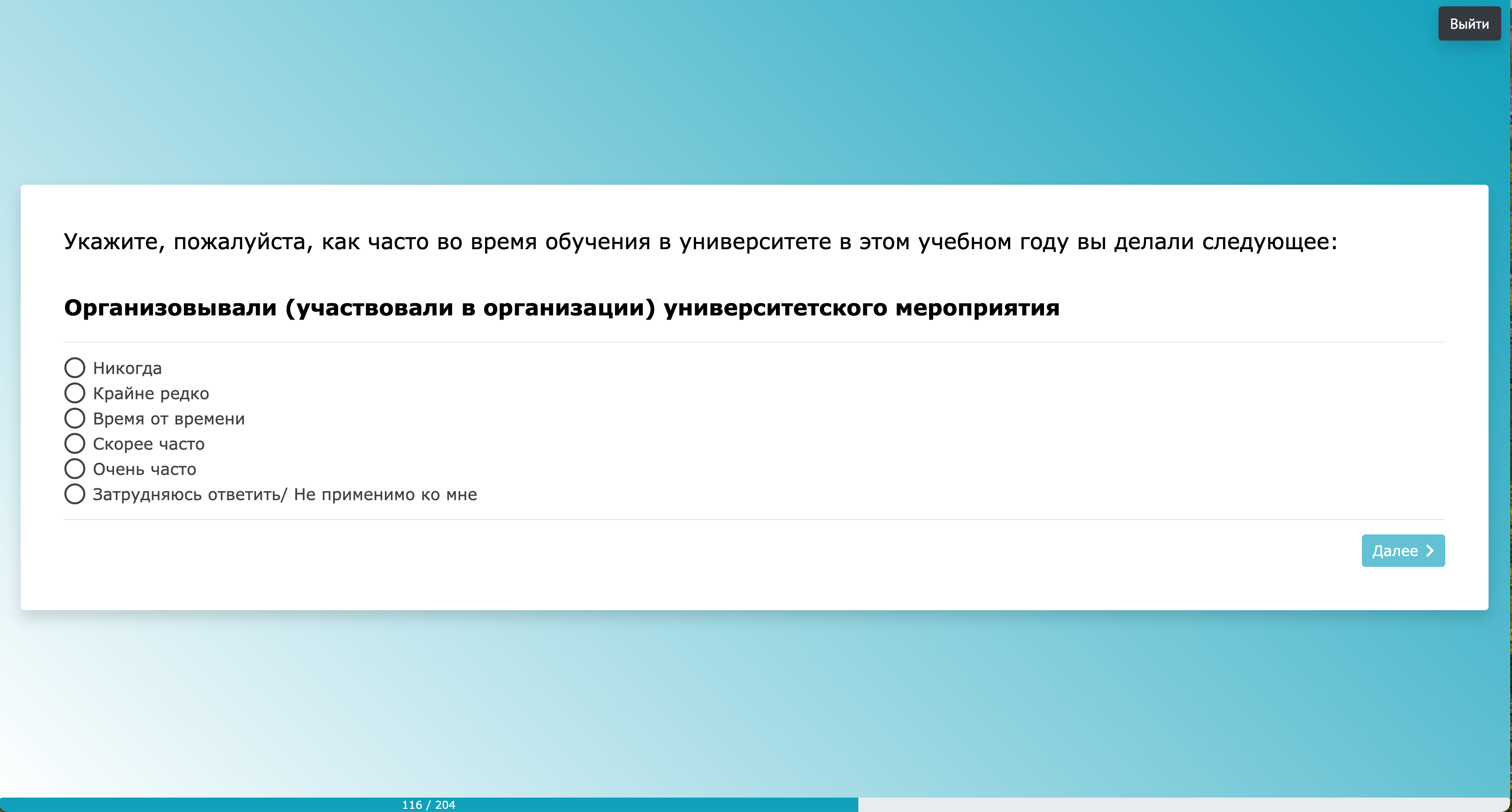
**

**
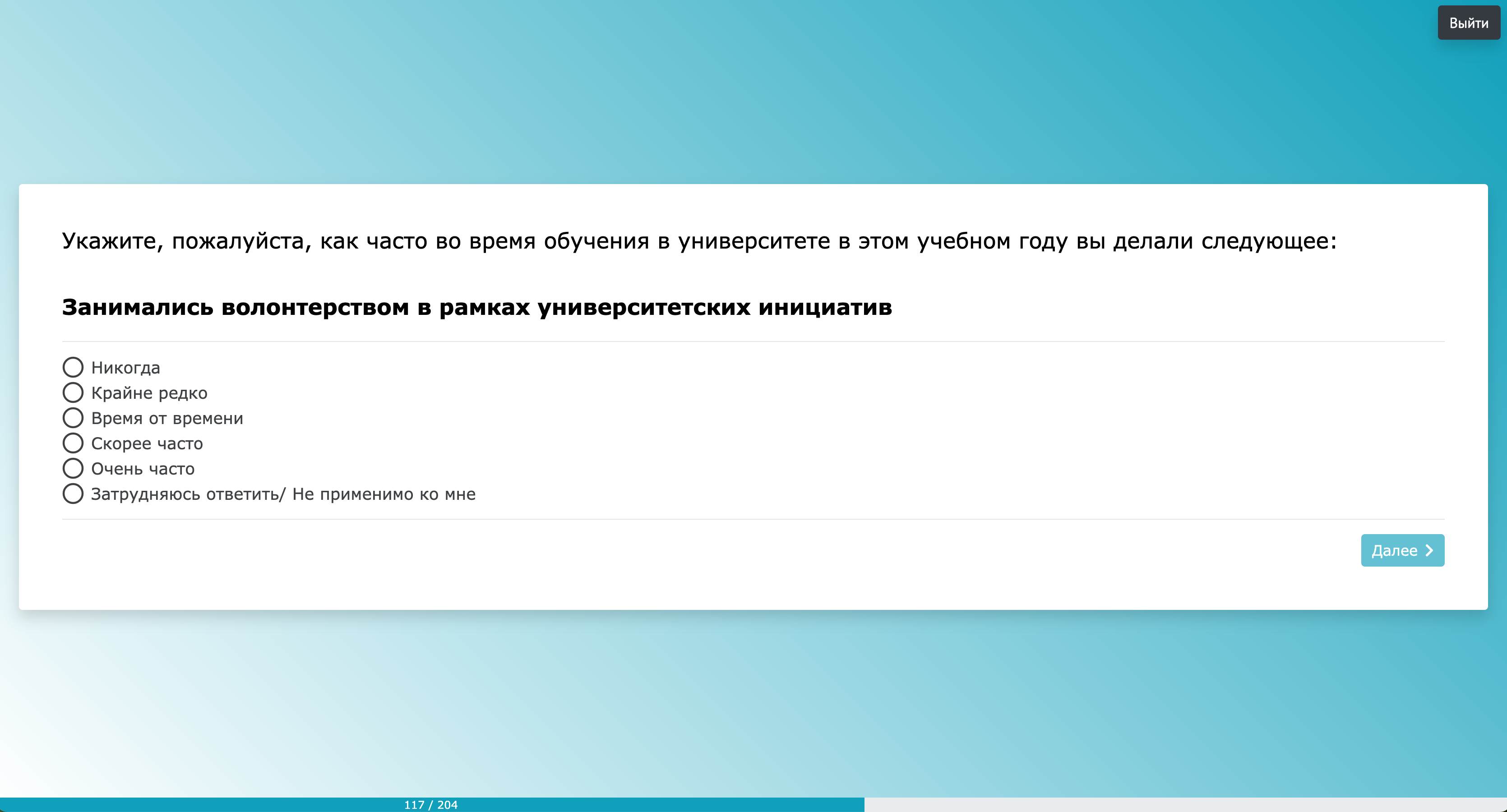
**
