# Supplementary material for: Student behavior at university: The development and validation of a 10-dimensional scale
Source: PLoS One. 2024 Nov 15;19(11):e0313357. doi: 10.1371/journal.pone.0313357 (PMC11567547; doi:10.1371/journal.pone.0313357)
Supplement: Attachment — Submitted filename: Manuscript_07_22 review EMP.docx [file pone.0313357.s008.docx]

**Student behavior at university: the development and validation of a 10-dimensional scale**

Natalia Maloshonok^1*^, Kseniia Vilkova^1^

^1^ Center for Sociology of Higher Education, HSE University, Moscow, Russia

^*^ Corresponding author

E-mail: [nmaloshonok@hse.ru](mailto:nmaloshonok@hse.ru)

Author Contributions

Conceptualization: NM.

Formal analysis: NM.

Methodology: NM.

Project administration: KV.

Writing – original draft: NM.

Acknowledgments

Support from the Basic Research Program of the National Research University Higher School of Economics (HSE University) is gratefully acknowledged.

**Abstract**

This study proposes the multidimensional concept of ‘student behavior at university’ and methodology for its quantitative investigation. Unlike previous concepts related to aspects of the student experience, the idea of student behavior considers the combinations and interrelations of individual and environmental characteristics affecting student experience and outcomes. It provides a new lens for viewing student experience at university which highlights the multifacetedness of this phenomenon and the diversity of possible patterns of student behavior. Based on the conceptual model, a ten-dimensional scale measuring student behavior was developed and validated through mixed-method research with an exploratory sequential design. The following dimensions of student behavior were identified: 1) interaction with course content in class; 2) persistence; 3) self-learning; 4) irresponsible learning behavior; 5) active learning; 6) friendship; 7) study collaboration; 8) obedience; 9) creating a positive self-image; 10) extracurricular involvement. To develop a survey instrument, we utilize semi-structured in-depth interviews with students from eight highly selective Russian universities (n=119). Then, in the quantitative phase of the study, based on the survey (n=1,253) carried out in seven highly selective Russian universities, we tested the reliability and validity of the ten-dimensional scale. To test construct-related validity, we utilize the self-determination theory developed by Ryan and Deci and a short version of the Academic motivation scale developed by Gordeeva, Sychev, and Osin for the Russian educational context. Our findings are in line with assumptions of self-determination theory and the results of previous studies and can be considered as evidence of construct validity. The directions for further development of the methodological approach and its practical implications are discussed.

*Keywords:* student behavior at university, academic diligence, active learning, social integration, conformity, extracurricular activities

**1. Introduction**

Many researchers and practitioners in higher education have been emphasizing the crucial role of student experience at university in developing students' knowledge and skills and their professional and personal growth [1-6]. Universities worldwide have begun to consider the enhancement of student experience as one of their key strategic priorities [7, 8]. However, despite the wide academic discussion on this concept and highlighting its importance for universities’ strategic planning, student experience still has not received proper theoretical elaboration [9-12]. Attempts to define it are inconsistent and frequently limited to listing different aspects of experience related to university [4, 8, 13-15].

Many studies focused on certain aspects of student experience: student engagement [1, 16-18], extracurricular involvement [19-21], time-use [22-24], student-faculty interactions [25], student networking [26], academic misconduct and disengagement [27-29], student satisfaction [30-32], and school-to-university transition [33, 34]. However, this research obtained fragmentary knowledge about student experience because they are limited in: (1) the comparing results of various research on student experience aspects with each other, and (2) utilizing findings of this huge amount of research for improvement not only one or several aspects of the educational process and university environment at certain higher education institutions but in developing an integrated educational policy taking into account the complex combinations and interrelations between different aspects of student experience. Therefore, the use of “student experience” as an umbrella term that covers multiple separate concepts related to different aspects of university life, restricts our capacity to improve student development. It is necessary to develop a complex multidimensional concept and the methodology for its measurement which can capture not only different aspects of student experience but also interrelations between them.

The word “experience” takes the form of the noun and verb, and includes the following meanings: 1) actions of the persons that lead to certain results and changes in their personality in the form of new knowledge, skills, feelings, and ideas about the world, attitudes, etc.; 2) something that has happened and influenced a person [35, 36]. Using the phrase student experience, we limit a person’s actions and what happens to them to the time during higher education. The existing higher education studies mostly emphasize the second part of this definition: responsibility and the important role of higher education institutions in shaping and enriching this experience: teaching practices, facilities, student services, and other university conditions and opportunities, which are considered as important for student growth and can be utilized for the assessment of higher education quality [5, 17, 37, 38]. However, they mostly ignore the influence of individual characteristics that can affect relationships between the environment, experiences, and outcomes at university. Previous research identified that students' characteristics correlate with the main aspects of the student experience [39-45]. Hence, previous conceptions related to student experience are limited in explaining the mechanisms of how various aspects of the university environment and their combinations lead to certain outcomes for student development and why the experience of students from one educational program (i.e. in the same environment) can vary significantly.

We argue that not only the environmental aspects of student experience but the *behavior* of students in university settings, which is affected by the combinations and interrelations of individual and environmental characteristics, is essential for student growth. However, little attention was paid to the plurality of student behavioral patterns at university. As a rule, to examine what elements of the university environment are positive, studies focused on a single aspect of student behavior (such as participation in extracurricular activities or student misconduct) without considering it in combination with other aspects of student behavior. Such methodological approaches limit the capacity of educators to improve educational outcomes by reinforcing desired student behaviors by changing elements of the learning environment.

The present study proposes the multidimensional concept of student behavior as a crucial element of student experience and develops and validates the survey instrument for its measurement. The conceptualization can capture the diversity of behavioral patterns at university and integrate the results of empirical studies and concepts related to student experience into one conceptual model. The development of the conceptual model of student behavior was based on the synthesis of previous conceptual and empirical papers on student experience. Then, it was verified through a mixed-method empirical study. In the first phase, semi-structured interviews with 119 undergraduate students enrolled in eight highly selective Russian universities were conducted. This qualitative phase allows us to verify the proposed conceptual model and develop an instrument measuring student behavior at university for quantitative research. In the second phase, based on the survey (n=1,253) carried out in seven Russian universities (one university declined to participate further), we tested the reliability and validity of the instrument as well as the fit of the conceptual model to empirical data.

**2. Conceptualization of student behavior at university**

***2.1. Relevant concepts and theoretical frameworks***

Two competing paradigms, related to student behavior but not their cognitive processing, were developed in educational research to explain how learning occurs and how academic outcomes can be improved: behaviorism and constructivism [46]. Behaviorism focuses on the *behavior* of students as a result of environmental stimuli and consequences, and proposes that learning occurs in the process of operant conditioning [47, 48]. Constructivism emphasizes the importance of communication between students and instructors and argues that learning occurs in the process of social interactions [49]. According to behaviorism, the role of an instructor is to prepare conditions and organize stimuli (course materials, assignments, assessments, etc.) in such a way as to reinforce desired behavior and accelerate the learning process [47, 48]. While constructivism assigns an active role to students in shaping their learning experiences, setting educational goals, and achieving them through meaningful interactions at university [49-51].

Despite the concept of student behavior being close to the behavioristic paradigm in education, we suggest that ideas of constructivism are also productive for our conceptual model and can be utilized for conceiving some dimensions of student behavior, especially, aspects related to communication with instructors and other students.

There are some theoretical frameworks related to various aspects of student experience at university that can be helpful in the development of the concept of student behavior. In this section, we briefly overview these approaches and explain how they inform the theoretical assumptions of the conception of student behavior at university.

One of the most discussed concepts is student engagement. Despite student engagement being an umbrella term that covers multiple definitions and methodological approaches [17, 52], in the current research, we focus on the framework suggested by Kuh [18, 53] and McCormick [17, 54], and based on the ideas of Astin [1] and Pace [16]. This approach is of interest to our conception of student behavior for two reasons. First, it is considered as an environmental one because the effect of the learning environment on the behavior and development of the person is highlighted [55]. Second, it focuses on the observable acts of behavior at university and ignores cognitive processes which are hidden from view [52, 56]. These two features of this student engagement approach are close to the behavioristic paradigm of learning at university, which is important for our conceptualization of student behavior. However, the student engagement approach prioritizes active learning practices that are more in the tradition of constructivism. Therefore, some researchers consider this approach as constructivist [5].

The founders and supporters of the concept of student engagement argue that students acquire knowledge and skills through their activities at university, and that a university can make this process more intensive and productive by providing opportunities for participation in various in-class and out-of-class activities as well as practices, which are positively correlated with academic outcomes and student growth [53, 57, 58]. The list of these practices is based on the research of Chickering and Gamson [59], who identified “seven principles of good practices” through the analysis of empirical research on the determinants of student success. These seven principles became a starting point for the development of survey instruments measuring student engagement [17, 60]. The student engagement approach emphasizes those aspects of student behavior which are related to participation in active learning practices and can be considered as evidence of the good quality of education at university.

The other aspect of student behavior at university is related to the social connection of students to classmates and faculty. The importance of this component of student life was emphasized in the work of Tinto [61, 62], who developed a framework explaining student dropouts from university. He proposed that student dropouts are caused by a low level of academic and social integration at university. Academic integration is related to students’ academic performance, their intellectual development, and experience in academic settings, while social integration refers to social connections and the presence of positive relationships with peers [61, 63, 64]. Tinto believes that a high level of social integration can sometimes compensate for a low level of academic integration at university [65].

In addition, some researchers have identified some features of student behavior related to conformity and social desirability. Macfarlane [66] argues that the discourse of performativity resulted in such phenomena as student *presenteeism* and *learnerism*. Student presenteeism is defined as a tendency to invest a huge amount of time in learning and demonstrate hard work and diligence despite feeling unwell or exhausted and even when these efforts are not effective. Learnerism implies including in the assessment criteria characteristics of student behavior that force students to behave in certain ways: to demonstrate their interest and activity, ask questions to instructors, and to participate in class discussions. The focus shifts from meaningful contributions to class activities to demonstrating socially desired behavior to be approved by instructors.

Many researchers explore student involvement in extracurricular activities as a crucial element of student experience, which positively affects student growth and future career prospects [20, 21, 67, 68]. Although extracurricular involvement is defined as student participation in university activities separate from the primary curriculum and not related to obtaining a degree [69], this might be an effective means for student retention and adaptation to university life [21, 70]. Some researchers propose, however, that excessive extracurricular activities may have negative consequences, such as stress and anxiety due to a high workload [71]. Therefore, we suggest extracurricular activities as an important dimension of student behavior that should be considered in combination with other aspects.

The approaches presented above focus on the single aspects of student experience and behavior at university. However, there have been some attempts to construct comprehensive typologies of students based on several aspects of their experience and behavior at university. There are several typologies of students created on the basis of student time allocation between curricular and extracurricular activities at university and their life outside university [23, 24, 72]. Some researchers introduce new concepts to systematize student behavior patterns [73, 74]. For example, Clark and Trow [73] suggest the concept of student subcultures and dividing all students into groups according to their ideas, interests, attitudes to university, and relationships with other students. In a recent book, Fischman and Gardner [74] propose the concept of mental models to conceptualize student experience. This concept is used to capture student ideas about what their learning process at the university should look like, how to use additional opportunities, and how to realize themselves within university settings. Despite these conceptualizations illuminating some critical aspects of student life at university and attempting to systemize and capture differences in the student body, they have serious limitations. First, these typologies are highly sensitive to national and institutional contexts. Second, the attempts to reduce the plurality of student behavior patterns to a limited number of types leads to the oversimplification of student behavior resulting in severe restrictions of these conceptual models for measuring and explaining student experience and behavior.

To sum up, none of the existing conceptualizations can capture and explain the diversity of student behavior at the university. Concepts and theoretical approaches developed in previous research either focus on the narrow aspects of student behavior and experience at university, or oversimplify these phenomena, limiting the potential of using these approaches for research and practical implementation. The current study aims to overcome these limitations by developing a conceptual approach for the deeper understanding and measurement of diverse student behavior at university.

***2.2. Five dimensions of student behavior at university***

Following the behaviorist paradigm, we define student behavior at university as a set of stable behavioral acts occurring in response to stimuli in the university environment which vary depending on the personal characteristics of students. This definition has three important features that should be discussed in more detail. First, our conception emphasizes the role of the university and the characteristics of the program environment (faculty characteristics and course requirements, classmates, facilities) in shaping student behavior. The effect of the educational environment on student behavior is proposed by the behaviorist paradigm [47, 48] and has been demonstrated in many empirical studies [2, 3, 6].

Second, this definition highlights the diversity of students’ behavioral responses to similar stimuli from the same educational program. The variety of student behavioral responses can be explained by the differences in student personal characteristics (previous background, motivation, psychological characteristics, etc.). It has also been demonstrated in empirical research [39, 75, 76].

Third, we focus more on repeated behavioral responses because we consider them as prerequisites of student growth and academic outcomes, while single behavioral responses may have non-significant or unstable effects on the development of students. Similarly to medical literature demonstrating the importance of healthy habits and adherence to therapies for success [77, 78], we argue that regular behavior and study habits at university are crucial determinants of student growth.

Based on the review of the relevant concepts and approaches presented above, we suggested five dimensions of student behavior: 1) academic diligence; 2) active learning; 3) social integration; 4) conformity behavior; 5) extracurricular involvement. Figure 1 presents the scheme of the conceptual model, where the concept of student behavior is located at the intersection of characteristics of the university environment and the personal characteristics of students.

[Fig. 1 about here]

Academic diligence can be defined as student activities and efforts aimed at achieving educational goals at university but which cannot be directly observed: paying attention in class, investing time and effort in learning at home, doing activities to deepen knowledge, forcing themselves to study, and organizing effective course work. However, we separate these activities and efforts from the cognitive and mental processes, such as thoughts, perceptions, emotions, memorization strategies, and the peculiarities of knowledge and skill acquisition, decision making, observing the facts and behavior of others, the analysis of causal links, and making conclusions. These cognitive and mental processes are important in explaining student development and academic outcomes, but they cannot be considered as behavioral acts. Therefore, to conceptualize this dimension of student behavior, we employ the theoretical assumptions of behaviorism.

The second dimension of student behavior at university is active learning, which can be defined based on previous research on student engagement [53, 57]. We suggest that active learning is student participation in educationally purposeful practices in class which propose the observed active behavior of students and their involvement in student-faculty interactions. Active learning includes such behavioral patterns as active participation in class activities, group work, discussions, and dialogues with instructors and others. As this definition suggests the importance of student-faculty interactions, it is closer to assumptions of constructivism.

Both academic diligence and active learning are related to the curricular work of students. However, one of the main university functions is socialization. Therefore, it is important to identify behavior dimensions related to social aspects. In our conceptualization, we proposed two dimensions related to social bounds and group effects, which can occur in university settings: social integration and conformity behavior. Social integration includes the behavior of students related to building friendly relationships with other students at university and the use of these social bonds for reaching educational goals and participation in collaborative study. The social integration dimension is close to the concept of social capital [79, 80], which can be accumulated during study at university and used in university settings for achieving educational and personal goals. Previous research demonstrated that social integration is correlated with student progress and growth at university [60, 81, 82].

However, the reverse side of the social connectedness and social nature of education at university is conformity. We define conformity behavior as a change in behavior caused by the pressure of faculty and peer expectations and the desire to make a socially acceptable image of self. We hypothesize that conformity behavior is a negative phenomenon that limits the effectiveness of learning at university which has been partly demonstrated in previous studies [65, 83]. We also expect that one type of conformity behavior is associated with students' desire to meet *faculty* expectations about ‘a good student’, while another type with desire to be ‘cool’ among *peers*. The second type of conformity behavior is based on the publication dedicated to creating a positive image of themselves and peer effects in educational settings [84-88]. Thus, for example, Jackson and Dempster [84] presented the gender enactment by demonstrating differences in the learning behavior of men and women students. They found that students describe effortless achievement as ‘cool’ and ‘popular’ and associate it with masculinity, while hard work - as ‘uncool’ and associated with femininity. As students who visibly prioritize hard work are frequently labelled ‘nerd’, ‘swots’ and are often bullied, ‘cool’ some students may try to avoid hard work to maintain their status [84; 88].

Finally, we identify extracurricular activities as one of the behavioral dimensions that is in line with previous conceptual and empirical studies. This aspect is important because of its effect on academic outcomes, student growth, and well-being. The exploration of different combinations of these activities with curricular work is also productive in capturing the diverse patterns of student behavior at university.

The conceptual model is grounded in the behaviorist and constructivist paradigms explaining how learning occurs and is informed by the results of previous research on different aspects of student experience at university. The combination of different theoretical approaches with empirical evidence allows us to provide a productive methodological approach for exploring patterns of diverse student behavior at university and their combinations. To develop a survey instrument measuring student behavior at university we conducted mixed method empirical research with an exploratory sequential design.

**3. Material and methods**

***3.1. Development of the instrument***

To develop the instrument and generate items for each of the five aspects of student behavior at university, semi-structured interviews were conducted. First-year students (n=119) enrolled in eight Russian universities in 2022 took part in this phase of the research. S1 Appendix presents the procedures of the analysis of the interview data and the generation of initial items. S6 Appendix presents the final version of the developed survey instrument in the English and Russian languages. Due to the national context of the study, the initial items were formulated in Russian. The back-translation procedure was used by both authors to ensure the equivalency of the Russian and English versions. The pool of initial items included: 20 items measuring academic diligence, 11 items measuring active learning, 11 items measuring social integration, 7 items measuring conformity behavior, and 6 items measuring extracurricular involvement. For each item students were asked to indicate how often they did activities described in the item by choosing one of the following options: “never”, “rarely”, “sometimes”, “somewhat often”, or “very often”. This scale reflects our idea that by the concept of student behavior, we mean *repeated* behavioral acts. Thus, we believe that the more often a student behaves in certain ways in university settings, the more prominent a certain behavioral pattern is.

***3.2. Quantitative data***

Data for this research were collected during a longitudinal study “Educational behavior and success of university students in Russia” launched in the Fall of 2022 in seven Russian universities. This study aims to explore changes in the behavior and academic performance of students enrolled in four-year undergraduate programs in 2022 during the whole period of their study at universities. At this moment, two of four waves, which were planned in this project, have been carried out. The developed instrument measuring student behavior at university was included in the longitudinal survey as a part of the questionnaire for the second wave of the survey.

The Ethics Committee of HSE University, Russia, approved the longitudinal study “Educational behavior and success of university students in Russia” on September 19, 2022. The first wave of the study was conducted in Fall 2022. First-year students, who agreed to participate in the research project, were asked to fill out the consent form, take a standardized test on critical thinking, and to respond to questions from an online survey about their demographics, family characteristics, educational background, and school-university transition. The second wave of the study included questions about student behavior, well-being, motivation, self-reported assessment of skills improvement, time-use, and satisfaction with the educational programs and the university.

The convenience sampling method including two stages was utilized. At the first stage, we randomly selected undergraduate programs (n=131) at each university in such a way that at least one program from each field of study (mathematics and science, engineering, medicine, agriculture, social sciences, humanities, arts) was included in the research. At the second stage, each student from the selected programs was invited to participate in the research by email. The procedures of data collection were the same for all participating universities.

In the period from 11 October 2022 to 26 December 2022, 3353 first-year students participated in the study with a response rate (RR) of 66%. In the period from 26 October 2023 to 13 December 2023, 1679 of them took part in the second wave (RR = 33%). In the first wave of the longitudinal survey, each respondent was provided with an informed consent form, which was presented on the first screen of the survey. To proceed with the online questionnaire, respondents were required to click the button “To sign the form”. All respondents were of age, allowing them to sign the consent form by themselves according to Russian legislation (15 years old and more).

The current study utilizes data collected through these two waves of online surveys of students: demographics and family characteristics from the first wave and questions measuring student behavior and motivation from the second wave. For the current research, we selected students (n=1620) enrolled only in programs in four fields of study (mathematics and science, engineering, social sciences, and humanities) for two reasons. First, these fields are more common in Russian universities, and studying at these programs have fewer peculiarities related to specific professions (in contrast with medicine, agriculture, and arts). Second, we received only a few responses from students enrolled in other fields.

The following variables contained missing values: sex (0.9%, 14 missing values), family income (15.9%, 257 missing values), items measuring student behavior (up to 6.5%, 106 missing values). Totally, the 367 observations with missing data on variables used in the analysis were removed. The missing data were not replaced. The final sample included 1,253 respondents. S2 Appendix presents the descriptive statistics of the final sample. Fifty-three percent of the sample are males, 59% are from Engineering majors, 16% are from Mathematics and Science, 16% are from Humanities, and 9% are from Social sciences. Most of the sample (70%) are state-subsidized students. Sixty-four percent of the sample are from middle and high-income families.

***3.3. Data handling and statistical analyses***

Data analyses were performed using SPSS 20.0 and R. Our analytic strategy had four steps. In the first step, we ran Exploratory Factor Analysis (EFA) for each of the five sets of items developed based on the theoretical approach and interview data. The principal component analysis (PCA) was applied to identify factor structure and to decide on the number of factors measuring each aspect of student behavior. In the second step, the internal reliability of the scales was estimated by calculating Cronbach’s α. Then, in the third step, the confirmatory factor analysis (CFA) was carried out to assess the evidence based on the internal structure of the questionnaire [89]. As we employ ordinal scales in items measuring student behavior at university, the diagonally weighted least squares (DWLS) estimator was used. The model fit was evaluated based on the following fit indices: Chi-square, the comparative fit index (CFI), Tucker-Lewis index (TLI), root mean squared error of approximation (RMSEA), and the Standardized Root Mean Square Residual (SRMR). According to Schreiber et al. [90], Browne and Cudeck [91], and Hu and Bentler [92], CFI and TLI values higher than 0.90, RMSEA less than 0.06, and SRMR less than 0.08, can be considered as evidence of an acceptable fit.

Finally, to assess the evidence based on relation to other variables we tested the construct -related validity of the instrument [89]. We study the relationships between student behavior and academic motivation, which are similar constructs. We specified a structural model, in which ten factors of student behavior are dependent variables, and four factors measuring motivation are independent variables predicting student behavior at university. The following control variables was included in the proposed structural model: 1) sex (0 – male; 1 – female); 2) field of study; 3) form of financing (0 – fee-based study; 1– state-subsidized study); 4) family income group (0 – low and middle income group; 1 – high income group).

***3.4. Evidence based on the relation to other constructs***

To test construct-related validity, we developed a conceptual model of relations between motivation and student behavior, which was based on the theoretical assumptions suggesting that human behavior is driven by internal and external motives [93]. According to Deci and Ryan [94], intrinsic motivation is related to the desire to do some activities for inherent satisfaction, fun, challenge, or extending capacities (innate needs for competence), while extrinsic motives drive people to act for some separable outcomes, such as avoiding punishment or receiving an external reward. To measure motivation, the short version of the “Academic motivation scale”, which was developed and validated in the Russian educational context by Gordeeva, Sychev, and Osin [95], was employed. This instrument suggests items to measure two types of intrinsic motivation (intrinsic cognition and achievement), and two types of extrinsic motivation (introjected and external regulation). Intrinsic motivation is divided into types on the basis of the needs which students satisfy through certain behavior: cognition, achievement, or personal growth [95]. In the current research, we utilized only the two types related to cognition and achievement. The intrinsic cognition scale measures the students’ desire to learn new things and understand the subject, which is associated with experiencing interest and pleasure from the learning process itself [95]. Achievement motivation is defined as the desire to achieve high academic outcomes and to feel pleasure from the process of solving complicated problems [95].

Types of extrinsic motivation are differentiated by the degree of autonomy. Deci and Ryan [94] identified four types of extrinsic motivation: integrated, identified, introjected, and external. In the current research, we measure only two types: introjected and external, which are considered less autonomous regulations. External regulation is defined as motives that lead to the least autonomous behavior driving by the need to satisfy external demand or reward contingency [96]. Introjected regulation is more autonomous than external regulation. This suggests that individuals act in such a way to avoid guilt or anxiety and attain ego enhancements [97]. Although this behavior is internally driven, it has an external perceived locus of causality as well as external regulation. In educational contexts, it was observed that introjected regulation was positively related to investing more effort by students but, it was also related to anxiety and coping more poorly with failures [96]; externally regulated students tended to put less effort and blame others for negative outcomes [96]. Benware and Deci [98] found positive correlations between engaging in active learning, conceptual understanding, and intrinsic motivation. Kusurkar et al. [99] found that intrinsic motivation correlates with persistence, willingness to sacrifice, and readiness to start in medical students; introjected regulation has only modest significant correlations with willingness to sacrifice and readiness to start, while external regulation negatively correlates with persistence and has no significant links with willingness to sacrifice and readiness to start [99]. Maloshonok [45] found that such extracurricular activities as research work is positively correlated with motives related to achievement (developing professional skills, getting new knowledge, broadening intellectual horizons).

Based on the theoretical assumptions and the results of previous research on these types of motivation we suggest the following relationships between four types of motivation and five dimensions of student behavior. We expect that *intrinsic cognition* is positively related to academic diligence and active learning, as well as enhancing student integration and involvement in extracurricular activities. *Achievement* motivation is positively associated with academic diligence, active learning, and extracurricular involvement. In terms of external motivation, we expect that *introjected regulation* is positively linked with academic diligence and conformity behavior, while *external regulation* is negatively associated with academic diligence, active learning, conformity behavior and extracurricular involvement.

**5. Results**

***5.1. Exploratory factor analysis and internal validity***

To identify the factor structure of each aspect of student behavior, we conducted EFA.

*1. Academic diligence.* The mean values of all items ranged from 1.43 to 3.95 (standard deviation (SD): 0.80-1.23). The results of the Kaiser–Meyer–Olkin test demonstrated the sample adequacy (KMO = 0.89). The result of Barlett’s test of sphericity (χ2 (190) = 9594.32, p < 0.001) verified that the correlations between items were appropriate for factor analysis. Four factors with eigenvalues above 1.0 were extracted. Based on the analysis of items with high factor loadings, these factors were labeled: 1) interaction with course content in class; 2) persistence; 3) self-learning; 4) irresponsible learning behavior.

*2.* *Active learning.* The mean values of all items ranged from 1.94 to 3.69 (SD: 1.00-1.22). The results of the KMO test demonstrated the sample adequacy (0.90). The result of Barlett’s test (χ2 (55) = 5835.45, df = 55, p < 0.001) verified that the correlations between items were appropriate for factor analysis. Only one factor with an eigenvalue above 1.0 was extracted.

*3. Social integration.* The mean values of all items ranged from 2.30 to 4.22 (standard deviation (SD): 0.97-1.26). The results of the KMO test demonstrated the sample adequacy (KMO = 0.89). The result of Barlett’s test of sphericity (χ2 (55) = 6587.76, p < 0.001) verified that the correlations between items were appropriate for factor analysis. Two factors with eigenvalues above 1.0 were extracted. Based on the analysis of items with high factor loadings, these factors were labeled: 1) friendship; 2) study collaboration.

*4. Conformity behavior.* The mean values of all items ranged from 1.72 to 3.61 (standard deviation (SD): 1.00-1.19). The result of KMO is 0.72, which is considered a middling value but still acceptable [100]. The result of Barlett’s test of sphericity (χ2 (21) = 1630.72, p < 0.001) verified that the correlations between items were appropriate for factor analysis. Two factors with eigenvalues above 1.0 were extracted. Based on the analysis of items with high factor loadings, these factors were labeled: 1) obedience; 2) creating a positive self-image.

*5. Extracurricular involvement.* The mean values of all items ranged from 1.96 to 2.60 (SD: 1.22-1.32). The results of the KMO test demonstrated the sample adequacy (0.90). The result of Barlett’s test (χ2 (15) = 5267.20, p < 0.001) verified that the correlations between items were appropriate for factor analysis. Only one factor with an eigenvalue above 1.0 was extracted.

Based on the preliminary EFA, we identified a 10-factor structure of the initial pool of items. Then, we conducted EFA and reliability analysis for each extracted factor. The factor loadings and explained total variance are presented in Table 1. Cronbach’s alpha was used to test the internal consistency reliability of all factors extracted in EFA. Coefficients for 8 of 10 factors exceed the threshold of 0.7 [101] (see Table 1).

Table 1. Results of EFA (PCA) and reliability analysis of the ten factors of student behavior at university

| Factors | Items | Factor loadings | Total variance explained | Cronbach’s alpha |
| --- | --- | --- | --- | --- |
| 1. Academic diligence | | | | |
| 1.1. Interaction with course content in class | 4 | 0.70-0.77 | 53.53% | 0.71 |
| 1.2. Persistence | 6 | 0.58-0.72 | 46.11% | 0.76 |
| 1.3. Self-learning | 4 | 0.78-0.82 | 64.13% | 0.81 |
| 1.4. Irresponsible learning behavior | 6 | 0.64-0.77 | 50.51% | 0.80 |
| 2. Active learning | 11 | 0.57-0.76 | 44.12% | 0.87 |
| 3. Social integration | | | | |
| 3.1. Friendship | 6 | 0.66-0.76 | 52.99% | 0.82 |
| 3.2. Study collaboration | 5 | 0.68-0.80 | 54.75% | 0.80 |
| 4. Conformity behavior | | | | |
| 4.1. Obedience | 3 | 0.70-0.80 | 54.09% | 0.57 |
| 4.2. Creating a positive image of self | 4 | 0.64-0.77 | 51.89% | 0.69 |
| 5. Extracurricular involvement | 6 | 0.70-0.88 | 66.59% | 0.90 |

***5.2. Evidence based on internal structure***

Taking into account the results of EFA, in the CFA we proposed a ten-dimensional model of student behavior at university including the following factors: 1) interaction with course content in class; 2) persistence; 3) self-learning; 4) irresponsible learning behavior; 5) active learning; 6) friendship at university; 7) study collaboration; 8) obedience; 9) creating a positive self-image; 10) extracurricular involvement. The model assumed that the underlying ten factors of student behavior correlate with each other. One item measuring active learning (*“acted in a play”*) was deleted from the final model to improve model fit. The CFA of the final 54 items measuring 10 dimensions of student behavior at university demonstrated an acceptable fit to the data: χ2 (1332) = 6068.14, p < 0.001, CFI = 0.939; TLI = 0.935, RMSEA = 0.053; SRMR = 0.062. All items have significant factor loadings to their underlying constructs. Factor loadings are presented in the S3 Appendix. The correlation matrix for the 54 items of the questionnaire is presented in the S5 Appendix.

***5.3. Evidence based on the relation to other constructs***

The proposed structural model of correlation between motivation and student behavior dimensions demonstrated acceptable fit to the data: χ2 (2540) = 9326.14, p < 0.001, CFI = 0.941; TLI = 0.936, RMSEA = 0.046; SRMR = 0.055. The most structural paths supported the theoretically assumed relations between latent variables (Tables 2–4). Intrinsic cognition is positively associated with interaction with course content in class (B=0.20, p <0.05), active learning (B=0.17, p<0.01), student friendship (B=0.21, p<0.05), and study collaboration (B=0.30, p<0.001). Achievement motivation has positive correlations with persistence (B=0.21, p<0.05), self-learning (B=0.57, p<0.001), active learning (B=0.14, p<0.01), and extracurricular involvement (B=0.19, p<0.01). Introjected regulation has a strong positive link with all dimensions of academic diligence: interaction with course content in class (B=0.42, p <0.001), persistence (B=0.67, p<0.001), self-learning (B=0.29, p<0.01), and irresponsible learning behavior (B=-0.37, p<0.01). This type of external motivation is also strongly correlated with obedience (B=0.65, p<0.001). In contrast, external regulation has a negative association with interaction with course content in class (B=-0.38, p <0.01), persistence (B=-0.66, p<0.01), self-learning (B=-0.36, p<0.05), obedience (B=-0.51, p<0.01), and positive association with irresponsible learning behavior (B=0.54, p<0.001). These findings are in line with assumptions of self-determination theory and the results of previous studies [93, 96, 99], and can be considered as evidence of construct validity.

We also observed that all ten factors of student behavior correlate with each other. The covariance matrix is presented in S3 Appendix.

Table 2. Regression coefficients (B (SE)) for the model predicting academic diligence dimensions

|  | Interaction with course content in class | Persistence | Self-learning | Irresponsible  learning behavior |
| --- | --- | --- | --- | --- |
| Female | 0.05 (0.04) | 0.21*** (0.04) | -0.15** (0.05) | -0.03 (0.03) |
| Field of study (ref. – Mathematics and Science) |  |  |  |  |
| Engineering | -0.06 (0.07) | 0.05 (0.08) | 0.08 (0.09) | -0.05 (0.05) |
| Social sciences | 0.01 (0.09) | -0.03 (0.11) | -0.11 (0.12) | 0.07 (0.07) |
| Humanities | 0.07 (0.08) | -0.03 (0.10) | 0.02 (0.11) | -0.03 (0.07) |
| Motivation |  |  |  |  |
| Intrinsic cognition | 0.20* (0.10) | -0.03 (0.14) | -0.19 (0.13) | 0.03 (0.10) |
| Achievement | 0.08 (0.07) | 0.21* (0.10) | 0.57*** (0.10) | -0.08 (0.07) |
| Introjected regulation | 0.42*** (0.12) | 0.67*** (0.18) | 0.29* (0.13) | -0.37** (0.12) |
| External regulation | -0.38** (0.13) | -0.66** (0.20) | -0.36* (0.15) | 0.54*** (0.14) |
| State-subsidized | 0.07 (0.04) | -0.01 (0.05) | -0.04 (0.05) | 0.10** (0.03) |
| High family income | 0.02 (0.03) | 0.04 (0.03) | -0.01 (0.04) | -0.01 (0.02) |

Table 3. Regression coefficients for the model predicting active learning and social integration dimensions

|  | Active learning | Student friendship | Study collaboration |
| --- | --- | --- | --- |
| Female | -0.13*** (0.03) | -0.05 (0.04) | 0.03 (0.04) |
| Field of study (ref. – Mathematics and Science) |  |  |  |
| Engineering | -0.13* (0.05) | 0.07 (0.08) | 0.12 (0.08) |
| Social sciences | 0.35*** (0.08) | 0.30** (0.11) | 0.41*** (0.11) |
| Humanities | 0.25*** (0.07) | 0.17 (0.10) | 0.11 (0.10) |
| Motivation |  |  |  |
| Intrinsic cognition | 0.17** (0.06) | 0.21* (0.08) | 0.30*** (0.08) |
| Achievement | 0.14** (0.04) | 0.09 (0.06) | -0.02 (0.06) |
| Introjected regulation | 0.06 (0.05) | -0.01 (0.08) | 0.08 (0.07) |
| External regulation | -0.08 (0.06) | 0.08 (0.09) | -0.02 (0.09) |
| State-subsidized | 0.06 (0.03) | 0.01 (0.05) | 0.02 (0.05) |
| High family income | 0.05* (0.02) | 0.07* (0.03) | 0.11*** (0.03) |

Table 4. Regression coefficients for the model predicting conformity and extracurricular activities

|  | Obedience | Creating a positive image of self | Extracurricular involvement |
| --- | --- | --- | --- |
| Female | 0.01 (0.05) | -0.04 (0.05) | -0.11* (0.04) |
| Field of study (ref. – Mathematics and Science) |  |  |  |
| Engineering | 0.06 (0.09) | 0.14 (0.09) | -0.13 (0.08) |
| Social sciences | 0.20 (0.13) | 0.20 (0.12) | 0.05 (0.11) |
| Humanities | 0.23* (0.12) | 0.05 (0.11) | 0.11 (0.11) |
| Motivation |  |  |  |
| Intrinsic cognition | -0.02 (0.14) | -0.11 (0.09) | 0.01 (0.08) |
| Achievement | 0.02 (0.10) | 0.02 (0.07) | 0.19** (0.06) |
| Introjected regulation | 0.65*** (0.16) | 0.06 (0.09) | 0.04 (0.08) |
| External regulation | -0.51** (0.18) | 0.12 (0.26) | -0.05 (0.09) |
| State-subsidized | 0.04 (0.05) | 0.02 (0.05) | -0.07 (0.05) |
| High family income | 0.05 (0.04) | -0.03 (0.04) | -0.04 (0.03) |

**6. Discussion**

This study conceptualized student behavior at the university and successfully developed and validated an instrument measuring this complex construct through a ten-dimensional scale. The conceptual and measurement model is grounded in the behaviorist and constructivist paradigms explaining how learning occurs as informed by the results of previous research on different aspects of the student experience at university. The combination of different theoretical approaches with empirical evidence allow us to provide a productive methodological approach for exploring patterns of diverse student behavior at university and their combinations. It also overcomes limitations of previous theoretical approaches, which (1) suffer from insufficient theoretical elaboration, (2) propose measuring only single aspects of the student experience [19; 21; 25; 26; 27; 29 and others], or (3) oversimplify the concept of the student experience at university by constructing a comprehensive typology of students through identifying only a small number (4–6) of groups [73, 74].

In the conceptualization phase of the study, we proposed five aspects of student behavior at university, which were based on the analysis of previous research on the student experience in higher education: 1) academic diligence; 2) active learning; 3) social integration; 4) conformity behavior; 5) extracurricular activity. We assume that these aspects correlate with each other, but nevertheless can be combined by students in lots of behavioral patterns.

***6.1. Academic diligence***

Academic diligence was defined as student activities and efforts aimed at achieving educational goals at university but which cannot be directly observed. The identification of this aspect of student behavior allows us to respond to the critics of the student engagement approach about ignoring non-observable student effort and activities [52; 56]. The qualitative phase of the research provides information for generating 20 items measuring different aspects of diligence. Through the analysis of factor structure, we identified four dimensions of academic diligence: 1) interaction with course content in class; 2) persistence; 3) self-learning; and 4) irresponsible learning behavior. Interaction with course content measures student behavioral acts that take place during classes but are related to directly non-observable activities and can be differentiated from the phenomenon of active learning. Active learning suggests social interactions in class, in which an instructor and students participate in dialogues with each other, while interaction with course content suggests that the instructor delivers course material and students “receive” it by utilizing some learning strategies such as notes taking, attentive listening, relating new course material to what the student already knows. These activities should be marked as behavioral because we do not consider the results of the cognitive process and their specific features, but only the fact that students invest effort to do it.

Persistence can be defined as a type of behavior related to overcoming difficulties and organizing conditions in such a way as to achieve educational goals. It can be measured through indicators of hard work despite a loss of interest, planning own learning activities, and making efforts to make learning effective. This dimension of student behavior is close to the concept of self-regulated learning developed in the work of Zimmerman [102] and Bandura [103].

Self-learning is a behavior related to additional activities aimed to better understand course materials. It is strongly associated with achievement motivation as students can believe that their extra effort can bring higher academic outcomes. Achievement motivation is related to enjoyment from solving difficult tasks, which require more work than is required by courses. Hence, we can conclude that this correlation is in line with the theoretical assumptions of Deci and Ryan [94].

Irresponsible learning behavior is a dimension that should be considered as the opposite to academic diligence. It captures two aspects of student experience covered in previous research: disengagement, proposed by Brint and Cantwell and defined as a lack of commitment to curricular work in student’s daily activities [29, P. 810], and academic misconduct, elaborated in previous research [27, 28]. Both constructs relate to the violation of the educational process requirements but in different ways. Disengagement is inactiveness when it is needed; academic misconduct is activities, which are considered as “wrong” in academic settings. The analysis of factor structure and validity of items, measuring both phenomena but integrated in one scale, allows us to conclude that both phenomena can be considered as one latent variable when the research aim is related to the measurement of academic diligence.

According to the results of structural equation analysis, academic diligence is associated with student motivation. We found strong positive associations of academic diligence dimensions with introjected regulation, and strong negative correlations with external regulation which are in line with previous research [96, 99] and theoretical assumptions for the conceptualization of these two types of extrinsic motivation [97]. Interaction with course materials can also be driven by internal cognition motivation, while persistence and self-learning by achievement motivation. The moderate correlations between intrinsic motivation and academic diligence can be explained by the features of Russian educational context, in which curricular work in class is characterized by the high prevalence of passive learning practices and the low share of active learning practices that encourage intrinsic motivation [45; 98].

Taking into account the significance and directions of structural paths between latent variables, we conclude that the division of academic diligence into four separate dimensions of student behavior is justified and productive for capturing the variety of student behavior patterns at university and explains it through the personal characteristics of students and factors in the university environment. In general, the results of the analysis allow us to speak about the reliability and validity of the items for measuring academic diligence.

***6.2. Active learning***

Active learning is defined as student participation in educationally purposeful practices in class which propose the observed active behavior of students and their involvement in student-faculty interactions. These two features differentiate this dimension from academic diligence. Eleven items for measuring this aspect were generated based on previous research on student engagement [17, 60] and in the result of the qualitative phase of the research. The good internal consistency of these items was demonstrated based on Cronbach’s alpha coefficient and CFA. EFA demonstrated that the variance in the items can be explained by one factor. However, one item was removed from the analysis to improve the fit of the CFA model.

According to the structural model, active learning is driven by intrinsic motivation (intrinsic cognition and achievement) which is in line with the theoretical assumptions and findings of Benware and Deci [98], who demonstrated that students involved in active learning demonstrate more intrinsic motivation.

***6.3. Social integration***

According to our conceptual model, social integration includes the behavior of students related to building friendships with other students at university and the use of these social bonds for reaching educational goals and participation in collaborative study. The qualitative phase of the research provides information for generating eleven items measuring social integration. Through the analysis of factor structure, we identified two dimensions of this type of behavior: friendship and study collaboration. Friendship suggests student involvement in close relationships with classmates, which are not directly related to study. Study collaboration implies participation in dialogues and group work with classmates on topics related to course materials. The two dimensions are differentiated by questions around which the communication with classmates is built: related to the study and not related to the study. Items measuring both dimensions of social integration demonstrated good internal consistency. Both factors are correlated with intrinsic cognition. This finding is in line with previous studies dedicated to second language learners, which demonstrated the positive correlation between intrinsic motivation and willingness to communicate [104, 105]. Another study demonstrated that social integration into the peer context is a crucial prerequisite for developing intrinsic motivation [106]. As our analysis reveals the positive association between social integration and intrinsic motivation, we can consider this as evidence of construct-related validity.

***6.4. Conformity behavior***

We define conformity behavior as “the dark side” of social integration into peer and faculty contexts, and of the social nature of education at university. In our conceptual model, it is defined as a change in behavior caused by the pressure of faculty and peer expectations, and willingness to make a socially desired self-image. The identification of this behavioral dimension is supported by previous research demonstrating conformity in university students [65, 83], as well as the results of our qualitative phase, in which students admitted that they try to adjust their behavior in class to faculty expectations, creating an image of “a good student”.

In the semi-structured interviews, many students told us about their willingness to act according to faculty expectations and a fear of contradicting them (see Table 1 in S1 Appendix). However, our participants did not mention their willingness to affect their classmates' perception of them through behavioral acts. Nevertheless, we decided to develop items measuring this aspect of conformity and include them in the survey instrument. Through the analysis of factor structure, we identified two dimensions of conformity behavior: obedience and creating a positive self-image. Obedience refers to the behavior influenced by faculty expectations even if it negatively affects the learning process. Creating a positive self-image is related to behavior aimed at making a good impression on faculty and classmates. Although the CFA model demonstrates the internal consistency of these items, the Cronbach's alpha coefficient for both dimensions is close to values 0.6-0.7, which are considered as acceptable [107], but is lower than the cutoff point (0.7). Although this reliability can be considered as enough for a preliminary analysis of student conformity, comparing groups and testing correlations with other variables, further methodological work should be done to develop measurements with better reliability.

We found that obedience is positively correlated with introjected regulation and negatively with external regulation which is in line with our theoretical assumptions. However, the second conformity dimension was found not to correlate to academic motivation. Taking into account the results of our analysis, we can conclude that the conformity behavior dimension is important for a deeper understanding of the variety of behavioral patterns at university and factors influencing them. However, the types of conformity behavior and the items measuring this construct require further methodological elaboration to improve the psychometric properties of the instrument.

***6.5. Extracurricular involvement***

We define extracurricular involvement, in line with previous studies [68], as student participation in university activities that are separate from the primary curriculum and are not related to obtaining a degree. To develop items measuring this dimension, we utilized the results of the qualitative phase of the study and previous research. We identified the most widespread extracurricular activities in Russian universities and chose those that match extracurricular activities in other countries. Six items demonstrating good reliability were developed. EFA demonstrated that the variance in the items can be explained by one factor. Extracurricular involvement positively correlates with achievement motivation which is in line with our theoretical assumptions [95], previous research [45], and can be considered as evidence of construct-related validity.

**7. Limitations**

The current study has several limitations. First, the data are restricted by their convenience sampling method and the number and types of represented universities. The study was conducted in only highly selective universities, which attracted more talented and motivated students and had higher curricular requirements. As, in the qualitative phase of the study, we were restricted to first-year students from highly selective Russian universities, some specific behavioral aspects of students from non-selective universities may have been missed and not reflected in the questionnaire. Convenience sampling methods in the quantitative phase may result in self-selection bias. We assume that more academically motivated students may take part in both waves of the longitudinal survey. Second, due to the longitudinal design of the survey, we have a large number of missing values that resulting in removing some cases from the final database and in increasing the possible response bias.

Third, the Russian system of higher education, where the current study was carried out, has some specific features such as 1) a rigid curriculum and narrow specializations, 2) a high proportion of state-subsidized students who do not have to pay tuition fees for their studies, 3) the prevalence of passive learning practices in class, 4) a high curricular workload, and 5) low student involvement in extracurricular activities [45, 108]. These features can affect the results of our research: the behavioral aspects identified, items developed for their measurement, and the observed associations between academic motivation and student behavior. Therefore, the construct validity of this survey instrument needs to be evaluated and established not only in samples derived from other types of higher education institutions, but also in a wider variety of national contexts.

Finally, the study tests the construct-related validity only through the analysis of associations between student behavior dimensions and motivation. Further research should test the effect of different dimensions of student behavior on academic performance and different measures of student growth at university.

**8. Conclusions and practical implications**

We synthesized the results of various empirical research on different aspects of student experience to develop an integrated theoretical framework taking into account the multidimensional nature of student behavior at university and to develop an instrument measuring this variety of dimensions without oversimplifying the diversity of behavioral patterns at university. We also established support for the validity of a ten-dimensional instrument measuring student behavior at university, both for its entirety and for its subscales. We argue that the concept of ‘student behavior’ is a promising alternative to the concept of ‘student experience’ in higher education research because it encompasses different aspects of student life and their interrelation with each other, explains the effect of environmental and personal characteristics on student performance and growth, and has practical implications through its measurement and management.

Although the instrument requires some additional methodological work for its further validation and item revision for the conformity behavior subscale, this instrument can be utilized in research and practical goals. The results of this research may be used in further academic and institutional research and can inform educational policy. Further research is required to identify the most productive behavioral patterns for students with certain personal characteristics and intensify these patterns by creating more favorable conditions for these students at university. This methodological approach can also help identify at-risk students.

**References**

1. Astin AW. Student involvement: A developmental theory for higher education. Journal of College Student Personnel. 1984; 25(4):297–308.
2. Pascarella ET, Terenzini PT. How college affects students: Findings and insights from twenty years of research. Jossey-Bass Inc., Publishers, PO Box 44305, San Francisco, CA 94144-4305; 1991.
3. Pascarella ET, Terenzini PT. How College Affects Students: A Third Decade of Research. Volume 2. Jossey-Bass, An Imprint of Wiley. 10475 Crosspoint Blvd, Indianapolis, IN 46256; 2005.
4. Harvey L, Burrows A, Green D. Total Student Experience: A first report of the QHE national survey of staff and students' views of the important criteria for assessing the quality of higher education. Quality in Higher Education Project; 1992.
5. Coates H. The value of student engagement for higher education quality assurance. Quality in higher education. 2005 Jan 1;11(1):25-36. <https://doi.org/10.1080/13538320500074915>
6. Mayhew MJ, Rockenbach AN, Bowman NA, Seifert TA, Wolniak GC. How college affects students: 21st century evidence that higher education works. John Wiley & Sons; 2016 Sep 19.
7. Shah M, Richardson JT. Is the enhancement of student experience a strategic priority in Australian universities?. Higher Education Research & Development. 2016 Mar 3;35(2):352-64. <https://doi.org/10.1080/07294360.2015.1087385>
8. Buultjens M, Robinson P. Enhancing aspects of the higher education student experience. Journal of Higher Education Policy and Management. 2011 Aug 1;33(4):337-46. https://doi.org/10.1080/1360080X.2011.585708
9. Jones JR. So just what is the student experience? The development of a conceptual framework for the student experience of undergraduate business students based on the themes emerging in the academic literature. InSRHE Annual Research Conference 2010. Newcastle University.
10. Jones R. The student experience of undergraduate students: towards a conceptual framework. Journal of Further and Higher Education. 2018 Nov 17;42(8):1040-54. <https://doi.org/10.1080/0309877X.2017.1349882>
11. Sabri D. What's wrong with ‘the student experience’?. Discourse: Studies in the cultural politics of education. 2011 Dec 1;32(5):657-67. ttps://[doi.org/10.1080/01596306.2011.620750](http://doi.org/10.1080/01596306.2011.620750)
12. Pötschulat M, Moran M, Jones P. ‘The student experience’ and the remaking of contemporary studenthood: A critical intervention. The Sociological Review. 2021 Jan;69(1):3-20. <https://doi.org/10.1177/0038026120946677>
13. Crossing S. The student experience: How can universities meet student demands? Studiosity Blog. 2018 Feb 15 [Cited 2024 May 29]. Available from: https://www.studiosity.com/blog/the-student-experience-how-can-universities-meet-student-demands.
14. Benckendorff P, Ruhanen L, Scott N. Deconstructing the student experience: A conceptual framework. Journal of Hospitality and Tourism Management. 2009 Jan 1;16(1):84-93. <https://doi.org/10.1375/jhtm.16.1.84>
15. Amado Mateus M, Guzmán Rincón A, Cuero Acosta YA. Student experience scale: Factor analysis in higher education institutions. In Frontiers in Education 2023 Jan 4 (Vol. 7, p. 1057651). Frontiers. <https://doi.org/10.3389/feduc.2022.1057651>
16. Pace CR. Measuring the quality of student effort. Current issues in higher education. 1980;2(1):10-6.
17. McCormick AC, Kinzie J, Gonyea RM. Student engagement: Bridging research and practice to improve the quality of undergraduate education. InHigher Education: Handbook of Theory and Research: Volume 28 2013 Jan 4 (pp. 47-92). Dordrecht: Springer Netherlands.
18. Kuh GD. The national survey of student engagement: Conceptual and empirical foundations. New directions for institutional research. 2009;141:5-20. <https://doi.org/10.1002/ir>
19. Hu S, Kuh GD, Gayles JG. Engaging undergraduate students in research activities: Are research universities doing a better job?. Innovative Higher Education. 2007 Oct;32:167-77. <https://doi.org/10.1007/s10755-007-9043-y>
20. Roulin N, Bangerter A. Students’ use of extra-curricular activities for positional advantage in competitive job markets. Journal of Education and Work. 2013 Feb 1;26(1):21-47. <https://doi.org/10.1080/13639080.2011.623122>
21. Buckley P, Lee P. The impact of extra-curricular activity on the student experience. Active Learning in Higher Education. 2021 Mar;22(1):37-48. <https://doi.org/10.1177/1469787418808988>
22. Marshall SJ. Student time choices and success. Higher Education Research & Development. 2018 Sep 19;37(6):1216-30. <https://doi.org/10.1080/07294360.2018.1462304>
23. Fosnacht K, McCormick AC, Lerma R. First-year students’ time use in college: A latent profile analysis. Research in Higher Education. 2018 Nov;59(7):958-78. <https://doi.org/10.1007/s11162-018-9497-z>
24. Maloshonok N. Undergraduate time-use: a comparison of US, Chinese, and Russian students at highly selective universities. Higher Education Research & Development. 2020 Apr 15;39(3):515-31. <https://doi.org/10.1080/07294360.2019.168445>
25. Kuh GD, Hu S. The effects of student-faculty interaction in the 1990s. The review of higher education. 2001;24(3):309-32. <https://doi.org/10.1353/rhe.2001.0005>
26. Vignery K. Findings and methodologies about student networks, learning, performance and academic achievement in higher education: A literature review on quantitative studies. Review of Education. 2021 Oct;9(3):e3308. <https://doi.org/10.1002/rev3.3308>
27. McCabe DL, Trevino LK, Butterfield KD. Academic integrity in honor code and non-honor code environments: A qualitative investigation. The Journal of Higher Education. 1999 Mar 1;70(2):211-34. <https://doi.org/10.1080/00221546.1999.11780762>
28. Denisova-Schmidt E. The challenges of academic integrity in higher education: Current trends and prospects. CIHE perspectives. 2017;5:1-27.
29. Brint S, Cantwell AM. Conceptualizing, measuring, and analyzing the characteristics of academically disengaged students: Results from UCUES 2010. Journal of College Student Development. 2014;55(8):808-23. [https://doi.org/](https://doi.org/10.1080/00221546.1999.11780762)10.1353/csd.2014.0080
30. Elliott, K.M. and Shin, D., 2002. Student satisfaction: An alternative approach to assessing this important concept. Journal of Higher Education policy and management, 24(2), pp.197-209. <https://doi.org/10.1080/1360080022000013518>
31. Douglas J, McClelland R, Davies J. The development of a conceptual model of student satisfaction with their experience in higher education. Quality assurance in education. 2008 Feb 1;16(1):19-35. [https://doi.org/](https://doi.org/10.1080/1360080022000013518)10.1108/09684880810848396
32. Schertzer CB, Schertzer SM. Student satisfaction and retention: A conceptual model. Journal of Marketing for Higher Education. 2004 Nov 8;14(1):79-91. <https://doi.org/10.1300/J050v14n01_05>
33. Bryde JF, Milburn CM. Helping to make the transition from high school to college. Understanding undergraduate education. 1990:203-13.
34. Brinkworth R, McCann B, Matthews C, Nordström K. First year expectations and experiences: Student and teacher perspectives. Higher Education. 2009 Aug;58:157-73. <https://doi.org/10.1007/s10734-008-9188-3>
35. Cambridge University Press. Experience. Cambridge dictionary. n.d. [May 2024 29]. Available from: from https://dictionary.cambridge.org/dictionary/english/experience.
36. Collins English Dictionary.. Experience. Collins English dictionary. n.d. [May 2024 29]. Available from: https://www.collinsdictionary.com/dictionary/english/experience.
37. Pascarella ET. identifying excellence in undergraduate education are we even close?. Change: The Magazine of Higher Learning. 2001 May 1;33(3):18-23. <https://doi.org/10.1080/00091380109601796>
38. Zeng LM, Fryer LK, Zhao Y. A comparison of three major instruments used for the assessment of university student experience: Toward a comprehensive and distributed approach. Higher Education Quarterly. 2023 Jan;77(1):27-44. <https://doi.org/10.1111/hequ.12363>
39. Kinzie J, Gonyea R, Kuh GD, Umbach P, Blaich C, Korkmaz A. The relationship between gender and student engagement in college. Association for the Study of Higher Education Annual Conference.
40. Saenz VB, Hatch D, Bukoski BE, Kim S, Lee KH, Valdez P. Community college student engagement patterns: A typology revealed through exploratory cluster analysis. Community College Review. 2011 Jul;39(3):235-67. <https://doi.org/10.1177/0091552111416643>
41. Sontam V, Gabriel G. Student engagement at a large suburban community college: Gender and race differences. Community College Journal of Research and Practice. 2012 Oct 1;36(10):808-20. <https://doi.org/10.1080/10668926.2010.491998>
42. Hedrick B, Dizen M, Collins K, Evans J, Grayson T. Perceptions of College Students with and without Disabilities and Effects of STEM and Non-STEM Enrollment on Student Engagement and Institutional Involvement. Journal of Postsecondary Education and Disability. 2010;23(2):129-36.
43. Hu S, Kuh GD. Being (dis) engaged in educationally purposeful activities: The influences of student and institutional characteristics. Research in higher education. 2002 Oct;43:555-75. <https://doi.org/10.1023/A:1020114231387>
44. Chi X, Liu J, Bai Y. College environment, student involvement, and intellectual development: evidence in China. Higher Education. 2017 Jul;74:81-99. <https://doi.org/10.1007/s10734-016-0030-z>
45. Maloshonok N. Do student engagement patterns differ across national higher education systems? The comparison of US, Chinese, and Russian high-level research-intensive universities. Innovations in Education and Teaching International. 2023 Feb 26:1-2. <https://doi.org/10.1080/14703297.2023.2183883>
46. Hinduja P. From behaviorism to constructivism in teaching-learning process. Journal of Education & Social Sciences. 2021 Dec 22;9(2):111-22. [https://doi.org/](https://doi.org/10.1177/016146816306500209)10.20547/jess0922109204
47. Skinner BF. Reflections on a decade of teaching machines. Teachers College Record. 1963 Nov;65(2):1-9. <https://doi.org/10.1177/016146816306500209>
48. Skinner BF. Review Lecture-The technology of teaching. Proceedings of the Royal Society of London. Series B. Biological Sciences. 1965 Jul 27;162(989):427-43. <https://doi.org/10.1098/rspb.1965.0048>
49. Wulf C. “From teaching to learning”: Characteristics and challenges of a student-centered learning culture. Inquiry-based learning–Undergraduate research: The German multidisciplinary experience. 2019:47-55.
50. Bramming P. An argument for strong learning in higher education. Quality in Higher Education. 2007 Apr 1;13(1):45-56. <https://doi.org/10.1080/13538320701272722>
51. De la Sablonnière R, Taylor DM, Sadykova N. Challenges of applying a student-centered approach to learning in the context of education in Kyrgyzstan. International Journal of Educational Development. 2009 Nov 1;29(6):628-34. <https://doi.org/10.1016/j.ijedudev.2009.01.001>
52. Kahu ER. Framing student engagement in higher education. Studies in higher education. 2013 Jun 1;38(5):758-73. <https://doi.org/10.1080/03075079.2011.598505>
53. Kuh GD. What we're learning about student engagement from NSSE: Benchmarks for effective educational practices. Change: The magazine of higher learning. 2003 Mar 1;35(2):24-32. <https://doi.org/10.1080/00091380309604090>
54. McCormick AC. Toward reflective accountability: Using NSSE for accountability and transparency. New Directions for Institutional Research. 2009 Mar;2009(141):97-106. <https://doi.org/10.1002/ir.289>
55. Long D. Theories and models of student development. In: Hinchliffe LJ, Wong MA, editors. Environments for student growth and development: Librarians and student affairs in collaboration. Chicago: Association of College & Research Libraries; 2012. pp. 41-55.
56. Kahn P. Higher education policy on student engagement: Thinking outside the box. Higher Education Policy. 2017 Mar;30:53-68. <https://doi.org/10.1057/s41307-016-0031-7>
57. Kuh GD, Kinzie JL, Buckley JA, Bridges BK, Hayek JC. What matters to student success: A review of the literature. Washington, DC: National Postsecondary Education Cooperative; 2006 Jul.
58. Krause KL, Coates H. Students’ engagement in first‐year university. Assessment & Evaluation in Higher Education. 2008 Oct 1;33(5):493-505. <https://doi.org/10.1080/02602930701698892>
59. Chickering AW, Gamson ZF. Seven principles for good practice in undergraduate education. AAHE bulletin. 1987 Mar;3:7.
60. Kuh GD, Pace CR, Vesper N. The development of process indicators to estimate student gains associated with good practices in undergraduate education. Research in higher education. 1997 Aug;38:435-54. <https://doi.org/10.1023/A:1024962526492>
61. Tinto V. Leaving college: Rethinking the causes and cures of student attrition. University of Chicago press; 2012 Apr 27.
62. Tinto V. Classrooms as communities: Exploring the educational character of student persistence. The Journal of higher education. 1997 Nov 1;68(6):599-623. <https://doi.org/10.1080/00221546.1997.11779003>
63. Tinto V. Dropout from higher education: A theoretical synthesis of recent research. Review of educational research. 1975 Mar;45(1):89-125. <https://doi.org/10.3102/00346543045001089>
64. Lakhal S, Mukamurera J, Bédard ME, Heilporn G, Chauret M. Features fostering academic and social integration in blended synchronous courses in graduate programs. International Journal of Educational Technology in Higher Education. 2020 Dec;17:1-22. <https://doi.org/10.1186/s41239-020-0180-z>
65. Pascarella ET, Terenzini PT. Interaction effects in Spady and Tinto's conceptual models of college attrition. Sociology of education. 1979 Oct 1:197-210. <https://doi.org/10.2307/2112401>
66. Macfarlane B. Student performativity in higher education: Converting learning as a private space into a public performance. Higher Education Research & Development. 2015 Mar 4;34(2):338-50. <https://doi.org/10.1080/07294360.2014.956697>
67. Loes CN, Pascarella ET. Collaborative learning and critical thinking: Testing the link. The Journal of Higher Education. 2017 Sep 3;88(5):726-53. <https://doi.org/10.1080/00221546.2017.1291257>
68. Finnerty R, Marshall SA, Imbault C, Trainor LJ. Extra-curricular activities and well-being: Results from a survey of undergraduate university students during COVID-19 lockdown restrictions. Frontiers in Psychology. 2021 Jun 28;12:647402. <https://doi.org/10.3389/fpsyg.2021.647402>
69. Munir S, Zaheer M. The role of extra-curricular activities in increasing student engagement. Asian Association of Open Universities Journal. 2021 Dec 14;16(3):241-54. https://doi.org/10.1108/AAOUJ-08-2021-0080
70. Bronkema RH, Bowman NA. Close campus friendships and college student success. Journal of College Student Retention: Research, Theory & Practice. 2019 Nov;21(3):270-85. <https://doi.org/10.1177/1521025117704200>
71. Ribeiro N, Malafaia C, Neves T, Menezes I. The impact of extracurricular activities on university students’ academic success and employability. European Journal of Higher Education. 2023 May 5:1-21. <https://doi.org/10.1080/21568235.2023.2202874>
72. Hensley LC, Wolters CA, Won S, Brady AC. Academic probation, time management, and time use in a college success course. Journal of College Reading and Learning. 2018 Feb 23;48(2):105-23. <https://doi.org/10.1080/10790195.2017.1411214>
73. Clark BR. Determinants of college student subculture. Center for the Study of Higher Education, University of California (Berkeley); 1960.
74. Fischman W, Gardner H. The real world of college: What higher education is and what it can be. MIT Press; 2022 Mar 22.
75. Hu S, Wolniak GC. College student engagement and early career earnings: Differences by gender, race/ethnicity, and academic preparation. The Review of Higher Education. 2013;36(2):211-33. <https://doi.org/10.1353/rhe.2013.0002>
76. Kuh GD, Umbach P. College and character: Insights from the national survey of student engagement. New Directions for Institutional Research. 2004; 122: 37-54.
77. Arlinghaus KR, Johnston CA. The importance of creating habits and routine. American journal of lifestyle medicine. 2019 Mar;13(2):142-4. <https://doi.org/10.1177/1559827618818044>
78. Sabaté E, editor. Adherence to long-term therapies: evidence for action. World Health Organization; 2003.
79. Bourdieu P. The forms of capital. In: Richardson JG, editor. Handbook of theory and research for the sociology of education. Westoort. CT: GreenwoodPress; 1986. pp. 241-258.
80. Martin ND, Spenner KI. Capital conversion and accumulation: A social portrait of legacies at an elite university. Research in Higher Education. 2009 Nov;50:623-48. <https://doi.org/10.1007/s11162-009-9136-9>
81. Severiens SE, Schmidt HG. Academic and social integration and study progress in problem based learning. Higher Education. 2009 Jul;58(1):59-69. <https://doi.org/10.1007/s10734-008-9181-x>
82. Bers TH, Smith KE. Persistence of community college students: The influence of student intent and academic and social integration. Research in higher Education. 1991 Oct;32:539-56. <https://doi.org/10.1007/BF00992627>
83. Marrs H. Conformity to masculine norms and academic engagement in college men. Psychology of Men & Masculinity. 2016 Apr;17(2):197.. <https://doi.org/10.1037/men0000015>
84. Jackson C, Dempster S. ‘I sat back on my computer… with a bottle of whisky next to me’: Constructing ‘cool’masculinity through ‘effortless’ achievement in secondary and higher education. Journal of Gender Studies. 2009 Dec 1;18(4):341-56. <https://doi.org/10.1080/09589230903260019>
85. Jackson C, Nyström AS. ‘Smart students get perfect scores in tests without studying much’: Why is an effortless achiever identity attractive, and for whom is it possible?. Research Papers in Education. 2015 Aug 8;30(4):393-410. <https://doi.org/10.1080/02671522.2014.970226>
86. Heyder A, Kessels U. Boys don’t work? On the psychological benefits of showing low effort in high school. Sex Roles. 2017 Jul;77:72-85. <https://doi.org/10.1007/s11199-016-0683-1>
87. Foster G. It's not your peers, and it's not your friends: Some progress toward understanding the educational peer effect mechanism. Journal of public Economics. 2006 Sep 1;90(8-9):1455-75.
88. Jackson D. Geeks, boffins, swots and nerds: a social constructionist analysis of ‘gifted and talented’identities in post-16 education (Doctoral dissertation, UCL Institute of Education).
89. Goodwin LD, Leech NL. The meaning of validity in the new standards for educational and psychological testing: Implications for measurement courses. Measurement and evaluation in Counseling and Development. 2003 Oct 1;36(3):181-91. <https://doi.org/10.1080/07481756.2003.11909741>
90. Schreiber JB, Nora A, Stage FK, Barlow EA, King J. Reporting structural equation modeling and confirmatory factor analysis results: A review. The Journal of educational research. 2006 Jul 1;99(6):323-38. <https://doi.org/10.3200/JOER.99.6.323-338>
91. Browne MW, Cudeck R. Alternative ways of assessing model fit. Sociological methods & research. 1992 Nov;21(2):230-58. <https://doi.org/10.1177/0049124192021002005>
92. Hu LT, Bentler PM. Cutoff criteria for fit indexes in covariance structure analysis: Conventional criteria versus new alternatives. Structural equation modeling: a multidisciplinary journal. 1999 Jan 1;6(1):1-55.
93. Ryan RM, Deci EL. Intrinsic and extrinsic motivations: Classic definitions and new directions. Contemporary educational psychology. 2000 Jan 1;25(1):54-67. <https://doi.org/10.1006/ceps.1999.1020>
94. Deci EL, Ryan RM. The" what" and" why" of goal pursuits: Human needs and the self-determination of behavior. Psychological inquiry. 2000 Oct 1;11(4):227-68. <https://doi.org/10.1207/S15327965PLI1104_01>
95. Gordeeva TO, Sychev OA., Osin EN. Academic motivations scales questionnaire. Psychological Journal. 2014; 35: 96-107.
96. Ryan RM, Deci EL. Self-determination theory and the facilitation of intrinsic motivation, social development, and well-being. American psychologist. 2000 Jan;55(1):68. <https://doi.org/10.1037/0003-066X.55.1.68>
97. Deci EL, Ryan RM. Human autonomy: The basis for true self-esteem. InEfficacy, agency, and self-esteem 1995 Jul 3 (pp. 31-49). Boston, MA: Springer US.
98. Benware CA, Deci EL. Quality of learning with an active versus passive motivational set. American educational research journal. 1984;21(4):755-65. <https://doi.org/10.3102/00028312021004755>
99. Kusurkar R, Croiset G, Kruitwagen C, ten Cate O. Validity evidence for the measurement of the strength of motivation for medical school. Advances in health sciences education. 2011 May;16:183-95. <https://doi.org/10.1007/s10459-010-9253-4>
100. Kaiser HF, Rice J. Little jiffy, mark IV. Educational and psychological measurement. 1974 Apr;34(1):111-7. <https://doi.org/10.1177/001316447403400115>
101. Lance CE, Butts MM, Michels LC. The sources of four commonly reported cutoff criteria: What did they really say?. Organizational research methods. 2006 Apr;9(2):202-20. <https://doi.org/10.1177/1094428105284919>
102. Zimmerman BJ. Self-regulated learning and academic achievement: An overview. Educational psychologist. 1990 Jan 1;25(1):3-17. <https://doi.org/10.1207/s15326985ep2501_2>
103. Bandura A. The evolution of social cognitive theory. Great minds in management. 2005 Sep 22:9-35.
104. Joe HK, Hiver P, Al-Hoorie AH. Classroom social climate, self-determined motivation, willingness to communicate, and achievement: A study of structural relationships in instructed second language settings. Learning and individual differences. 2017 Jan 1;53:133-44. <https://doi.org/10.1016/j.lindif.2016.11.005>
105. Peng JE, Woodrow L. Willingness to communicate in English: A model in the Chinese EFL classroom context. Language learning. 2010 Dec;60(4):834-76. <https://doi.org/10.1111/j.1467-9922.2010.00576.x>
106. Reindl M, Auer T, Gniewosz B. Social integration in higher education and development of intrinsic motivation: A latent transition analysis. Frontiers in psychology. 2022 Jun 14;13:877072. <https://doi.org/10.3389/fpsyg.2022.877072>
107. Cronbach LJ. Coefficient alpha and the internal structure of tests. psychometrika. 1951 Sep;16(3):297-334. <https://doi.org/10.1007/BF02310555>
108. Kuzminov Ya.I, Yudkevich MM. Russian Universities: How the System Works. Moscow: HSE Publishing House; 2021.
