# Supplementary material for: Student behavior at university: The development and validation of a 10-dimensional scale
Source: PLoS One. 2024 Nov 15;19(11):e0313357. doi: 10.1371/journal.pone.0313357 (PMC11567547; doi:10.1371/journal.pone.0313357)
Supplement: Attachment — Submitted filename: Response to Reviewers.docx [file pone.0313357.s009.docx]

Response to Reviewers

| Reviewers’ comments | Author’s responses |
| --- | --- |
| Academic editor |  |
| The article was reviewed by 2 different reviewers and the opinions are not uniform.  I would like to pay attention to reviewer 1's comments, they are detailed and refer to the length of the article and its various sections. Pay special attention to the comments regarding the findings. | Dear Dr. Gal Harpaz,  Thank you for your positive response and comments to the paper. We revised the manuscript to make it more accessible and useful for readers. We removed repetitive information and revised the structure of some sections, added headings and more explanations about the structure and value of different sections for achieving the aim of the paper. Moreover, we revised figure 1 and supporting information files to make them more clear, and proofread the text using the AJE editing service. |
| Reviewer 1 | Dear Dr Glenda Crosling,  Thank you so much for your thorough reading and helpful comments on this manuscript. We greatly appreciate your help in improving the quality of the manuscript. The detailed answers to your comments and description of changes in the text are presented below. |
| The paper is very well researched, and the model presented well supported by the literature from which it is drawn. However, the paper is very long in word length, and even more so when the information in the six appendices is included. The paper is quite broad in its coverage, and while well expressed, is deep. The author/s need to refine the paper including the appendices to streamline it and render it more accessible to readers. | Thank you for the comment. We revised the manuscript to make it more accessible and useful for readers. We removed repetitive information, revised the structure of some sections, added headings and more explanations about the structure and value of different sections for achieving the aim of the paper. We also revised appendices to make it more clear, and proofread the text using the AJE editing service. |
| There are some discrepancies in the paper that require review. For instance, it is stated earlier in the paper that eight universities participated in the study, but in Section 3.2 this number is stated as seven. | Thank you for your comment. To develop the instrument we employed mixed-method research with an exploratory sequential design. In the first stage, we conducted in-depth interviews with students from *eight* universities. In the second stage, we carried out a survey at *seven* universities. One university that participated in the first stage did not participate in the second stage. This information is correct. We tried to make it more clear by adding the following text highlighted in green: Data for this research were collected during a longitudinal study “Data for this research were collected during a longitudinal study titled “*Educational behavior and success of university students in Russia” launched in the fall of 2022 in seven Russian universities (one of the eight universities participating in the qualitative phase did not participate in the survey).*  In the previous version of the manuscript, this information was mentioned in the Introduction: *“In the second phase, based on the survey (n=1,253) carried out in seven Russian universities (one university declined to participate further), we tested the reliability and validity of the instrument as well as the fit of the conceptual model to empirical data”*. However, we removed this text to avoid repetition of the information. |
| The concept of ‘highly selective’ universities in Russia needs definition as it may differ across the countries of the journal’s readership. | We added the following explanation in the Materials and Method Section: *Highly selective universities in Russia represent the group of higher education institutions that have the highest chances of attracting the most talented students. To be admitted to university, Russian applicants must pass the Unified State Exam (USE). Universities select applicants based on their USE scores. The applicants with the highest scores can be admitted to the most prestigious universities, and the quality of admission at Russian universities is estimated by the average USE scores of admitted students. We consider universities in the highest quartile by the average USE score of admitted students to be highly selective. Therefore, our participants, who are represented by students from highly selective universities, may be more talented and motivated to study at university than the general population of Russian students.* |
| A brief explanation of the notion of ‘convenience sampling’ would also be useful for readers. | Thank you for the comment. We added the following clarification: *Convenience sampling is a nonrandom sampling technique involving respondents who are “convenient” to a researcher (for example, recruiting people in the street, in a workplace, or on the internet). In our research, we recruited students who used email services, opened emails with invitations and were willing to participate in the survey. The recruiting students through emails and the volunteer nature of their participation can lead to bias in the data discussed in the Limitations section.* |
| The paper needs to be reviewed carefully so that there is not repetition of information across it. For instance, some information in the Methods section has been presented earlier in the paper. | Thank you for the comment. We reviewed the paper and removed repetition.   - we removed information about the empirical research from the Introduction section, because it is presented in detail in the Materials and methods section - we removed repetitive information from 3.2. Quantitative data section |
| The abstract mentions self determination theory, but this does not seem to be discussed in the paper. | In the abstract, we mentioned that we used self-determination theory to test construct-related validity. Therefore, we present this theory in section ***3.4.*** ***Evidence based on the relation to other constructs.*** In our opinion, it is a better decision to present this theory in the section where we use its assumptions to test construct-related validity, rather than in the Literature review sections, which are aimed to presenting the concept of student behavior. |
| Further development of the diagram in Figure 1 would enhance the paper, so that the diagram includes the research areas from which the five behavioural indicators emanate. | We revised Figure 1 to make it more informative. We added the definitions of each dimension and highlighted their differences. |
| The results section should include comment on the value of the sections of the model in relation to the purpose of the paper, which is to examine its validity in measuring students’ behaviour. | Thank you for the comment. We added the following text to present the value of each section for the purpose:  *In this section, we demonstrate the psychometric properties of the instrument for measuring student behavior. First, we identify the factor structure of each aspect of student behavior that was assumed based on the literature review and quantitative phase of the research. This analysis allows us to identify a certain number of student behavior dimensions. Second, to demonstrate the reliability of the instrument, we investigate the internal structure of the dimensions and their interrelations with each other. Finally, to test the construct validity of the instrument, we explore the relationships between student behavior dimensions and other constructs. For this purpose, we utilize the self-determination theory developed by Ryan and Deci [95] and a short version of the Academic Motivation Scale developed by Gordeeva, Sychev, and Osin [96] for the Russian educational context. The observed correlations between student behavior dimensions and different types of motivation that are in line with the assumptions of self-determination theory are evidence of the construct validity of the instrument.* |
| The conclusion in the paper is brief and should be expanded to discuss the aim of the model developed as presented on page 4 (line 64), which is to improve students’ development. | Thank you for the comment. We expanded the conclusion and practical implication section to explain how the developed conceptual model and instrument measuring student behavior can be utilized for enhancing student development at university. |
| I am unclear if the end note referencing system used in the paper is the style required by the journal.  The validation of the statistical analysis undertaken in the study has also not been undertaken in this review. | We have checked the Journal requirements. According to the Journal rules, footnotes are not permitted. The Journal advices to move the information into the main text or the reference list, depending on the content. |
| In all, the paper is well researched, and the model developed logical and well supported. The paper is well structured and clearly expressed. However, my reading is that it is a complex and overly long paper which requires further work to refine and streamline it. I wish the author/s well as they proceed further with the paper. | We greatly appreciate your positive feedback and comments! We did our best to make this paper more clear and accessible for readers. |
| Reviewer 2 | Dear Exelis Moise Pierre,  Thank you so much for your thorough reading, useful comments and help with editing the manuscript. We very appreciate your help in improving the text! |
| I have provided some review tracks & changes from the original MS word manuscript to help authors in the English language standard | Thank you very much! We very appreciate your help with editing the manuscript. We accept all suggested changes. Also, we proofread the revised version of the manuscript using the AJE editing service. |
| Authors should provide the R studio year complete citation and put in reference the associated system full details. | Thank you for the comment. We provided the R studio complete citation and put it on the reference list. Also, we put more details about the R Studio version. |
| These are technical expression associated specifically to this field of study? (*to the sentence: This instrument suggests items to measure two types of intrinsic motivation (intrinsic cognition and achievement), and two types of extrinsic motivation (introjected and external regulation)*) | Introjected and external regulation are two types of extrinsic motivation that were proposed by Deci and Ryan. We utilize the assumptions of their self-determination theory and their terminology. |
| Important point given. A student would artificially modify his/her natural behavior according to a faculty made standards. Unless the inner behavior of the students were not associated with crimes, social type of unrests and the like – this could adversely affect in the long term the comfort of a student. Others studies proved that respecting somehow the diversity atmosphere among students if this is related to cultural factors, is actually a great asset for any institution of higher learning. It is even considered as a potential enrichment being shared in the community. It is considered as a betterment in the future and worthwhile as an enhancement and motivational pattern in students. They just feel at home. Many negative factors are totally erased by implementing such policy i.e. stress, homesick, cultural shock, difficulty of adaptation and the like. This point alone can be made as the aim of a whole study.  I do suggest to the authors to relate this briefly and show its necessary association to obtain a conducive and better faculty or university itself environment. This will affect the whole relationship model inside the university, faculties, departments. We understand that there are multicultural ethnic groups in Russia. Hence this point can be briefly emphasized further. The psychological impact of giving attention to such cultural diversity without trying to frame the students into a single and uniform model is very productive. The student are just happy. The opposite may create stressful atmosphere and the sentiment of rejection in the long term. | Thank you so much for mentioning this. We agree that it is a very important research topic that should be explored in further research, and this is an aim for the whole study. We briefly relate this point by adding the following text:  *“This dimension may have greater importance in cultures that assume high power distance between teachers and students and promote student compliance and teacher authority, as well as in classes with international students or ethnic minorities from those cultures.”.* |
| Need to find another expression to show the concept that is being analyzed. (to introjected regulation) | Thank you for your comment. The term “introjected regulation” is one type of extrinsic motivation proposed by Deci and Ryan and measured by the short version of the instrument “Academic motivation scale”. In section 3.4., we give the following definition of this type of motivation: “Introjected regulation is more autonomous than external regulation. This suggests that individuals act in such a way to avoid guilt or anxiety and attain ego enhancements [97].” |
| Journal Requirements: |  |
| 1. Please ensure that your manuscript meets PLOS ONE's style requirements, including those for file naming. The PLOS ONE style templates can be found at  https://journals.plos.org/plosone/s/file?id=wjVg/PLOSOne_formatting_sample_main_body.pdf and  https://journals.plos.org/plosone/s/file?id=ba62/PLOSOne_formatting_sample_title_authors_affiliations.pdf | Thank you. We revised the manuscript according to PLOS ONE's style requirements. |
| 2. Please provide additional details regarding participant consent. In the ethics statement in the Methods and online submission information, please ensure that you have specified what type you obtained (for instance, written or verbal, and if verbal, how it was documented and witnessed). If your study included minors, state whether you obtained consent from parents or guardians. If the need for consent was waived by the ethics committee, please include this information. | In the previous version of the submission we provided detailed information about the participant consent and ethics of this research. We have attached the ethics certificate in the Russian language and its translation, also we clarify some peculiarities of this document caused by the consideration procedure adopted at the Ethics Committee of HSE University.  The text in the manuscript regarding participant consent:  *“Each respondent was provided with an informed consent form, which was presented on the first screen of the online survey. To proceed with the online questionnaire, the respondents were required to click the button “To sign the form”. All respondents were of age, allowing them to sign the consent form by themselves according to Russian legislation (15 years or older)”.* |
| 3. We note that the grant information you provided in the ‘Funding Information’ and ‘Financial Disclosure’ sections do not match.  When you resubmit, please ensure that you provide the correct grant numbers for the awards you received for your study in the ‘Funding Information’ section. | Thank you for the comment.  We added to the manuscript the following information about funding:  “**Funding:** Support from the Basic Research Program of the National Research University Higher School of Economics (HSE University) is gratefully acknowledged. The funder had no role in study design, data collection and analysis, decision to publish, or preparation of the manuscript.” |
| 4. Thank you for stating the following financial disclosure:  "the Basic Research Program of the National Research University Higher School of Economics (HSE University)"  Please state what role the funders took in the study. If the funders had no role, please state: "The funders had no role in study design, data collection and analysis, decision to publish, or preparation of the manuscript."  If this statement is not correct you must amend it as needed.  Please include this amended Role of Funder statement in your cover letter; we will change the online submission form on your behalf. | Thank you for the comment. According to Journal Requirements, we would like to ask you to add the following information about the Role of Funder: The funder had no role in study design, data collection and analysis, decision to publish, or preparation of the manuscript. |
| 5. Thank you for stating the following in the Acknowledgments Section of your manuscript:  "Support from the Basic Research Program of the National Research University Higher School of Economics (HSE University) is gratefully acknowledged."  We note that you have provided funding information that is not currently declared in your Funding Statement. However, funding information should not appear in the Acknowledgments section or other areas of your manuscript. We will only publish funding information present in the Funding Statement section of the online submission form.  Please remove any funding-related text from the manuscript and let us know how you would like to update your Funding Statement. Currently, your Funding Statement reads as follows:  "the Basic Research Program of the National Research University Higher School of Economics (HSE University)"  Please include your amended statements within your cover letter; we will change the online submission form on your behalf. | Thank you. We would like to update our funding statement in the following ways:  Support from the Basic Research Program of the National Research University Higher School of Economics (HSE University) is gratefully acknowledged. The funder had no role in study design, data collection and analysis, decision to publish, or preparation of the manuscript. |
| 6. Please include captions for your Supporting Information files at the end of your manuscript, and update any in-text citations to match accordingly. Please see our Supporting Information guidelines for more information: http://journals.plos.org/plosone/s/supporting-information. | Thank you. We included captions for Supporting Information files at the end of the manuscript. We updated these files and in-text citations. |
| 7. Please review your reference list to ensure that it is complete and correct. If you have cited papers that have been retracted, please include the rationale for doing so in the manuscript text, or remove these references and replace them with relevant current references. Any changes to the reference list should be mentioned in the rebuttal letter that accompanies your revised manuscript. If you need to cite a retracted article, indicate the article’s retracted status in the References list and also include a citation and full reference for the retraction notice. | We reviewed our reference list to ensure that it is complete and correct. We highlighted the changes we made in the reference list. We also checked all the papers we cited in the retraction database (<http://retractiondatabase.org/>) to ensure that none of them were retracted. |
